# 4-Diketopyrrole-2-carboxamide derivatives affecting HBV capsid assembly: structure–activity relationship around the GLP-26 scaffold

**DOI:** 10.1039/d6ob00943c

**Published:** 2026-10-05

**Authors:** Zhe Chen, Shaoman Zhou, Peng Liu, John Wiseman, Longhu Zhou, Chia-Han (Vanessa) Fan, Heather B. Bowers, Mohammad Salman, Kiran Verma, Danielle R. Monroe, Jessica Downs-Bowen, Dharmeshkumar Patel, Leda Bassit, Raymond F. Schinazi, Franck Amblard

**Affiliations:** a Center for ViroScience and Cure, Laboratory of Biochemical Pharmacology, Department of Pediatrics, Emory University School of Medicine, and Children's Healthcare of Atlanta Atlanta GA 30322 USA rschina@emory.edu famblar@emory.edu

## Abstract

Chronic hepatitis B virus (HBV) infection remains a major global health burden with limited curative options. Capsid assembly modulators (CAMs) have emerged as a promising antiviral strategy by disrupting nucleocapsid formation and suppressing cccDNA amplification. In this study, we discuss the structure–activity relationship (SAR) around the 4-diketopyrrole-2-carboxamide scaffold of GLP-26, focusing on modifying the aniline moiety and the oxoacetamide region, and introducing alternative acetamide groups. Compounds were synthesized through efficient coupling strategies, including microwave-assisted amidation, and several analogs exhibited potent inhibition of HBV DNA replication in HepAD38 cells, with EC_50_ values in the low nanomolar range and minimal cytotoxicity. This work broadens the chemical space surrounding GLP-26 and provides a valuable roadmap for designing new scaffolds for next-generation HBV CAMs.

## Abstract 2

SAR around HBV capsid assembly modulator GLP-26.

## Graphical abstract

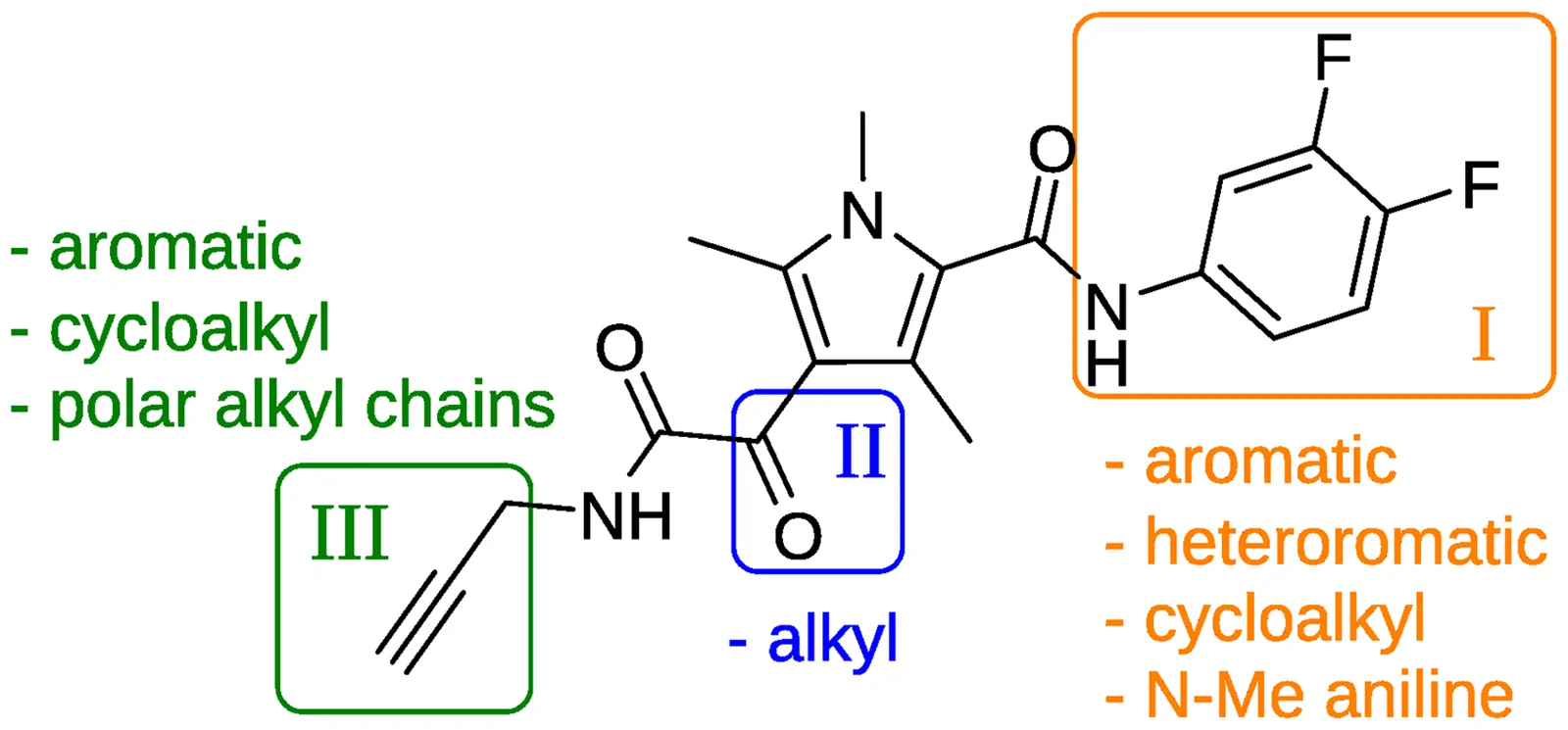

## 1. Introduction

Hepatitis B virus (HBV) is a major global health problem, with an estimated 350 million people infected worldwide.^1^ Chronic hepatitis B virus (CHB) infection is associated with cirrhosis and hepatocellular carcinoma (HCC) and is responsible for approximately 686 000 deaths per year.^2^ Although effective vaccines and antiviral treatments are available, there is still a need for new drugs for the treatment of HBV. At present, HBV treatment mainly relies on interferon and nucleoside analogs (NA). The former suppresses the virus by stimulating the immune system, while the latter, once triphosphorylated, inhibit HBV reverse transcriptase. Neither of these two types of therapies can completely resolve HBV infections.^3^ Tolerance of interferon is poor, and treatment rarely clears the virus from patients. NAs can only suppress the replication of HBV, but they do not cure HBV infection. Once the drugs are withdrawn, viral tiers often rebound to pre-treatment levels. Long-term antiviral therapy is often required to maintain viral suppression, and some patients may develop resistance to the drugs over time. Because the current treatments have limitations, there is a need for new, more effective, and well-tolerated HBV drugs that can cure HBV infection or provide long-lasting viral suppression with minimal side effects. These drugs could improve the quality of life for people with HBV and reduce the burden of HBV-related diseases worldwide. HBV capsid assembly modulators (CAMs) represent a promising class of direct-acting antivirals that target the viral core protein (HBc), a key structural component responsible for nucleocapsid formation. The HBV core protein plays a central role in viral replication by encapsidating pregenomic RNA (pgRNA) and viral polymerase, enabling reverse transcription and subsequent formation of relaxed circular DNA. By binding to a hydrophobic pocket at the dimer–dimer interface of the core protein, CAMs modulate capsid assembly.^4^ Over the past decade, major progress has been made in the discovery of HBV capsid assembly modulators (CAMs), resulting in several classes with diverse chemical structures. Recent reviews have summarized advances in CAM discovery, mechanisms of action, structure–activity relationships, and clinical development.^5,6^ CAM-A compounds, represented by heteroaryldihydropyrimidines (HAPs) such as Bay 41-4109,^7,8^ GLS4,^9,10^ RO7049389,^11^ and RG7907,^12^ induce aberrant capsid assembly. Of particular interest to the present study are CAM-E compounds, including NVR 3-778,^13^ AT-130,^14^ JNJ-56136379,^15^ ABI-H0731,^16^ AB-506,^17^ AB-836,^18^ ZM-H1505R,^19^ and QL-007,^20^ representative structures of which are shown in Fig. 1. These compounds promote the formation of empty capsids and inhibit HBV replication. Several CAMs have advanced into clinical trials and have shown encouraging antiviral activity, supporting this class of compounds as a promising therapeutic approach for chronic HBV infection. However, despite demonstrating antiviral activity, the clinical development of several CAM-E candidates has been discontinued or has not progressed further because of limitations related to insufficient efficacy at clinically feasible doses and liver safety concerns.^21,22^ These outcomes highlight both the therapeutic potential of the CAM-E mechanism and the continued opportunity to develop new CAM-E inhibitors with improved potency, and physicochemical, pharmacokinetic, and drug-like properties. Over the past several years, our laboratory has been actively engaged in the discovery and development of novel HBV CAMs and identified several 4-diketopyrrole-2-carboxamide derivatives.^23–26^ Among these 4-diketopyrrole-2-carboxamide derivatives, GLP-26 (Fig. 1) emerged as one of the most potent and promising compounds. GLP-26 inhibits HBV DNA production, HBeAg formation, and cccDNA establishment when used at low nanomolar concentrations, without displaying significant cytotoxicity across a panel of cell lines. Strikingly, long-term combination treatment with GLP-26 and entecavir in a humanized mouse model reduced viral loads and viral antigens, which were sustained for up to 12 weeks after treatment cessation.^27,28^ More interestingly, a recent study in an immunocompetent humanized mouse model of chronic HBV showed that treatment with GLP-26 clears viremia and HBsAg, reduces liver inflammation, and promotes anti-HBs antibody development. After stopping therapy, half of the mice maintained viral control with seroconversion and strong HBV-specific immune responses, suggesting that finite CAM treatment could enable durable off-therapy viral control consistent with a functional cure.^29^

**Fig. 1:**
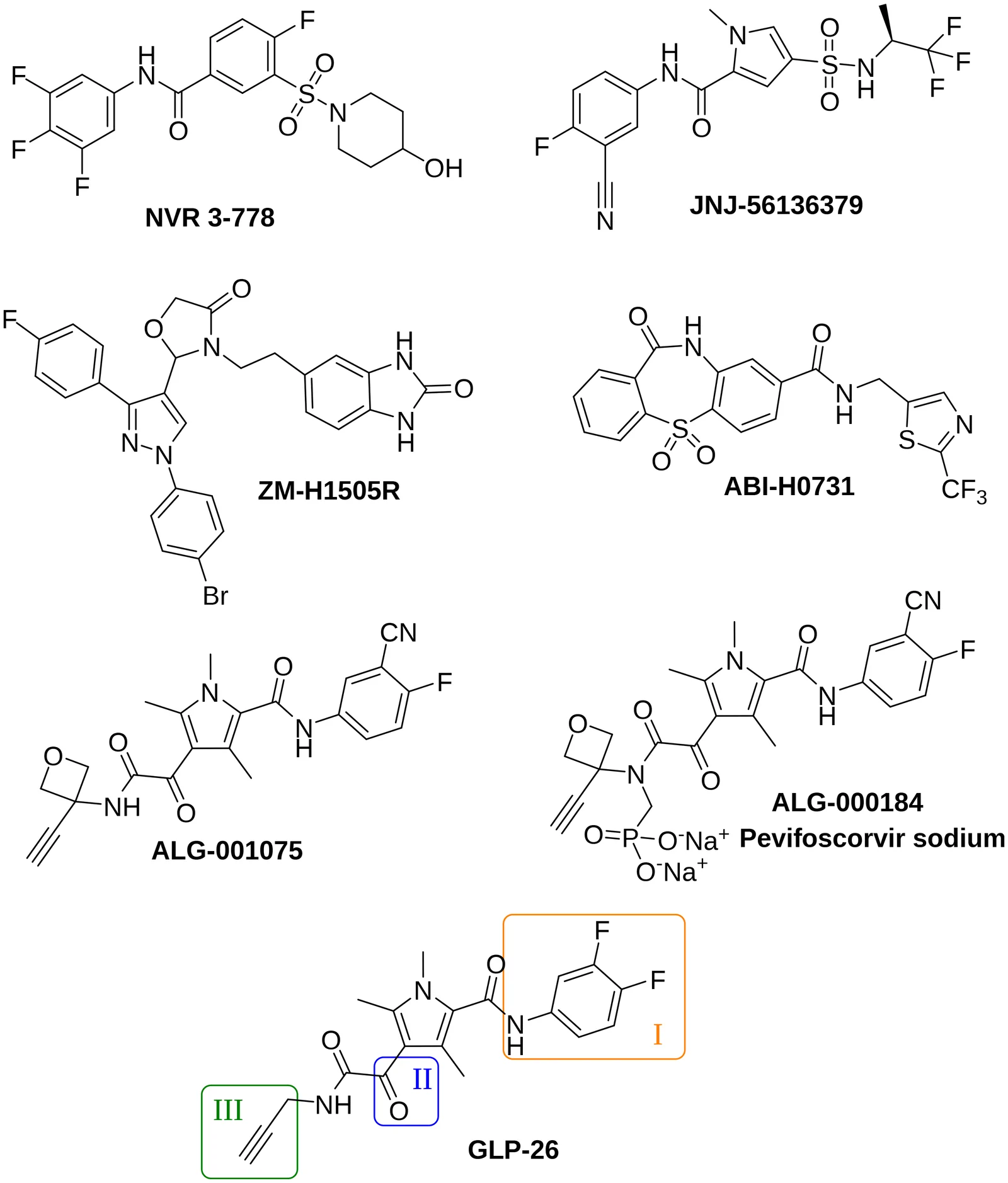
Structures of representative HBV CAM-E compounds and the proposed modifications around the GLP-26 scaffold.

In parallel, medicinal chemistry optimization of GLP-26 led to the discovery of a clinical candidate pevifoscorvir sodium (formerly ALG-000184), a highly soluble prodrug of ALG-001075, which has shown unprecedented reductions in viral DNA, RNA, and antigens in 96-week Phase 1b trials and Phase II trials (Fig. 1).^30–32^ The clinical advancement of ALG-000184 further demonstrates the therapeutic potential of this CAM-E chemical space. At the same time, the limitations encountered with earlier CAM-E candidates provide a strong rationale for continued exploration of the GLP-26 scaffold toward inhibitors with improved physicochemical, pharmacokinetic, and drug-like properties. Collectively, these findings highlight CAMs in general and GLP-26 analogs in particular as a validated therapeutic modality with strong potential to achieve a functional cure for chronic HBV infection.

In the present study, we describe our efforts to further explore the chemical space surrounding the GLP-26 scaffold and discuss the resulting structure–activity relationships (SAR).

## 2. Results and discussion

### 2.1. Chemical synthesis

To simplify the structure–activity relationship (SAR) study, the GLP-26 4-diketopyrrole-2-carboxamide scaffold was divided into three parts, labeled I, II and III (Fig. 1).

#### 2.1.1. Part I: replacement of the aniline moiety

The original 3,4-difluoroaniline moiety of GLP-26 was replaced with various substituted amines using the chemistry described in Schemes 1 and 2. The commercially available 1*H*-pyrrole-2-carboxylate 1 was first methylated by treatment with iodomethane in the presence of K_2_CO_3_ to obtain intermediate 2, which was then subjected to a Friedel–Crafts acylation with ethyl oxalyl chloride in the presence of aluminum chloride. The resulting compound 3 was selectively saponified using 5% aqueous sodium hydroxide in methanol, and the oxalic acid group formed 4 was further reacted with propargylamine in the presence of CDI to give 4-diketopyrrole-2-carboxylate 5 in 89% yield. Compound 5 was then treated with 5% aqueous sodium hydroxide in methanol and the acid 6 generated was reacted with various amines. The reaction of compound 6 with aliphatic amines in the presence of HATU and DIPEA was carried out to afford compounds 7a–7c in variable yields: 7a and 7b were obtained in moderate yields (65% and 71%, respectively), whereas 7c was isolated in a relatively low yield (31%). In contrast, the coupling reactions with aromatic amines required elevated temperatures or microwave irradiation to afford compounds 7d–7j in low to moderate yields (25–62%). For compounds 7k–7m, the above method was unsuccessful, and the desired compounds were finally obtained by treating carboxylic acid 6 with oxalyl chloride to first convert it into the corresponding acid chloride followed by coupling with the desired amine in the presence of DIPEA.

**Scheme 1:**
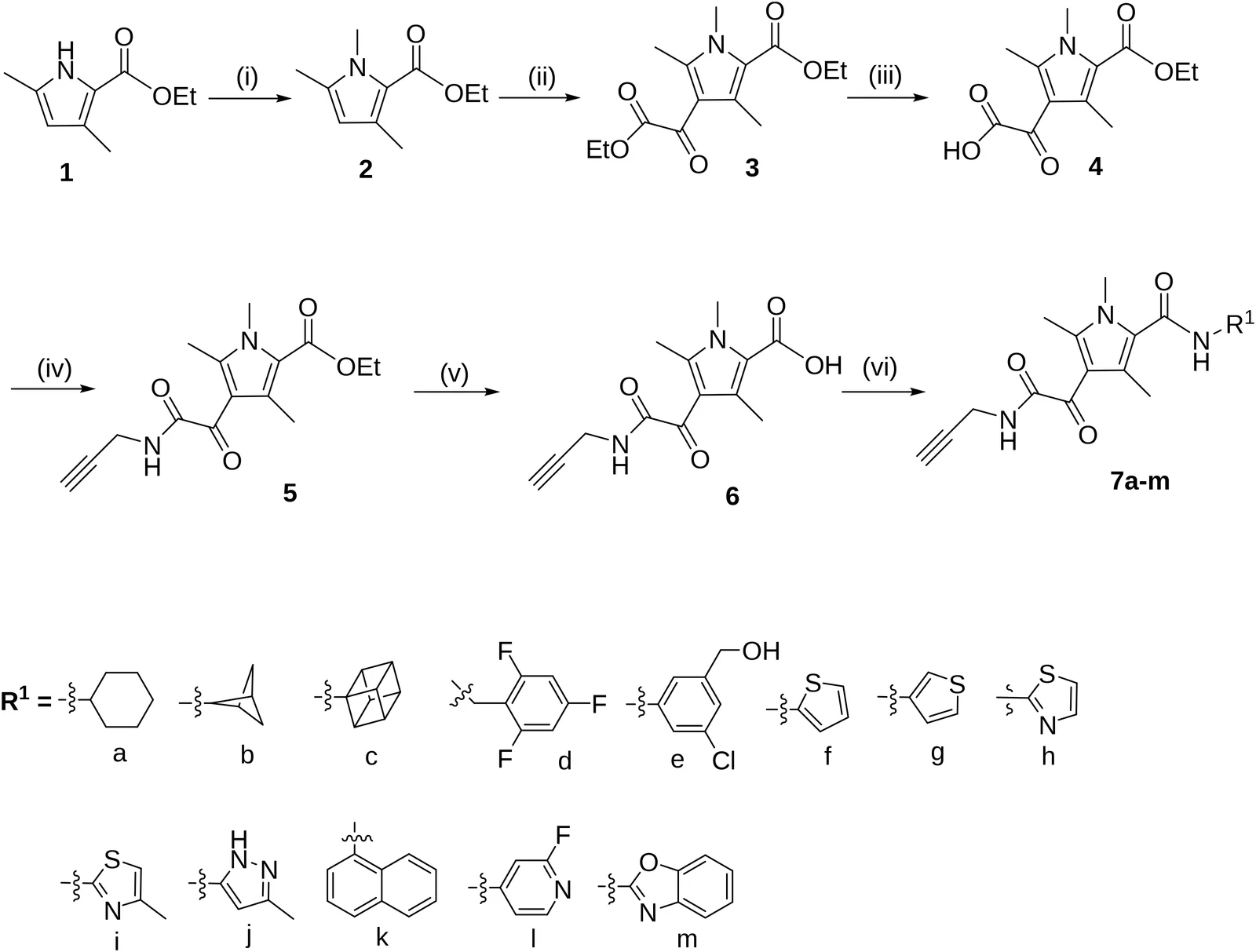
Reagents and conditions: (i) MeI, K_2_CO_3_, DMSO, rt, overnight, 92%; (ii) ClCOCO_2_Et, AlCl_3_, DCM, rt, overnight, 87%; (iii) 5% aq. NaOH, MeOH, rt, 15 min, 80%; (iv) propargylamine, CDI, DMF–DCM, rt, 3 h, 89%; (v) 5% aq. NaOH, MeOH–THF, rt, 16 h; then 1 N HCl to pH 3–4, 75%; (vi) R^1^-NH_2_, HATU, DIPEA, DMF, for compound 7a: 65 °C, 45 min, yield 65%; compound 7b: room temperature, overnight, yield 71%; compound 7c: 110 °C, overnight, yield 31%; compound 7d: microwave irradiation, 90 °C, 20 min, yield 80%; compounds 7e–7j: microwave irradiation, 65–70 °C, 35–40 min, yields 21–42%; and for compounds 7k–7m: (1) oxalyl chloride, DCM, 0 °C, 1 h; (2) R^1^-NH_2_, DIPEA, DCM, rt, 20 h, 30–60%.

**Scheme 2:**
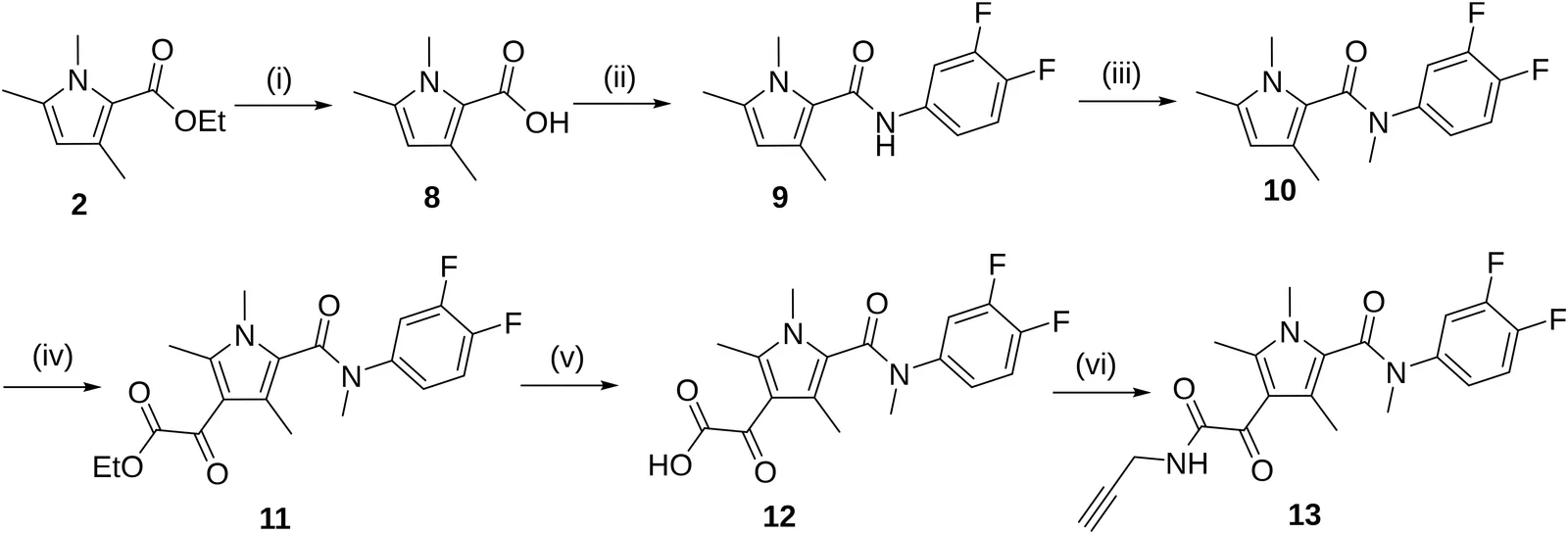
Reagents and conditions: (i) 5% aq. NaOH, MeOH–THF, rt, 16 h, 81%; (ii) 3,4-F-aniline, HATU, DIPEA, DMF, 65 °C, 16 h, 77%; (iii) KOH, MeI, DMSO, rt, overnight, 79%; (iv) ClCOCO_2_Et, AlCl_3_, DCM, rt, overnight, 82%; (v) 5% aq. NaOH, MeOH, rt, 15 min; then 1 N HCl to pH 3–4; and (vi) propargylamine, CDI, DMF–DCM, rt, 3 h, 65% over two steps.

The *N*-methyl analog of GLP-26, compound 13, was prepared according to the chemistry described in Scheme 2. Pyrrolylcarboxylate 2 was first saponified with sodium hydroxide to give the corresponding carboxylic acid 8, which was subsequently coupled with 3,4-difluoroaniline in the presence of HATU and DIPEA to afford amide 9. Alkylation of 9 with iodomethane in DMSO furnished *N*,*N*-disubstituted amide 10, which was then acylated with ethyl oxalyl chloride in the presence of AlCl_3_ to afford compound 11 in high yield. Oxoacetate 11 was saponified to the corresponding carboxylic acid 12, which was subsequently condensed with propargylamine in the presence of CDI to afford the desired product 13 in 65% overall yield over two steps.

#### 2.1.2. Part III: modifications of the oxoacetamide moiety

Compounds 16a–i and 17 were synthesized following the route outlined in Scheme 3. Compound 9 was acylated with ethyl oxalyl chloride under Friedel–Crafts conditions to afford oxoacetate 14, which was subsequently saponified with 5% aqueous sodium hydroxide to generate the corresponding diketo acid 15. Condensation of 15 with various amines (R^a–k^–NH_2_) in the presence of HATU and DIPEA provided the desired amide derivatives 16a–i in good yields. Compound 17 was prepared from intermediate 16i by treatment with phenylboronic acid in Et_2_O followed by 1 N HCl/H_2_O at 50 °C for 16 h (70% yield). Compound 18 was prepared from GLP-26 by triple bond migration under basic conditions (20% aqueous NaOH at 100 °C) (Scheme 4).

**Scheme 3:**
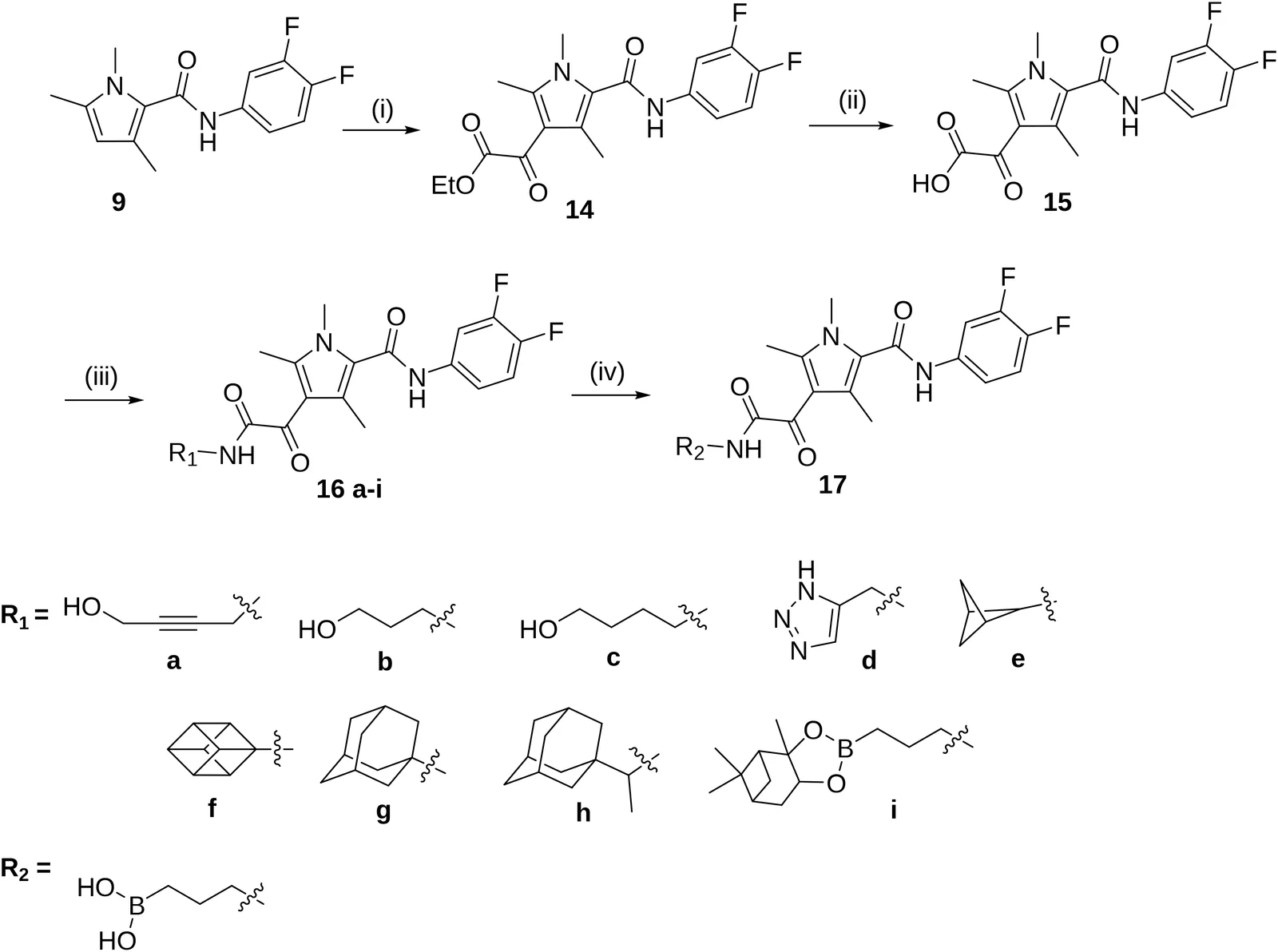
Reagents and conditions: (i) ClCOCO_2_Et, AlCl_3_, DCM, rt, overnight, 79%; (ii) 5% NaOH, MeOH, rt, 15 min; then 1 N HCl to pH 3–4; (iii) R_1_-NH_2_, HATU, DMF, DIPEA, 60–80 °C, overnight; and (iv) 16i, 1 N HCl, PhB(OH)_2_, Et_2_O–H_2_O, 50 °C, 16 h.

**Scheme 4:**
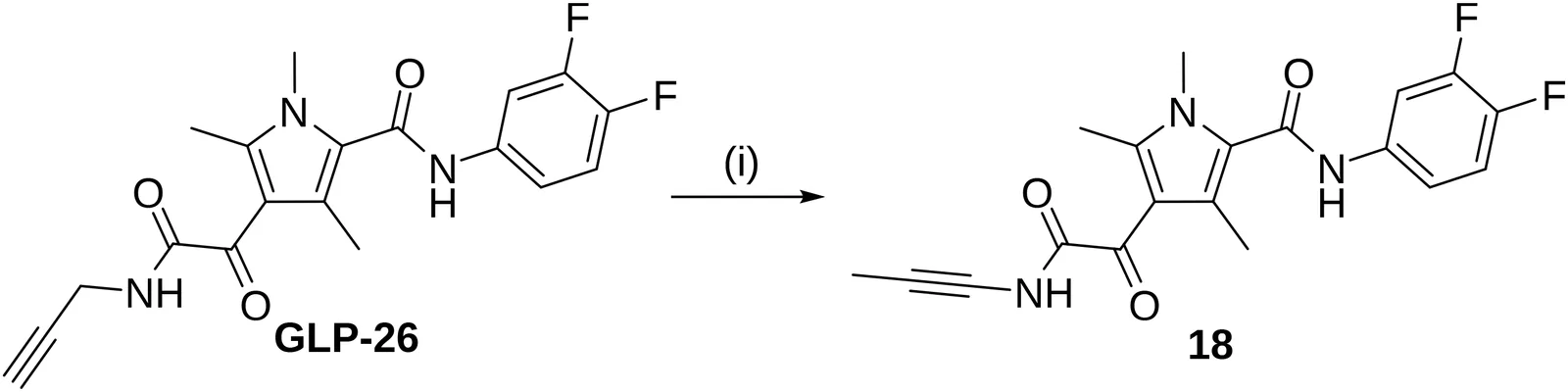
Reagents and conditions: (i) 20% NaOH, EtOH, 100 °C, 24 h, 83%.

#### 2.1.3. Part II: replacement of oxoacetamide with an acetamide group

Compound 19 was synthesized following the procedure reported by Timothy D. Lash and co-workers.^33^ Methylation of compound 19 afforded compound 20. Subsequent selective hydrolysis of the ethyl ester using 1 N sodium hydroxide in THF–MeOH afforded the corresponding carboxylic acid derivative 21. Compound 21 was then coupled with propargylamine using HATU and DIPEA in DMF to yield amide 22, which was subsequently condensed with 3,4-difluoroaniline in the presence of LiHMDS in THF at 60 °C, providing compound 23 in high yield (Scheme 5).

**Scheme 5:**
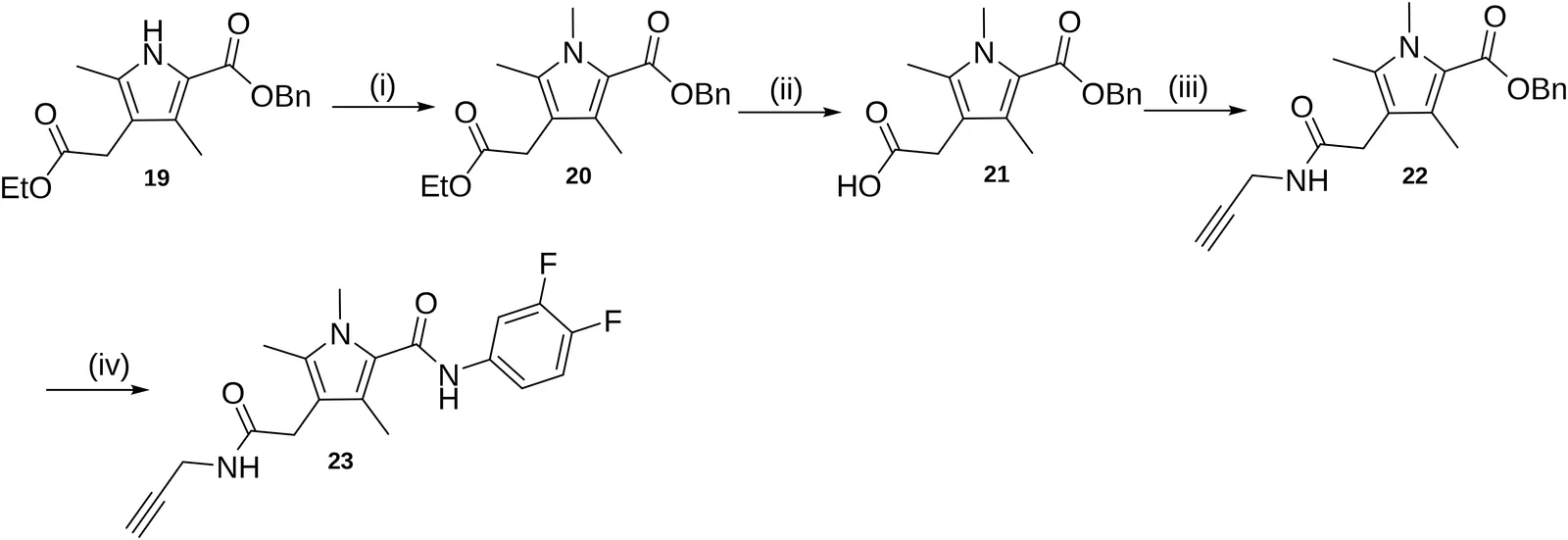
Reagents and conditions: (i) MeI, KOH, DMSO, rt, overnight, 66%; (ii) 1 N NaOH, THF–MeOH (1 : 1), rt; then 1 N HCl to pH 3–4, 86%; (iii) propargylamine, HATU, DIPEA, DMF, 65 °C, 3 h, 60%; and (iv) 3,4-difluoroaniline, LiHMDS, THF, 40 °C, 1 h, 70%.

### 2.2. Structure–activity relationship and discussion

The anti-HBV activity (HBV DNA production) of all synthesized compounds was tested in HepAD38 cells,^26,27^ and the cytotoxicity profile was measured in human lymphocytes (PBM), T lymphoblast cells (CEM), African Green Monkey cells (Vero), and human hepatoma cells (HepG2). All the data are summarized in Tables 1 and 2.

**Table 1:** Anti-HBV activity in HepAD38 cells and cytotoxicity profiles of compounds 7a–m and 13

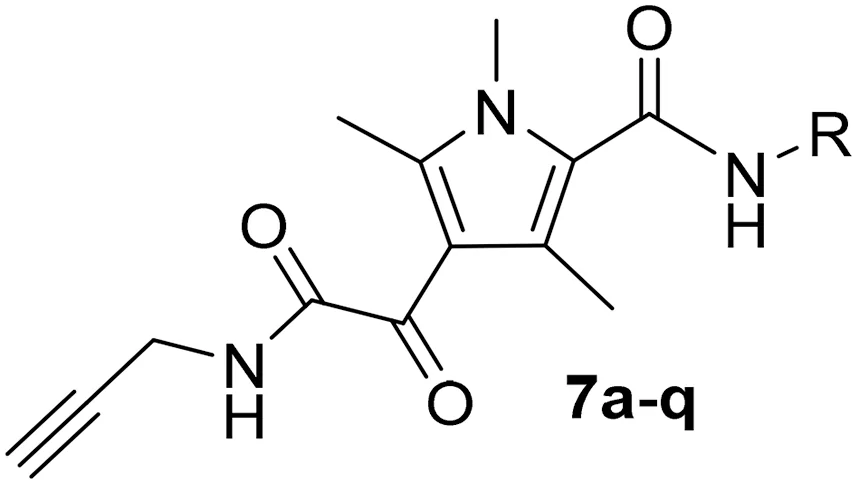						
Compound	R	Anti-HBV activity EC_50_ and EC_90_ (nM)	Cytotoxicity CC_50_ (μM)			
			PBM	CEM	Vero	HepG-2
7a	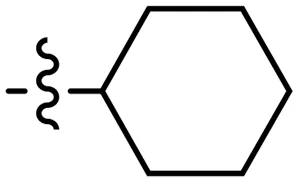	668 ± 103	>100	>100	>100	>100
		4461 ± 2095				
7b	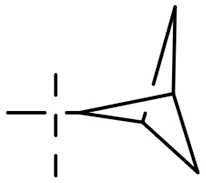	>10 000	>100	>100	>100	>100
		>10 000				
7c	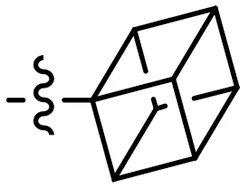	4655 ± 728	>100	>100	>100	>100
		9380 ± 156				
7d	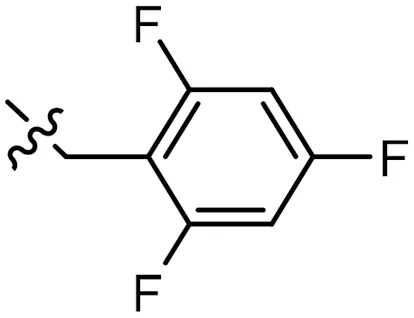	>10 000	>100	>100	>100	>100
		>10 000				
7e	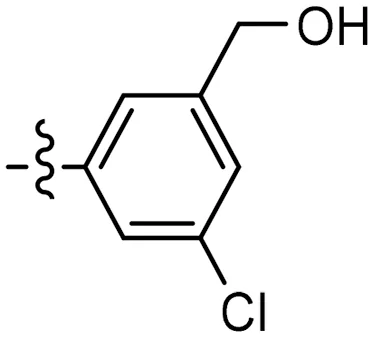	30 ± 14	>100	>100	>100	>100
		315 ± 163				
7f	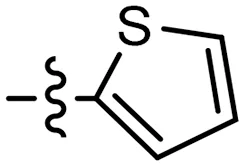	>10 000	>100	>100	>100	>100
		>10 000				
7g	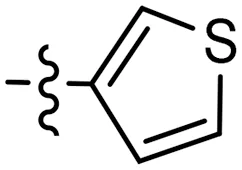	2062 ± 2234	>100	>100	>100	>100
		8005 ± 2196				
7h	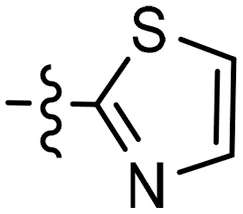	>10 000	>100	>100	>100	>100
		>10 000				
7i	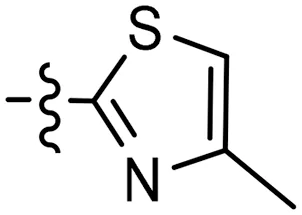	6568 ± 2531	>100	>100	>100	>100
		>10 000				
7j	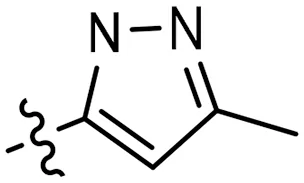	>10 000	>100	>100	>100	>100
		>10 000				
7k	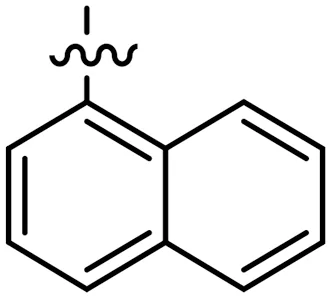	471 ± 245	>100	>100	>100	>100
		3988 ± 917				
7l	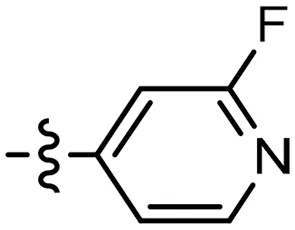	2436 ± 1603	>100	>100	>100	>100
		9538 ± 1332				
7m	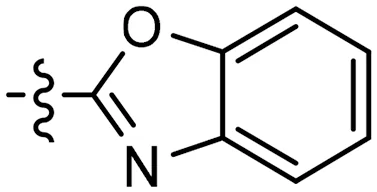	3690 ± 3691	>100	>100	>100	>100
		8940 ± 3711				
13	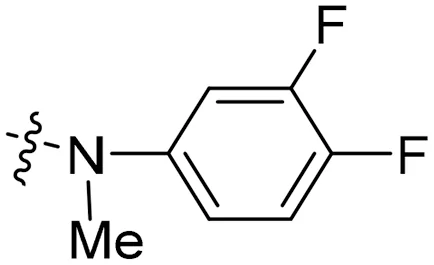	>10 000	>100	>100	>100	>100
		>10 000				
GLP-26	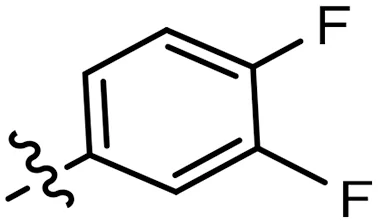	2.67 ± 1.15	>100	>100	91	>100
		30.0 ± 26.5				

**Table 2:** Anti-HBV activity in HepAD38 cells and cytotoxicity profiles of compounds 16a–h, 17, 18, and 23

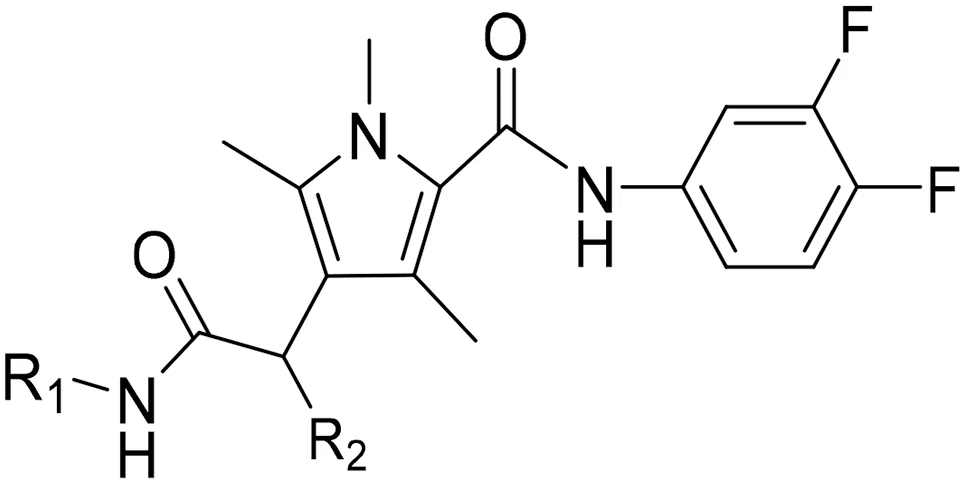							
Compound	R_1_	R_2_	Anti-HBV activity EC_50_ and EC_90_ (nM)	Cytotoxicity CC_50_ (μM)			
				PBM	CEM	Vero	HepG-2
16a	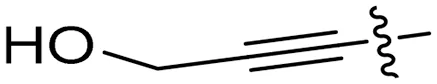	<svg xmlns="http://www.w3.org/2000/svg" version="1.0" width="13.200000pt" height="16.000000pt" viewBox="0 0 13.200000 16.000000" preserveAspectRatio="xMidYMid meet"><metadata> Created by potrace 1.16, written by Peter Selinger 2001-2019 </metadata><g transform="translate(1.000000,15.000000) scale(0.017500,-0.017500)" fill="currentColor" stroke="none"><path d="M0 440 l0 -40 320 0 320 0 0 40 0 40 -320 0 -320 0 0 -40z M0 280 l0 -40 320 0 320 0 0 40 0 40 -320 0 -320 0 0 -40z"/></g></svg> O	12.65 ± 7.99	>100	>100	>100	>100
			107 ± 61				
16b	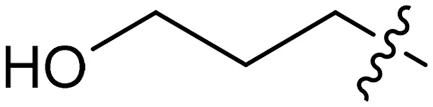	O	8.9 ± 0.1	>100	>100	>100	>100
			54 ± 23				
16c	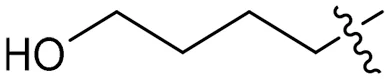	O	24 ± 15	≥100	>100	>100	>100
			314 ± 44				
16d	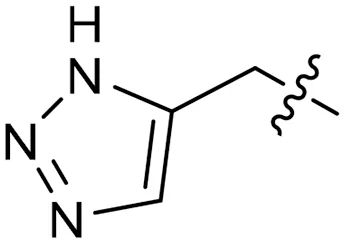	O	9.4 ± 0.2	51 ± 16	47 ± 16	>100	>100
			276 ± 28				
16e	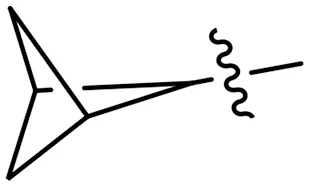	O	4.3 ± 1.3	83 ± 14	56 ± 15	>100	74 ± 10
			23.4 ± 21.2				
16f	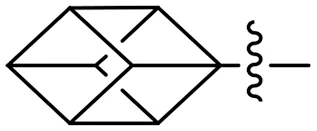	O	6.3 ± 3.3	19 ± 14	12 ± 1	19 ± 17	29 ± 15
			34.5 ± 25.4				
16g	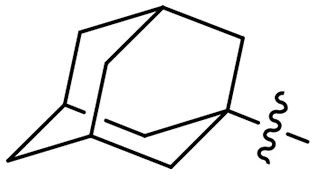	O	19 ± 10	>100	ND	>100	>100
			50 ± 34				
16h	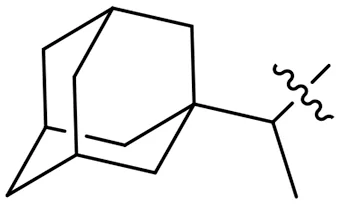	O	35 ± 27	>100	>100	>100	>100
			66 ± 33				
17	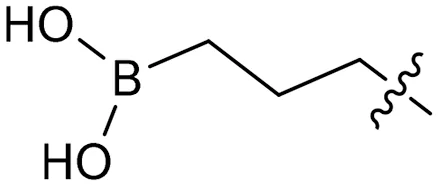	O	15 ± 4	>100	62 ± 32	>100	>100
			77 ± 24				
18	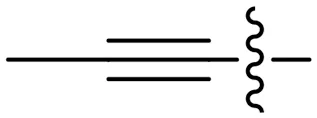	O	514 ± 181	78.6 ± 9.40	>100	>100	>100
			4706 ± 3426				
23	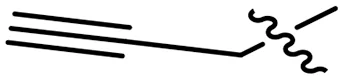	H	>10 000	69 ± 45	30 ± 6	>100	31 ± 11
			>10 000				
GLP-26	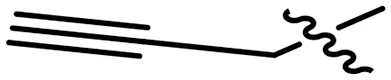	O	2.67 ± 1.15	>100	>100	91	>100
			30.0 ± 26.5				

#### 2.2.1. Docking-guided design and SAR strategy

In the absence of an experimentally determined GLP-26–HBV core protein co-crystal structure, our predicted binding model (Fig. 2) served as a structural framework for the present medicinal chemistry study.^25^ The model was used to identify key ligand–protein interactions, define regions likely to be important for binding, and guide the selection of positions for systematic structural modification. Based on these predictions, the GLP-26 scaffold was divided into three key regions (Parts I–III, Fig. 1) for systematic exploration, allowing the predicted binding interactions and structural tolerance of each region to be assessed through experimental SAR.

**Fig. 2:**
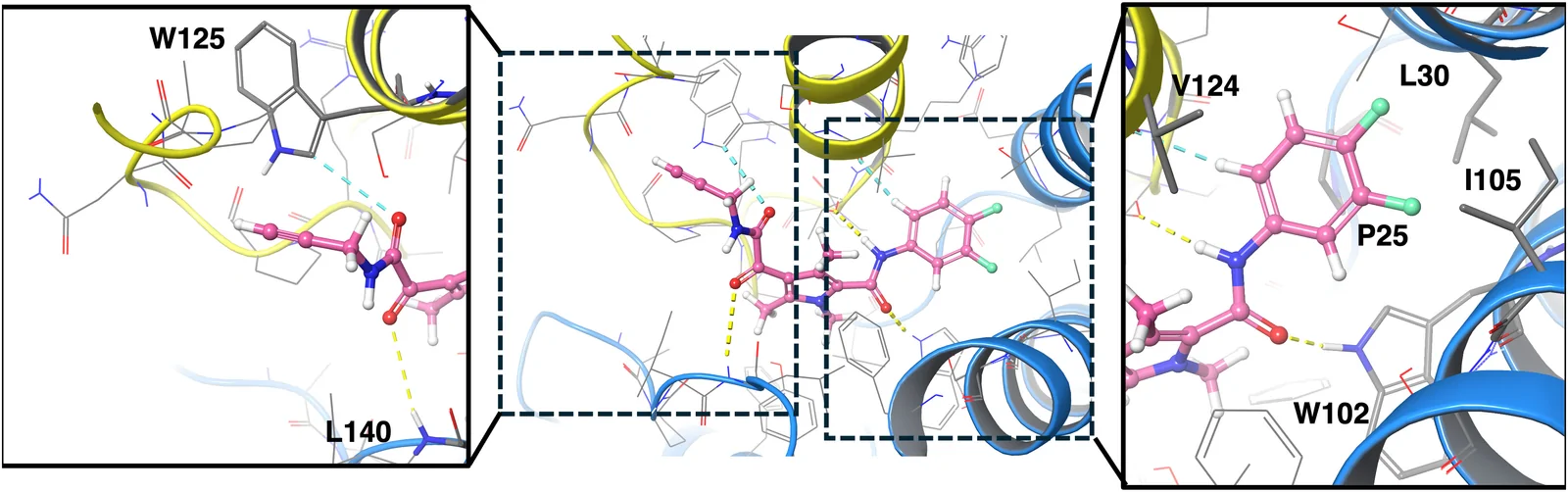
Predicted binding mode of GLP-26 with the HBV core protein dimer. The zoomed view highlights the interactions of the phenyl ring and the diketo solvent-exposed region with the HBV core protein. The phenyl ring forms hydrophobic interactions with I105, L30, P25, and W102, an edge-to-face π–π interaction with W102, and an aromatic hydrogen bond with V124. The diketo moiety forms hydrogen bonds with L140 and an aromatic hydrogen bond with W125. (Aromatic hydrogen bonds are shown as cyan dashed lines; hydrogen bonds are shown as yellow dashed lines.)

#### 2.2.2. Part I modifications

As anticipated based on our predicted binding mode of GLP-26 with the HBV core protein dimer (Fig. 2), *N*-methylation of the aniline moiety in GLP-26 (compound 13) was detrimental to the potency of this series, confirming that the free amide group plays a critical role in compound binding as it is involved in the H-bond interactions with W102 and T128, suggesting that these are essential H-bond interactions for the anti-HBV activity. Given that the 3,4-difluorophenyl ring of GLP-26 occupies a well-defined hydrophobic pocket, we sought to determine whether this moiety could be replaced with smaller five-membered aromatic bioisosteres while maintaining antiviral activity. Introduction of thiophene analogs (7f and 7g), thiazole derivatives (7h and 7i), or a pyrazole analog (7j) resulted in a clear loss of potency, suggesting that the hydrophobic pocket does not simply tolerate aromaticity but instead requires the specific size, shape, and electronic properties of the phenyl ring. Similarly, incorporation of larger bicyclic aromatic systems (7k and 7m) or a trifluorobenzyl substituent (7d) also led to diminished activity, indicating that both contraction and expansion of the aromatic scaffold are detrimental to target engagement. These findings suggest that the pocket is highly optimized for accommodation of the 3,4-difluorophenyl group and has limited tolerance for significant changes in steric footprint.

Among the analogs evaluated, compound 7e, bearing a 3-chloro-5-(hydroxymethyl)phenyl substituent, was the most active and exhibited only an 11-fold reduction in potency relative to GLP-26. The retention of measurable activity with 7e further supports the importance of preserving the phenyl core while allowing modest substitution around the ring periphery. In contrast, replacement of the aromatic ring with hydrophobic saturated motifs, including cyclohexyl (7a) or the phenyl bioisosteres cubane (7c) and bicyclo[1.1.1]pentane (7b), also resulted in reduced potency. Although these substituents are expected to maintain favorable hydrophobic contacts with residues lining the pocket, including W102, P25, I105, L30, and V124, they lack the aromatic character required for key binding interactions.

Collectively, these SAR results highlight the critical role of the 3,4-difluorophenyl ring in GLP-26. Beyond serving as a hydrophobic anchor, the phenyl ring appears to provide an optimal balance of size, planarity, and electronic properties necessary for productive binding. Structural modeling suggests that this ring participates in a favorable edge-to-face π-stacking interaction with W102 and may also engage in a weak aromatic C–H⋯O interaction with the backbone carbonyl of V124 (Fig. 2). The inability of smaller heteroaromatic rings, larger bicyclic systems, or saturated bioisosteres to effectively reproduce these interactions likely accounts for their reduced activity, underscoring the importance of the phenyl ring as a key pharmacophoric element of GLP-26.

#### 2.2.3. Part II and III modifications

Our binding prediction model (Fig. 2) showed that the diketo group in GLP-26 provides key interactions with the HBV core protein dimer: the carbonyl next to the pyrrole ring forms an H-bond with the backbone –NH of the L140 residue, while the second carbonyl is involved in aromatic H-bond interactions with residue W125 (Fig. 2). To experimentally validate the predicted importance of these interactions and assess whether this region could tolerate structural modification, we selected the diketo moiety as one of the key positions for SAR exploration. As predicted by our model, replacing one of the keto groups with a simple alkyl substituent (compound 23) resulted in a loss of potency. This result supported the predicted importance of the carbonyl group for antiviral activity; therefore, no further modifications at this position were pursued. In contrast, because the alkyne moiety of GLP-26 is predicted to be solvent-exposed, substitutions at this position were expected to be well tolerated. Guided by our binding model and supported by previous observations,^22,23^ this solvent-exposed region was selected for further structural exploration. Accordingly, the introduction of alkyl chains bearing polar hydroxyl groups (16a, 16b, and 16c) or a boronic acid group (17) led to only minimal losses in potency compared to GLP-26. Similarly, incorporation of bulkier substituents such as a triazole (16d), cubane (16f), bicyclopentane (16e), or adamantane derivatives (16g and 16h) was also well tolerated, with 16f and 16g displaying EC_50_ values comparable to that of GLP-26. Interestingly, isomerization of the triple bond in GLP-26, as shown in compound 18, proved counterproductive and resulted in an approximately 190-fold reduction in potency. Docking of compound 18 with the HBV core protein dimer suggests that the triple bond alters the conformation of the solvent-exposed region and may create steric clashes with residues W125 and P134 (Fig. 3), potentially explaining the loss of potency.

**Fig. 3:**
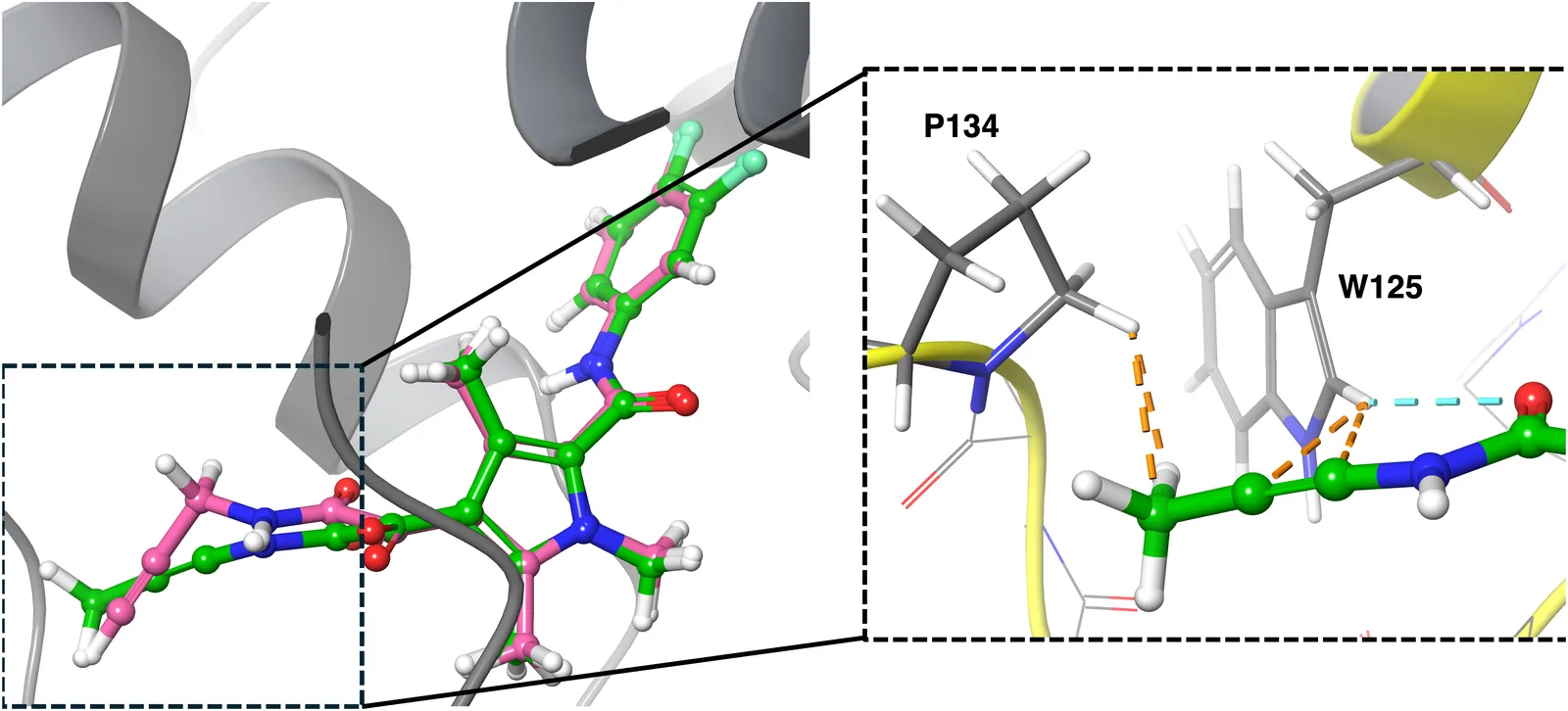
Superimposition of compound 18 onto the docked pose of GLP-26. Compound 18 is shown in green, and GLP-26 is shown in magenta. The zoomed view highlights steric clashes between compound 18 and residues P134 and W125 of the HBV core protein (orange dashed lines).

## 3. Conclusion

A series of GLP-26 analogs were synthesized and evaluated to better understand the key features around the GLP-26 scaffold. In the absence of an experimentally determined GLP-26–HBV core protein co-crystal structure, our docking-based binding model provided an important structural framework for rational analog design and selection of key regions for medicinal chemistry exploration. The resulting analogs were synthesized and biologically evaluated, allowing the predicted binding interactions to be systematically assessed through experimental SAR. Our SAR analysis confirmed the importance of several structural elements for an optimal activity profile, including: (1) the phenyl ring in the aniline moiety, (2) hydrogen bonding involving the aniline NH group, and (3) key hydrogen-bonding interactions associated with the diketo motif. In particular, modifications that disrupted predicted key interactions resulted in corresponding changes in antiviral activity, providing experimental support for the proposed binding model. We also showed that the solvent-exposed portion of the molecule (Part III) is relatively permissive, with a wide variety of modifications being well tolerated.

While several of the compounds described exhibit single-digit nanomolar activity comparable to that of GLP-26, the data generated in this study provide new insights into the structural determinants governing activity within this class of HBV CAMs. Collectively, the integration of molecular docking, chemical synthesis, and biological evaluation provides a useful SAR framework for guiding further optimization toward next-generation HBV CAMs with improved potency.

## 4. Experimental section

### 4.1. Chemistry

Commercially available chemicals were of reagent grade and used as received. Nuclear magnetic resonance (NMR) spectra (^1^H, ^13^C and ^19^F) were recorded on a Bruker Ascend™ 400 MHz Fourier transform spectrometer at rt, with tetramethylsilane (TMS) as an internal standard. Chemical shifts (*δ*) are reported in parts per million (ppm) and signals are quoted as s (singlet), d (doublet), t (triplet), q (quartet), m (multiplet), bs (broad signal), dd (doublet of doublets) or ddd (doublet of doublets of doublets). ^13^C NMR data are reported as observed, that is, some carbon signals overlap with solvent signals. High-resolution mass spectra (HRMS) were recorded on a ThermoFisher Q exactive Plus high-resolution mass spectrometer with electrospray ionization (ESI). Analytic TLC was performed on Analtech GHLF silica gel plates, and preparative TLC on Analtech GF silica gel plates. Column chromatography was performed on Combiflash R_f_200 or *via* reverse-phase high performance liquid chromatography. Reactions under microwave irradiation were performed on a CEM Discovery SP Microwave Synthesizer using 5 mL sealed tubes. Cell sources: human peripheral blood mononuclear cells (PBMCs) were isolated from buffy coats obtained from the New York Blood Center (601 Midland Ave., Rye, NY 10580, USA) using SepMate™ PBMC Isolation Tubes (STEMCELL Technologies) according to the manufacturer's protocol; human T lymphoblast (CEM), African green monkey kidney (Vero), and human hepatocellular carcinoma (HepG2) cell lines were obtained from the American Type Culture Collection (ATCC).

#### 4.1.1. General synthetic procedure for compounds 7a–j

Compound 6 was synthesized according to the literature procedure.^23^

To a solution of compound 6 (78 mg, 0.30 mmol, 1.0 eq.) in DMF (6 mL) were added the corresponding amine (0.45 mmol, 1.5 eq.), HATU (205 mg, 0.54 mmol, 1.8 eq.), and DIPEA (0.13 mL, 0.75 mmol, 2.5 eq.). The reaction mixture was stirred under the following conditions depending on the amine used: 7a: 65 °C, 45 min, yield 65%; 7b: room temperature, overnight, yield 71%; 7c: 110 °C, overnight, yield 31%; 7d: microwave irradiation, 90 °C, 20 min, yield 80%; 7e–7j: microwave irradiation, 65–70 °C, 35–40 min, yields 21–42%. After completion of the reaction, the reaction mixture was poured into 1 N HCl (20 mL) and extracted with EtOAc (3 × 40 mL). The combined organic layers were dried over anhydrous Na_2_SO_4_, filtered, and concentrated under reduced pressure. The crude residue was purified by flash column chromatography on silica gel using a gradient of hexane/ethyl acetate (100 : 1 → 0 : 100) to afford compounds 7a–7j as pure products.

##### 4.1.1.1. N-Cyclohexyl-1,3,5-trimethyl-4-(2-oxo-2-(prop-2-yn-1-ylamino)acetyl)-1H-pyrrole-2-carboxamide (7a)

Yield 65%: ^1^H NMR (400 MHz, CDCl_3_) *δ* 6.99 (s, 1 H), 5.61 (d, *J* = 8.1 Hz, 1 H), 4.16 (dd, *J* = 5.6, 2.6 Hz, 2 H), 3.96 (dd, *J* = 11.5, 7.3 Hz, 1 H), 3.65 (s, 3 H), 2.37 (s, 3 H), 2.34–2.20 (m, 4 H), 2.08–1.95 (m, 2 H), 1.82–1.55 (m, 4 H), 1.44 (ddd, *J* = 25.0, 9.2, 3.4 Hz, 2 H), 1.34–1.15 (m, 2 H); ^13^C NMR (101 MHz, CDCl_3_) *δ* 186.2, 163.9, 161.1, 141.1, 126.8, 121.7, 117.4, 78.6, 72.2, 48.4, 33.3, 32.2, 29.2, 25.5, 24.8, 12.3, 12.2; HRMS (ESI+) calcd for C_19_H_25_N_3_O_3_ + H_1_ 344.1896, found 344.1904.

##### 4.1.1.2. N-((1S,3S)-Bicyclo[1.1.1]pentan-2-yl)-1,3,5-trimethyl-4-(2-oxo-2-(prop-2-yn-1-ylamino)acetyl)-1H-pyrrole-2-carboxamide (7b)

Yield 71%: ^1^H NMR (400 MHz, CDCl_3_) *δ* 7.04–6.94 (m, 1H), 6.04 (s, 1H), 4.16 (dd, *J* = 5.6, 2.5 Hz, 2H), 3.66 (s, 3H), 2.51 (s, 1H), 2.36 (s, 3H), 2.29 (t, *J* = 2.7 Hz, 1H), 2.27 (s, 3H), 2.17 (s, 6H); ^13^C NMR (101 MHz, CDCl_3_) *δ* 186.2, 163.8, 162.1, 141.4, 126.3, 122.4, 117.4, 78.5, 72.2, 53.0, 48.7, 32.3, 29.2, 25.0, 12.4, 12.2; HRMS (ESI): *m*/*z* [M + H]^+^ calcd for C_18_H_22_N_3_O_3_: 328.1661 found: 328.1659.

##### 4.1.1.3. N-(Cuban-1-yl)-1,3,5-trimethyl-4-(2-oxo-2-(prop-2-yn-1-ylamino)acetyl)-1H-pyrrole-2-carboxamide (7c)

Yield 31%: ^1^H NMR (400 MHz, CDCl_3_) *δ* 7.02 (s, 1H), 6.22 (s, 1H), 4.23–4.21 (m, 3H), 4.18–4.16 (m, 2H), 4.02–4.00 (m, 4H), 3.69 (s, 3H), 2.37 (s, 3H), 2.32 (s, 3H), 2.94 (t, *J* = 2.6 Hz, 1H); ^13^C NMR (101 MHz, CDCl_3_) *δ* 186.2, 163.7, 161.1, 141.4, 125.9, 122.8, 117.5, 78.5, 72.2, 66.7, 52.8, 47.8, 43.0, 32.4, 29.2, 12.6, 12.2; HRMS (ESI): *m*/*z* [M + H]^+^ calcd for C_21_H_22_N_3_O_3_: 364.1661, found: 364.1642.

##### 4.1.1.4. 1,3,5-Trimethyl-4-(2-oxo-2-(prop-2-yn-1-ylamino)acetyl)-N-(2,4,6-trifluorobenzyl)-1H-pyrrole-2-carboxamide (7d)

Yield 80%: ^1^H NMR (400 MHz, DMSO-*d*_6_) *δ* 9.10 (t, *J* = 5.3 Hz, 1H), 8.63 (t, *J* = 4.5 Hz, 1H), 7.22–7.18 (m, 2H), 4.43 (d, *J* = 4.6 Hz, 2H), 3.98 (d, *J* = 5.2 Hz, 2H), 3.51 (s, 3H), 3.17 (s, 1H), 2.34 (s, 3H), 2.10 (s, 3H). ^13^C NMR (101 MHz, DMSO-*d*_6_) *δ* 187.7, 167.7, 163.0, 161.6, 160.6, 140.5, 127.2, 121.4, 116.3, 111.3, 100.9, 80.6, 73.8, 32.2, 31.1, 28.0, 11.6. ^19^F NMR (377 MHz, DMSO-*d*_6_) *δ* −109.0 to −109.1 (m, 1 F), −111.4 to −111.5 (m, 1F). HRMS (ESI) *m*/*z* calcd for C_20_H_19_F_3_N_3_O_3_ [M + H]^+^: 406.1379; found: 406.1373.

##### 4.1.1.5. N-(3-Chloro-5-(hydroxymethyl)phenyl)-1,3,5-trimethyl-4-(2-oxo-2-(prop-2-yn-1-ylamino)acetyl)-1H-pyrrole-2-carboxamide (7e)

Yield 40%: ^1^H NMR (400 MHz, MeOD) *δ* 7.75 (s, 1 H), 7.64–7.40 (m, 1 H), 7.17 (dd, *J* = 1.9, 1.4 Hz, 1 H), 4.63 (s, 1 H), 4.61 (s, 2 H), 4.12 (d, *J* = 2.5 Hz, 2 H), 3.67 (s, 3 H), 3.33 (dd, *J* = 3.2, 1.6 Hz, 2 H), 2.68 (dd, *J* = 4.3, 1.7 Hz, 1 H), 2.47 (s, 3 H), 2.35 (s, 3 H); ^13^C NMR (101 MHz, CDCl_3_) *δ* 186.5, 162.4, 157.6, 151.8, 145.4, 141.0, 136.5, 135.0, 129.5, 120.9, 120.0, 116.0, 78.3, 72.4, 33.5, 29.4, 13.6, 12.8; HRMS (ESI+) calcd for C_20_H_20_ClN_3_O_4_ + H_1_ 402.1142, found 402.1134.

##### 4.1.1.6. 1,3,5-Trimethyl-4-(2-oxo-2-(prop-2-yn-1-ylamino)acetyl)-N-(thiophen-2-yl)-1H-pyrrole-2-carboxamide (7f)

Yield 42%: ^1^H NMR (400 MHz, CDCl_3_) *δ* 8.74 (dd, *J* = 4.5, 1.4 Hz, 1 H), 8.45 (dd, *J* = 8.4, 1.4 Hz, 1 H), 7.45 (dt, *J* = 12.8, 6.4 Hz, 1 H), 7.12 (t, *J* = 5.4 Hz, 1 H), 4.24–4.15 (m, 2 H), 3.85 (d, *J* = 6.1 Hz, 3 H), 2.71–2.63 (m, 3 H), 2.48–2.42 (m, 3 H), 2.35–2.29 (m, 1H); ^13^C NMR (101 MHz, CDCl_3_) *δ* 186.5, 162.4, 157.6, 151.8, 145.3, 141.0, 136.5, 135.0, 129.5, 120.9, 116.0, 78.3, 72.4, 33.5, 29.4, 13.6, 12.8; HRMS (ESI+) calcd for C_17_H_17_N_3_O_3_S + H_1_ 344.0991, found 344.0985.

##### 4.1.1.7. 1,3,5-Trimethyl-4-(2-oxo-2-(prop-2-yn-1-ylamino)acetyl)-N-(thiophen-3-yl)-1H-pyrrole-2-carboxamide (7g)

Yield 31%: ^1^H NMR (400 MHz, CDCl_3_) *δ* 7.69 (s, 1 H), 7.64 (d, *J* = 1.9 Hz, 1 H), 7.29 (dd, *J* = 5.2, 3.2 Hz, 1 H), 7.05 (dd, *J* = 5.2, 1.3 Hz, 1 H), 7.01 (s, 1 H), 4.17 (dd, *J* = 5.6, 2.6 Hz, 2 H), 3.72 (s, 3 H), 2.41 (s, 3 H), 2.38 (s, 3 H), 2.30 (t, *J* = 2.6 Hz, 1 H); ^13^C NMR (101 MHz, CDCl_3_) *δ* 189.5, 163.6, 158.9, 151.3, 145.1, 142.0, 135.1, 124.9, 121.0, 118.1, 110.4, 78.5, 72.3, 32.4, 29.3, 12.5, 12.24; HRMS (ESI+) calcd for C_17_H_17_N_3_O_3_S + H_1_ 344.0991, found 344.0996.

##### 4.1.1.8. 1,3,5-Trimethyl-4-(2-oxo-2-(prop-2-yn-1-ylamino)acetyl)-N-(thiazol-2-yl)-1H-pyrrole-2-carboxamide (7h)

Yield 29%: ^1^H NMR (400 MHz, CDCl_3_) *δ* 8.74 (d, *J* = 4.0 Hz, 1 H), 8.45 (d, *J* = 8.0 Hz, 1 H), 7.47–7.44 (m, 1 H), 7.12 (s, 1 H), 4.21–4.19 (m, 2 H), 3.84 (s, 3 H), 2.67 (s, 3 H), 2.44 (s, 3 H), 2.32 (t, *J* = 4.0 Hz, 1 H); ^13^C NMR (101 MHz, CDCl_3_) *δ* 186.5, 162.4, 157.6, 151.8, 145.3, 141.0, 136.5, 135.0, 129.5, 120.9, 120.0, 116.1, 78.3, 72.4, 33.5, 29.4, 13.6, 12.8; HRMS (ESI+) calcd for C_16_H_16_N_4_O_3_S + H_1_ 345.0943, found 345.0949.

##### 4.1.1.9. 1,3,5-Trimethyl-N-(4-methylthiazol-2-yl)-4-(2-oxo-2-(prop-2-yn-1-ylamino)acetyl)-1H-pyrrole-2-carboxamide (7i)

Yield 37%: ^1^H NMR (400 MHz, CDCl_3_) *δ* 7.18–7.24 (m, 1 H), 6.54 (d, *J* = 1.0 Hz, 1 H), 5.30 (s, 1 H), 4.25–4.08 (m, 2 H), 3.75 (s, 3 H), 2.40 (s, 3 H), 2.36 (s, 3 H), 2.29 (t, *J* = 2.5 Hz, 1 H), 2.20 (d, *J* = 0.9 Hz, 3 H); ^13^C NMR (101 MHz, CDCl_3_) *δ* 186.3, 163.3, 159.0, 157.7, 147.0, 142.6, 125.8, 124.3, 118.2, 108.2, 78.5, 77.4, 77.2, 77.0, 76.7, 72.2, 32.7, 29.3, 29.1, 16.7, 12.5, 12.4; HRMS (ESI+) calcd for C_17_H_18_N_4_O_3_S + H_1_ 359.1100, found 359.1095.

##### 4.1.1.10. 1,3,5-Trimethyl-N-(3-methyl-1H-pyrazol-5-yl)-4-(2-oxo-2-(prop-2-yn-1-ylamino)acetyl)-1H-pyrrole-2-carboxamide (7j)

Yield 21%: ^1^H NMR (400 MHz, CDCl_3_) *δ* 6.97 (s, 1 H), 5.41 (s, 2 H), 5.32 (s, 1 H), 4.16 (dd, *J* = 5.6, 2.6 Hz, 2 H), 3.56 (s, 3 H), 2.41 (s, 3 H), 2.28 (t, *J* = 2.6 Hz, 1 H), 2.15 (s, 3 H), 2.10 (s, 3 H); ^13^C NMR (101 MHz, CDCl_3_) *δ*^13^C NMR (101 MHz, CDCl_3_) *δ* 186.0, 163.8, 154.0, 150.7, 142.8, 130.0, 125.0, 118.2, 90.2, 78.5, 72.2, 60.4, 32.9, 29.2, 21.1, 14.2, 12.7; HRMS (ESI+) calcd for C_17_H_19_N_5_O_3_ + H_1_ 342.1488, found 342.1498.

#### 4.1.2. General synthetic procedure for compounds 7k–m

To a solution of compound 6 (260 mg, 1 mmol, 1 eq.) in DCM (10 mL) were added oxalyl chloride (0.086 mL, 1.5 mmol, 1.5 eq.) and one drop of DMF at 0 °C. The reaction mixture was stirred at 0 °C for 1 h, and then was concentrated *in vacuo*. The residue was dried under vacuum for 30 min, and redissolved in dry DCM (10 mL). DIPEA (0.53 mL, 3 mmol, 3 eq.) and amine (1.5 mmol, 1.5 eq.) were added into the acid chloride solution at rt and the reaction mixture was stirred for 12 h. DCM (10 mL) was added to dilute the reaction mixture, and the organic layer was washed with saturated aq. NaHCO_3_ (5 mL), 1 N HCl (5 mL), and brine (5 mL). The combined organic layers were dried over anhydrous Na_2_SO_4_, filtered, and concentrated under reduced pressure. The crude residue was purified by Combi flash column chromatography on silica gel using a solvent gradient from 100% dichloromethane (DCM) to DCM/methanol (20 : 1) to afford compounds 7k–7m as pure products.

##### 4.1.2.1. 1,3,5-Trimethyl-N-(naphthalen-1-yl)-4-(2-oxo-2-(prop-2-yn-1-ylamino)acetyl)-1H-pyrrole-2-carboxamide (7k)

Yield 54%. ^1^H NMR (400 MHz, CDCl_3_) *δ* 8.10 (d, *J* = 7.5 Hz, 1 H), 8.01–7.79 (m, 3 H), 7.74 (d, *J* = 8.2 Hz, 1 H), 7.65–7.41 (m, 4 H), 7.08 (s, 1 H), 4.19 (dd, *J* = 5.6, 2.6 Hz, 2 H), 3.75 (s, 3 H), 2.53 (s, 3 H), 2.43 (s, 3 H), 2.30 (t, *J* = 2.6 Hz, 1 H); ^13^C NMR (101 MHz, CDCl_3_) *δ* 186.2, 163.7, 160.8, 142.1, 134.2, 132.1, 129.0, 126.8, 126.6, 126.1, 125.9, 125.8, 120.4, 120.1, 117.8, 78.5, 72.3, 32.5, 29.7, 29.3, 12.9, 12.3; HRMS (ESI+) calcd for C_23_H_21_N_3_O_3_ + H_1_ 388.1583, found 388.1580.

##### 4.1.2.2. N-(2-Fluoropyridin-4-yl)-1,3,5-trimethyl-4-(2-oxo-2-(prop-2-yn-1-ylamino)acetyl)-1H-pyrrole-2-carboxamide (7l)

Yield 36%. ^1^H NMR (400 MHz, CDCl_3_) *δ* 8.57–8.25 (m, 3 H), 7.89 (s, 1 H), 7.14 (s, 1 H), 4.18 (dd, *J* = 5.6, 2.6 Hz, 2 H), 3.76 (s, 3 H), 2.44 (s, 3 H), 2.41 (s, 3 H), 2.31 (t, *J* = 2.6 Hz, 1 H); ^13^C NMR (101 MHz, CDCl_3_) *δ* 186.2, 163.1, 160.0, 147.0, 1467.0, 142.8, 137.2, 137.0, 133.3, 133.3, 125.1, 118.3, 114.3, 78.4, 72.3, 32.7, 29.7, 12.6; HRMS (ESI+) calcd for C_18_H_17_FN_4_O_3_ + H_1_ 357.1285, found 357.1284.

##### 4.1.2.3. N-(Benzo[d]oxazol-2-yl)-1,3,5-trimethyl-4-(2-oxo-2-(prop-2-yn-1-ylamino)acetyl)-1H-pyrrole-2-carboxamide (7m)

Yield 62%. ^1^H NMR (400 MHz, CDCl_3_) *δ* 8.60 (s, 1 H), 7.79 (s, 1 H), 7.34 (dd, *J* = 7.9, 1.5 Hz, 1 H), 7.15–6.82 (m, 3 H), 4.20 (dd, *J* = 5.6, 2.6 Hz, 2 H), 3.67 (s, 3 H), 2.43 (s, 3 H), 2.36 (s, 3 H), 2.31 (t, *J* = 2.6 Hz, 1 H); ^13^C NMR (101 MHz, CDCl_3_) *δ* 186.0, 163.6, 161.0, 148.0, 142.5, 126.7, 125.7, 125.2, 124.3, 121.6, 120.6, 119.0, 118.0, 78.3, 72.4, 32.5, 29.4, 12.7, 12.2; HRMS (ESI+) calcd for C_20_H_18_N_4_O_4_ + H_1_ 379.1328, found 379.1324.

#### 4.1.3. *N*-(3,4-Difluorophenyl)-*N*,1,3,5-tetramethyl-1*H*-pyrrole-2-carboxamide (10)

Compound 9 was synthesized according to a reported procedure.^23^ To a solution of *N*-(3,4-difluorophenyl)-1,3,5-trimethyl-1*H*-pyrrole-2-carboxamide 9 (0.25 g, 0.95 mmol) and KOH (0.11 g, 1.9 mmol) in DMSO (10 mL) was added MeI (0.18 mL, 2.9 mmol) at ambient temperature. The mixture was stirred at ambient temperature overnight, and then poured into H_2_O (50 mL). The aqueous phase was extracted with EtOAc (3 × 15 mL), and the combined organic layers were dried over Na_2_SO_4_ and concentrated *in vacuo*. The resulting mixture was purified by flash chromatography (hexanes : EtOAc = 8 : 2 v/v) to give compound 10 (0.21 g, 0.75 mmol, 70%). ^1^H NMR (400 MHz, CDCl_3_) 7.02 (dd, *J* = 18.6, 8.9 Hz, 1H), 6.88 (m, 1H), 6.75 (m, 1H), 5.52 (s, 3H), 3.53 (s, 3H), 3.44 (s, 3H), 2.14 (s, 3H), 1.74 (s, 3H). ^13^C NMR (101 MHz, CDCl_3_) *d* 165.2, 150.8 (d, *J* = 13.7 Hz), 149.0 (d, *J* = 12.7 Hz), 148.4 (d, *J* = 13.7 Hz), 146.5 (d, *J* = 12.6 Hz), 141.3 (dd, *J* = 1.7, 3.6 Hz), 133.1, 123.7, 121.2 (dd, *J* = 5.97, 3.49 Hz), 116.8 (d, *J* = 18.6 Hz), 114.5 (d, *J* = 19.1 Hz), 37.7, 31.7, 12.3, 12.1. ^19^F NMR (377 MHz, CDCl_3_) *d* −135.9 (d, *J* = 21.8 Hz), −141.2 (d, *J* = 21.8 Hz). HRMS (ESI): *m*/*z* [M + H]^+^ calcd for C_15_H_16_F_2_N_2_O: 279.1264, found: 279.1300.

#### 4.1.4. Ethyl 2-(5-((3,4-difluorophenyl)(methyl)carbamoyl)-1,2,4-trimethyl-1*H*-pyrrol-3-yl)-2-oxoacetate (11)

A solution of *N*-(3,4-difluorophenyl)-*N*,1,3,5-tetramethyl-1*H*-pyrrole-2-carboxamide 10 (0.17 g, 0.61 mmol) and aluminum trichloride (0.25 g, 1.9 mmol, 3) in DCM (6 mL) was cooled to 0 °C and ClCOCO_2_Et (0.21 mL, 1.9 mmol) was added in a dropwise manner over 30 min. The solution was allowed to slowly come to ambient temperature and was stirred under nitrogen for 24 hours after which it was poured over aqueous NH_4_Cl (20 mL). The aqueous phase was extracted with DCM (3 × 10 mL). The combined organic phase was dried over anhydrous Na_2_SO_4_, filtered and concentrated *in vacuo*. The crude product was purified by flash column chromatography on silica gel using hexane/ethyl acetate (8 : 2, v/v) as the eluent to afford compound 11 (0.19 g, 0.50 mmol, 62%) as a pure product. ^1^H NMR (400 MHz, CDCl_3_) *d* 7.08 (dd, *J* = 18.2, 8.9 Hz, 1H), 6.95 (m, 1H), 6.97 (m, 1H), 4.31 (q, *J* = 7.1 Hz, 2H), 3.58 (s, 3H), 3.45 (s, 3H), 2.41 (s, 3H), 1.91 (s, 3H), 1.34 (t, *J* = 7.1 Hz, 3H). ^13^C NMR (101 MHz, CDCl_3_) *d* 183.0, 165.8, 163.8, 158.3 (d, *J* = 23.4 Hz), 151.0 (d, *J* = 13.7 Hz), 149.8 (d, *J* = 12.6 Hz), 148.5 (d, *J* = 13.7 Hz), 147.4 (d, *J* = 12.5 Hz), 141.5, 139.8 (dd, *J* = 7.2, 3.4 Hz), 125.9, 121.8 (dd, *J* = 6.3, 3.5 Hz), 121.3, 117.5 (d, *J* = 18.3 Hz), 116.8, 115.0 (d, *J* = 18.9 Hz), 61.8, 37.9, 31.9, 13.9, 11.7, 11.3. ^19^F NMR (377 MHz, CDCl_3_) *d* −134.8 (d, *J* = 21.5 Hz), −138.9 (d, *J* = 21.5 Hz). HRMS (ESI): *m*/*z* [m + 1]^+^ calcd for C_19_H_20_F_2_N_2_O_4_: 379.1391, found: 379.1460.

#### 4.1.5. *N*-(3,4-Difluorophenyl)-*N*,1,3,5-tetramethyl-4-(2-oxo-2-(prop-2-yn-1-ylamino)acetyl)-1*H*-pyrrole-2-carboxamide (13)

A solution of ethyl 2-(5-((3,4-difluorophenyl)(methyl)carbamoyl)-1,2,4-trimethyl-1*H*-pyrrol-3-yl)-2-oxoacetate 11 (0.15 g, 0.40 mmol) in MeOH (4 mL) and THF (2 mL) was cooled to 0 °C and 5% aqueous NaOH (4 mL) was added slowly. The solution was stirred at 0 °C for 20 min after which time the solution was acidified to pH 4–5 by the slow addition of 1 N HCl. The solution was partitioned between water (20 mL) and EtOAc (20 mL). The aqueous phase was extracted with EtOAc (3 × 10 mL). The combined organic phase was dried over anhydrous Na_2_SO_4_, filtered and concentrated *in vacuo*. The residue 12 was then taken up in DMF (4 mL) and CDI (0.32 g, 1.97) was added. After stirring the solution for 30 min under nitrogen at ambient temperature, propargyl amine (0.12 mL, 1.87 mmol) was added. The reaction was stirred for 24 hours after which time the solution was poured over water (25 mL) and extracted with EtOAc (3 × 15 mL). The combined organic phase was dried over anhydrous Na_2_SO_4_, filtered and concentrated *in vacuo*. The residue was purified *via* flash chromatography using hexanes : EtOAc (6.5 : 3.5 v/v) as the eluent to give 13 (0.10 g, 0.26 mmol, 43%) as a white solid. ^1^H NMR (400 MHz, DMSO-d_6_) *d* 9.04 (t, *J* = 5.5 Hz, 1H), 7.47 (m, 1H), 7.02 (m, 1H), 3.96 (m, 2H), 3.54 (s, 3H), 3.37 (s, 3H), 3.15 (t, *J* = 2.3 Hz), 2.33 (s, 3H), 1.63 (s, 3H). ^13^C NMR (101 MHz, DMSO-d_6_) *d* 187.5, 167.6, 163.3, 150.4 (d, *J* = 13.4 Hz), 149.2 (d, *J* = 12.5), 148.0 (d, *J* = 13.4), 146.8 (d, *J* = 12.5), 140.7, 126.6, 123.4 (dd, *J* = 6.3, 3.1 Hz), 120.0, 117.7 (d, *J* = 18.0 Hz), 115.9, 115.8, (d, *J* = 18.7 Hz), 80.5, 73.7, 37.7, 32.3, 27.9, 11.7. ^19^F NMR (377 MHz, DMSO-d_6_) *d* −136.9 (d, *J* = 24.4 Hz), −148.9 (d, *J* = 24.5 Hz). HRMS (ESI): *m*/*z* [m + 1]^+^ calcd for C_20_H_20_F_2_N_3_O_3_: 388.1473, found: 388.1465.

#### 4.1.6. 2-(5-((3,4-Difluorophenyl)carbamoyl)-1,2,4-trimethyl-1*H*-pyrrol-3-yl)-2-oxoacetic acid (15)

To a solution of 9 (860 mg, 3.26 mmol) in DCM (30 mL) were added ethyl oxalylchloride (980 μL, 8.80 mmol) and AlCl_3_ (1.08 g, 8.15 mmol) at 0 °C. The mixture was stirred at room temperature for 16 h and poured into crushed ice. The mixture was extracted with DCM and the combined organic layers were filtered on Celite. The filtrate was concentrated and the resulting residue was used in the next step without further purification. To a solution of crude 14 in EtOH was added NaOH 10% (25 mL). The mixture was stirred for 1 h at room temperature. EtOH was evaporated under vacuum, and the mixture was extracted with EtOAc (3 × 10 mL). The aqueous layer was acidified with 1 M HCl. The mixture was extracted with EtOAc (3 × 10 mL). The combined organic layers were dried over MgSO_4_ and concentrated *in vacuo*. The resulting solid was washed with Et_2_O to afford 15 in 59% yield (646 mg) over two steps. ^1^H NMR (400 MHz, DMSO-*d*_6_) *δ* 14.13 (s, 1H), 10.46 (s, 1H), 8.04–7.71 (m, 1H), 7.59–7.28 (m, 2H), 3.60 (s, 3H), 2.46 (s, 3H), 2.26 (s, 3H). ^13^C NMR (101 MHz, DMSO-*d*_6_) *δ* 185.5, 168.5, 160.4, 149.4 (dd, *J* = 244.3 Hz, *J* = 13.2 Hz), 146.0 (dd, *J* = 242.9 Hz, *J* = 12.6 Hz), 141.5, 136.3 (dd, *J* = 8.9 Hz, *J* = 2.8 Hz), 127.5, 122.3, 117.9 (d, *J* = 17.8 Hz), 116.5 (dd, *J* = 5.8 Hz, 3.4 Hz), 115.4, 109.1 (d, *J* = 21.7 Hz), 32.4, 11.6, 11.5. HRMS (ESI) *m*/*z* calcd for C_16_H_15_F_2_N_2_O_4_ [M + H]^+^: 337.1000; found 337.0990.

#### 4.1.7. General synthetic procedure for compounds 16a–j

To a solution of compound 15 (1.0 eq.) and HATU (3.0 eq.) in DMF were added DIPEA (6.0 eq.) and substituted amine R_1_a–j (4.0 eq.). The resulting mixture was stirred at 60–80 °C overnight and was quenched with cold H_2_O (20 mL). The water layer was then extracted with EtOAc (2 × 50 mL). The organic layers were finally combined, washed with water and brine, dried over anhydrous Na_2_SO_4_, and concentrated under reduced pressure. The residue was purified by flash chromatography (hexane/ethyl acetate 10 : 1 to 0 : 100) to afford products 16a–j (60–80%).

##### 4.1.7.1. N-(3,4-Difluorophenyl)-4-(2-((3-hydroxyprop-1-yn-1-yl)amino)-2-oxoacetyl)-1,3,5-trimethyl-1H-pyrrole-2-carboxamide (16a)

Yield 61%. ^1^H NMR (400 MHz, DMSO-*d*_6_) *δ* 10.43 (s, 1H), 9.14 (t, *J* = 6.4 Hz, 1H), 7.90–7.85 (m, 1H), 7.45–7.41 (m, 2H), 5.19 (t, *J* = 5.8 Hz, 1H), 4.08–4.03 (m, 4H), 3.59 (s, 3H), 2.40 (s, 3H), 2.23 (s, 3H). ^13^C NMR (101 MHz, DMSO-*d*_6_) *δ* 187.9, 167.5, 160.5, 141.2, 136.4, 127.3, 122.8, 117.9, 116.5, 109.1, 82.8, 80.5, 49.4, 32.4, 28.3, 11.9. ^19^F NMR (377 MHz, DMSO-*d*_6_) *δ* −137.1 to −137.3 (m, 1F), −144.2 to −144.3 (m, 1F). HRMS (ESI) *m*/*z* calcd for C_20_H_20_F_2_N_3_O_4_ [M + H]^+^: 404.1422; found 404.1417.

##### 4.1.7.2. N-(3,4-Difluorophenyl)-4-(2-((3-hydroxypropyl)amino)-2-oxoacetyl)-1,3,5-trimethyl-1H-pyrrole-2-carboxamide (16b)

Yield 73%. ^1^H NMR (400 MHz, DMSO-*d*_6_) *δ* 10.41 (s, 1H), 8.65 (t, *J* = 5.6 Hz, 1H), 7.90–7.84 (m, 1H), 7.45–7.41 (m, 2H), 4.51 (t, *J* = 5.2 Hz, 1H), 3.59 (s, 3H), 3.48–3.43 (m, 2H), 3.26–3.21 (m, 2H), 2.39 (s, 3H), 2.23 (s, 3H), 1.67–1.63 (m, 2H). ^13^C NMR (101 MHz, DMSO-*d*_6_) *δ* 188.5, 167.8, 160.6, 150.6, 148.2, 147.2, 144.8, 141.0, 136.4, 127.2, 122.8, 117.9, 116.8, 116.4, 109.1, 58.9, 36.2, 32.3, 11.8. ^19^F NMR (377 MHz, DMSO-*d*_6_) *δ* −137.2 to −137.3 (m, 1 F), −144.3 to −144.4 (m, 1F). HRMS (ESI) *m*/*z* calcd for C_19_H_22_F_2_N_3_O_4_ [M + H]^+^: 394.1578; found 394.1581.

##### 4.1.7.3. N-(3,4-Difluorophenyl)-4-(2-((4-hydroxybutyl)amino)-2-oxoacetyl)-1,3,5-trimethyl-1H-pyrrole-2-carboxamide (16c)

Yield 52%. ^1^H NMR (400 MHz, DMSO-*d*_6_) *δ* 10.34 (s, 1H), 8.60 (t, *J* = 5.7 Hz, 1H), 7.82–7.77 (m, 1H), 7.38–7.34 (m, 2H), 4.37 (t, *J* = 5.1 Hz, 1H), 3.51 (s, 3H), 3.34–3.33 (m, 2H), 3.11–3.09 (m, 2H), 2.32 (s, 3H), 2.15 (s, 3H), 1.47–1.36 (m, 4H). ^13^C NMR (101 MHz, DMSO-*d*_6_) *δ* 188.6, 167.8, 160.6, 150.6, 148.2, 147.2, 144.7, 141.0, 136.4, 127.2, 122.7, 118.0, 117.9, 116.4, 109.1, 60.8, 32.3, 30.4, 25.8, 11.8. ^19^F NMR (377 MHz, DMSO-*d*_6_) *δ* −137.2 to −137.3 (m, 1F), −144.3 to −144.4 (m, 1F). HRMS (ESI) *m*/*z* calcd for C_20_H_24_F_2_N_3_O_4_ [M + H]^+^: 408.1735; found 408.1735.

#### 4.1.8. 4-(2-(((1*H*-1,2,3-Triazol-5-yl)methyl)amino)-2-oxoacetyl)-*N*-(3,4-difluorophenyl)-1,3,5-trimethyl-1*H*-pyrrole-2-carboxamide (16d)

##### 4.1.8.1. Procedure for the synthesis of compound 16d

The synthesis of compound 16d was performed by following the procedure reported in ref. 34. ^1^H NMR (400 MHz, MeOH-*d*_4_) *δ* 7.82–7.77 (m, 2H), 7.36–7.34 (m, 1H), 7.29–7.22 (m, 1H), 4.64 (s, 2H), 3.65 (s, 3H), 2.41 (s, 3H), 2.29 (s, 3H). ^13^C NMR (101 MHz, MeOH-*d*_4_) *δ* 187.2, 168.1, 161.2, 151.0, 148.6, 148.0, 145.5, 142.2, 141.9, 135.2, 126.8, 123.5, 116.9, 116.6, 160.0, 109.3, 33.5, 31.1, 10.5. ^19^F NMR (377 MHz, MeOH-*d*_4_) *δ* −138.9 to −139.0 (m, 1 F), −145.5 to −145.6 (m, 1 F). HRMS (ESI): *m*/*z* [M + 1]^+^ calcd for C_19_H_19_F_2_N_6_O_3_: 417.1487, found 417.1475.

#### 4.1.9. 4-(2-(Bicyclo[1.1.1]pentan-2-ylamino)-2-oxoacetyl)-*N*-(3,4-difluorophenyl)-1,3,5-trimethyl-1*H*-pyrrole-2-carboxamide (16e)

Yield 31%. ^1^H NMR (400 MHz, chloroform-*d*) *δ* 7.72 (ddd, *J* = 11.0, 6.7, 2.0 Hz, 1H), 7.52 (s, 1H), 7.18 (d, *J* = 2.1 Hz, 1H), 7.14–7.10 (m, 2H), 3.70 (s, 3H), 2.52 (s, 1H), 2.38 (s, 3H), 2.36 (s, 3H), 2.18 (s, 6H); ^19^F NMR (377 MHz, chloroform-*d*) *δ* −135.29 (d, *J* = 21.8 Hz), −142.21 (d, *J* = 21.7 Hz); ^13^C NMR (101 MHz, CDCl_3_) *δ* 187.2, 163.8, 160.1, 151.5, 151.4, 149.1, 148.4, 145.8, 141.9, 134.2, 134.2, 125.8, 123.4, 117.9, 117.5, 117.3, 115.3, 115.3, 115.2, 115.2, 109.7, 109.5, 52.7, 48.4, 32.3, 25.1, 12.5, 12.1; HRMS (ESI): *m*/*z* [M + H]^+^ calcd for C_21_H_22_F_2_N_3_O_3_: 402.1629, found: 402.1627.

#### 4.1.10. 4-(2-(Cuban-1-ylamino)-2-oxoacetyl)-*N*-(3,4-difluorophenyl)-1,3,5-trimethyl-1*H*-pyrrole-2-carboxamide (16f)

Yield 66%. ^1^H NMR (400 MHz, CD_3_OD) *δ* 7.80–7.76 (m, 1H), 7.35–7.33 (m, 1H), 7.28–7.21 (m, 1H), 4.27–4.24 (m, 3H), 4.01–3.99 (m, 4H), 3.66 (s, 3H), 2.47 (s, 3H), 2.34 (s, 3H); ^13^C NMR (101 MHz, CD_3_OD) *δ* 188.7, 169.0, 162.6, 143.3, 128.2, 124.8, 118.4, 118.2, 118.0, 117.4, 110.8, 110.5, 67.6, 53.5, 44.3, 32.5, 12.0, 11.9; ^19^F NMR (377 MHz, CD_3_OD) *δ* −138.9 to −139.0 (m, 1 F), −145.5 to −145.6 (m, 1F); HRMS (ESI): *m*/*z* [M + H]^+^ calcd for C_24_H_22_F_2_N_3_O_3_: 438.1629, found: 438.1611.

#### 4.1.11. 4-(2-(((3*S*,5*S*,7*S*)-Adamantan-1-yl)amino)-2-oxoacetyl)-*N*-(3,4-difluorophenyl)-1,3,5-trimethyl-1*H*-pyrrole-2-carboxamide (16g)

Yield 48%. ^1^H NMR (400 MHz, CDCl_3_) *δ* 7.78–7.67 (m, 1H), 7.54 (s, 1H), 7.17–7.08 (m, 2H), 6.49 (s, 1H), 3.69 (s, 3H), 2.38 (d, *J* = 5.9 Hz, 6H), 2.18–2.11 (m, 3H), 2.09 (d, *J* = 2.9 Hz, 6H), 1.79–1.67 (m, 6H); ^19^F NMR (377 MHz, CDCl_3_) *δ* −135.32 (d, *J* = 21.8 Hz), −142.28 (d, *J* = 21.8 Hz); ^13^C NMR (101 MHz, CDCl_3_) *δ* 188.2, 163.3, 160.1, 151.5, 151.4, 149.1, 148.9, 148.4, 148.2, 145.9, 145.8, 141.8, 134.3, 134.3, 125.7, 123.4, 117.9, 117.5, 117.3, 115.3, 115.2, 115.2, 115.2, 109.7, 109.5, 52.2, 41.1, 36.2, 32.3, 29.4, 12.6, 12.1; HRMS (ESI): *m*/*z* [M + H]^+^ calcd for C_26_H_30_F_2_N_3_O_3_: 470.2255 found: 470.2259.

#### 4.1.12. 4-(2-((1-((3*R*,5*R*,7*R*)-Adamantan-1-yl)ethyl)amino)-2-oxoacetyl)-*N*-(3,4-difluorophenyl)-1,3,5-trimethyl-1*H*-pyrrole-2-carboxamide (16h)

Yield 60%. ^1^H NMR (400 MHz, DMSO-*d*_6_) *δ* 10.40 (s, 1H), 8.35 (d, *J* = 9.4 Hz, 1H), 7.99–7.79 (m, 1H), 7.43 (dd, *J* = 6.8, 1.9 Hz, 2H), 3.59 (s, 3H), 3.34 (s, 3H), 2.41 (s, 3H), 2.24 (s, 3H), 1.95 (s, 3H), 1.74–1.57 (m, 5H), 1.56–1.49 (m, 6H), 1.01 (d, *J* = 6.9 Hz, 3H); ^19^F NMR (377 MHz, DMSO-*d*_6_) *δ* −137.24 (d, *J* = 23.2 Hz), −144.35 (d, *J* = 23.0 Hz); ^13^C NMR (101 MHz, DMSO-*d*_6_) *δ* 188.7, 167.9, 160.6, 150.6, 150.5, 148.2, 148.1, 147.2, 147.0, 144.8, 144.7, 140.9, 136.4, 127.1, 122.8, 118.0, 117.9, 116.8, 116.5, 109.2, 109.0, 79.8, 79.4, 79.1, 52.7, 38.4, 37.1, 36.2, 32.3, 28.2, 14.2, 11.9, 11.8; HRMS (ESI): *m*/*z* [M + H]^+^ calcd for C_28_H_34_F_2_N_3_O_3_: 498.2568 found: 498.2570.

#### 4.1.13. *N*-(3,4-Difluorophenyl)-1,3,5-trimethyl-4-(2-oxo-2-((3-(3a,5,5-trimethylhexahydro-4,6-methanobenzo[*d*] [1,3,2] dioxaborol-2-yl) propyl) amino)acetyl)-1*H*-pyrrole-2-carboxamide (16i)

Yield 45%. ^1^H NMR (400 MHz, acetone-*d*_6_) *δ* 9.52 (s, 1H), 8.00–7.95 (m, 1H), 7.81–7.79 (m, 1H), 7.53–7.49 (m, 1H), 7.34–7.27 (m, 1H), 4.29 (d, *J* = 40 Hz, 1H), 3.66 (s, 3H), 4.29 (dd, *J* = 8.0 Hz, *J* = 12 Hz, 2H), 2.39–2.27 (m, 4H), 2.22 (s, 3H), 2.21–2.18 (m, 1H), 1.97–1.99 (m, 1H), 1.81–1.76 (m, 1H), 1.88–1.86 (m, 1H), 1.72–1.69 (m, 2H), 1.35 (s, 3H), 1.27 (s, 3H), 1.11 (t, *J* = 8.0 Hz, 1H), 0.86–0.82 (m, 5H). ^13^C NMR (101 MHz, acetone-*d*_6_) *δ* 187.9, 166.7, 160.4, 150.9, 148.4, 147.5, 145.0, 140.8, 136.1, 126.5, 123.0, 117.2, 115.8, 109.0, 108.7, 85.2, 77.4, 51.3, 41.0, 39.5, 37.9, 35.3, 31.5, 29.5, 28.5, 26.4, 23.6 11.2, 11.0. ^19^F NMR (377 MHz, acetone-*d*_6_) *δ* −138.2 to −138.4 (m, 1 F), −145.5 to −145.6 (m, 1F). HRMS (ESI) *m*/*z* calcd for C_29_H_37_BF_2_N_3_O_5_ [M + H]^+^: 556.2794; found 556.2805.

#### 4.1.14. (3-(2-(5-((3,4-Difluorophenyl)carbamoyl)-1,2,4-trimethyl-1*H*-pyrrol-3-yl)-2-oxoacetamido)propyl)boronic acid (17)

Compounds 16i and 17 were synthesized by adapting the procedures described in the patent literature.^35^ To a solution of compound 16i (30 mg, 0.054 mmol) and phenyl boronic acid in Et_2_O (4 mL) were added dropwise 1 N HCl (0.5 mL) and H_2_O (2 mL). The reaction mixture was then stirred at 50 °C for 16 h and the solvent was removed *in vacuo*. The residue was purified by flash chromatography (DCM : MeOH = 100 : 1 v/v to 5 : 1) to give compound 17 as a yellowish powder (70%, 16 mg). ^1^H NMR (400 MHz, acetone-*d*_6_) *δ* 9.48 (s, 1H), 8.01–7.96 (m, 1H), 7.80 (t, *J* = 5.3 Hz, 1H), 7.53–7.49 (m, 1H), 7.36–7.29 (m, 1H), 6.77 (s, 2H), 3.68 (s, 3H), 3.34–3.29 (m, 2H), 2.89 (s, 3H), 2.42 (s, 3H), 1.75–1.67 (m, 2H), 0.79 (t, *J* = 7.8 Hz, 2H). ^13^C NMR (101 MHz, acetone-*d*_6_) *δ* 189.7, 168.3, 162.2, 152.7, 150.3, 149.2, 146.8, 142.6, 128.3, 124.6, 119.0, 117.5, 110.5, 51.3, 50.7, 42.9, 33.2, 26.0, 12.8. ^19^F NMR (377 MHz, acetone-*d*_6_) *δ* −138.5 to −138.6 (m, 1 F), −145.7 to −145.8 (m, 1F). HRMS (ESI) *m*/*z* calcd for C_19_H_23_BF_2_N_3_O_5_ [M + H]^+^: 422.1699; found 422.1698.

#### 4.1.15. *N*-(3,4-Difluorophenyl)-1,3,5-trimethyl-4-(2-oxo-2-(prop-1-yn-1-ylamino)acetyl)-1*H*-pyrrole-2-carboxamide (18)

To a solution of GLP-26 (40 mg, 0.11 mmol, 1.0 eq.) in EtOH (20 mL) was added 20% NaOH (5.0 mL). The resulting mixture was stirred at 100 °C for 24 h and the solvent was removed under reduced pressure. The mixture was diluted with 10 mL of cold H_2_O and extracted with ethyl acetate (3 × 30 mL). The organic layers were combined, washed with water and brine, dried over anhydrous Na_2_SO_4_, and concentrated under reduced pressure. The residue was purified by flash chromatography (hexane/EtOAc, from 10 : 1 to 1 : 10) to afford compound 18 (31 mg, 83%). ^1^H NMR (400 MHz, acetone-*d*_6_) *δ* 9.46 (s, 1H), 8.01–7.96 (m, 1H), 7.55–7.50 (m, 1H), 7.35–7.28 (m, 1H), 7.07 (s, 1H), 3.70 (s, 3H), 2.44 (s, 3H), 2.29 (s, 3H), 2.20 (s, 3H); ^13^C NMR (101 MHz, acetone-*d*_6_) *δ* 177.4, 160.4, 158.5, 152.2, 150.9, 148.5, 147.4, 145.0, 139.3, 136.2, 124.5, 122.5, 120.0, 117.2, 115.7, 108.8, 31.5, 11.3, 10.1; ^19^F NMR (377 MHz, acetone-*d*_6_) *δ* −138.5 to −138.6 (m, 1 F), −145.8 to −145.9 (m, 1F). HRMS (ESI) *m*/*z* calcd for C_19_H_18_F_2_N_3_O_3_ [M + H]^+^: 374.1316; found 374.1317.

#### 4.1.16. 2-(5-((Benzyloxy)carbonyl)-1,2,4-trimethyl-1*H*-pyrrol-3-yl)acetic acid (20)

Compound 19 was prepared following the method reported in ref. 33. Compound 19 (210 mg, 0.67 mmol) was dissolved in DMSO (4.0 mL), and methyl iodide (190 mg, 1.34 mmol) was added, followed by the addition of KOH (78 mg, 1.4 mmol). The reaction mixture was stirred at room temperature overnight. Upon completion of the reaction, the mixture was diluted with ethyl acetate (15 mL) and washed with water (3 × 5 mL). The organic layer was dried over Na_2_SO_4_, filtered, and concentrated under reduced pressure. The crude product was purified by flash chromatography on silica gel using a gradient of hexane/ethyl acetate (20 : 1 to 5 : 1) to afford the corresponding new methylated derivative 20 (145 mg, 66%). ^1^H NMR (400 MHz, CDCl_3_) *δ* 7.51–7.39 (m, 2H), 7.39–7.29 (m, 3H), 5.29 (s, 2H), 4.11 (q, *J* = 7.1 Hz, 2H), 3.78 (s, 3H), 3.38 (s, 2H), 2.27 (s, 3H), 2.18 (s, 3H), 1.24 (t, *J* = 7.1 Hz, 3H); ^13^C NMR (101 MHz, CDCl_3_) *δ* 171.9, 162.0, 136.8, 134.6, 128.5, 128.4, 128.0, 127.9, 118.5, 113.4, 65.3, 60.7, 33.1, 30.4, 14.2, 11.9, 10.6. HRMS (ESI): *m*/*z* [M + H]^+^ calcd for C_19_H_24_NO_4_: 330.1705, found: 330.1709.

#### 4.1.17. Benzyl 1,3,5-trimethyl-4-(2-oxo-2-(prop-2-yn-1-ylamino)ethyl)-1*H*-pyrrole-2-carboxylate (22)

A solution of compound 20 (67 mg, 0.2 mmol) in a 1 : 1 mixture of methanol and tetrahydrofuran (1 mL) was treated with 1 N NaOH (0.5 mL, 0.5 mmol). The reaction mixture was stirred at room temperature for 2 hours. Upon completion of the reaction, the pH was adjusted to 1–2 using 1 N HCl, and the mixture was extracted with ethyl acetate (3 × 5 mL). The combined organic layers were dried over anhydrous Na_2_SO_4_, filtered, and concentrated under reduced pressure to afford compound 21 as a crude product (52 mg, 86% yield). Subsequently, a mixture of compound 21 (50 mg, 0.17 mmol), the corresponding amine (17 mg, 0.31 mmol), and HATU (80 mg, 0.21 mmol) was dissolved in DMF (0.8 mL). To this solution, DIPEA (65 mg, 0.5 mmol) was added, and the reaction mixture was stirred at room temperature for 15 hours. After completion of the reaction, DMF was removed under reduced pressure. The residue was purified by thin-layer chromatography (TLC) using a hexane/ethyl acetate (2 : 1) solvent system to afford the new amide 22 (34 mg, 60% yield). ^1^H NMR (400 MHz, CDCl_3_) *δ* 7.50–7.41 (m, 2H), 7.40–7.33 (m, 3H), 5.69 (s, 1H), 5.31 (s, 2H), 3.99 (dd, *J* = 5.5, 2.5 Hz, 2H), 3.81 (s, 3H), 3.37 (s, 2H), 2.22 (s, 3H), 2.16 (s, 4H); ^13^C NMR (101 MHz, CDCl_3_) *δ* 170.9, 161.8, 136.5, 134.7, 128.6, 128.1, 128.1, 128.1, 119.2, 113.2, 79.6, 71.3, 65.5, 33.2, 32.2, 29.1, 11.8, 10.5. HRMS (ESI): *m*/*z* [M + H]^+^ calcd for C_20_H_23_N_2_O_3_ 339.1709 found: 339.1711.

#### 4.1.18. *N*-(3,4-Difluorophenyl)-1,3,5-trimethyl-4-(2-oxo-2-(prop-2-yn-1-ylamino)ethyl)-1*H*-pyrrole-2-carboxamide (23)

To a solution of compound 22 (10 mg, 0.03 mmol) and difluoroaniline (6.0 mg 0.046 mmol) in THF (0.4 mL) was added 1 M LiHMDS in THF (0.2 mL, 0.2 mmol) dropwise at room temperature. The reaction mixture warmed up to 40 °C and stirred for 1 h. The reaction was quenched with ice-water (2 mL) and extracted with AcOEt (3 mL × 3). The combined organic layers were dried over Na_2_SO_4_, filtered and concentrated under vacuum. The residue was purified by preparative TLC (hexanes/EA = 1 : 1) to give compound 23 (7.3 mg 70%). ^1^H NMR (400 MHz, CDCl_3_) *δ* 7.75–7.63 (m, 1H), 7.36 (s, 1H), 7.17–7.08 (m, 2H), 5.76 (s, 1H), 4.01 (dd, *J* = 5.6, 2.6 Hz, 2H), 3.75 (s, 3H), 3.39 (s, 2H), 2.25 (s, 3H), 2.18 (d, *J* = 5.8 Hz, 4H); ^19^F NMR (377 MHz, CDCl_3_) *δ* −135.49 (d, *J* = 21.7 Hz), −142.75 (d, *J* = 21.7 Hz); ^13^C NMR (101 MHz, CDCl_3_) *δ* 170.8, 160.5, 151.5, 151.4, 149.1, 148.9, 148.2, 148.1, 145.8, 145.6, 134.5, 134.5, 134.5, 134.4, 133.4, 123.7, 120.6, 117.4, 117.2, 115.3, 115.3, 115.2, 115.2, 112.5, 109.7, 109.5, 79.5, 71.4, 32.6, 32.3, 29.1, 11.3, 10.3; HRMS (ESI): *m*/*z* [M + H]^+^ calcd for C_19_H_20_F_2_N_3_O_2_ 360.1524 found: 360.1520.

### 4.2. Anti-HBV activity in HepAD38

The HBV 7-day assay was performed in HepAD38 wild-type (HepAD38) cells as previously described.^25,27^ Briefly, HepAD38 cells were seeded in 96-well plates and incubated for two days at 37 °C under a humidified 5% CO_2_ atmosphere. On day two, the medium was removed, and cells were washed with 1× phosphate buffered saline (PBS). Forty µM stock solutions of the compounds were prepared for the assay. The desired aliquot of the solution was diluted in medium without tetracycline and added in duplicate at various concentrations to the wells. On day seven, total DNA was extracted using the DNeasy 96 Tissue kit (Qiagen), and HBV DNA was amplified by RT-PCR.^25,27^ Antiviral activity was measured by determining the average threshold cycle for HBV amplification of the compounds (alone or in combination), which was subtracted from the average cycle of the untreated-tetracycline control (ΔCT). Drugs were first tested individually for effective concentration, which inhibited 50% and 90% of HBV DNA replication (EC_50_ and EC_90_), using the CalcuSyn software program (Biosoft, Cambridge, UK).

### 4.3. Cytotoxicity assays

Cytotoxicity was determined by using the CellTiter 96 non-radioactive cell proliferation colorimetric assay (Promega) in human peripheral blood mononuclear (PBM) cells and in human T lymphoblast (CEM), African green monkey kidney (Vero), and human hepatocellular carcinoma (HepG2) cells. Toxicity levels were measured as the concentration of the test compound that inhibited cell growth by 50% (CC_50_).

## Author contributions

Conceptualization: FA. Funding acquisition: FA and RFS. Resources: RFS. Data analysis: ZC, SZ, PL, JW, LZ, CHF, HBB, MS, KV, DRM, JDB, LB, and FA. Investigation: ZC, SZ, PL, JW, LZ, CHF, HBB, MS, KV, DRM, JDB, LB, DP, and FA. Computational data: DP. Supervision: FA, RFS, and LB. Writing of draft: FA and ZC. Writing – editing and review: FA and RFS.

## Conflicts of interest

Drs Schinazi, Amblard, and Bassit, along with Emory University, are entitled to equity and royalties related to products licensed to Aligos Therapeutics, Inc., being further evaluated in the research described in this paper. The terms of this arrangement have been reviewed and approved by Emory University in accordance with its conflict-of-interest policies.

## Supplementary Material

OB-024-D6OB00943C-s001

## Data Availability

The data supporting this article have been included as part of the supplementary information (SI). Supplementary information: NMR spectra. See DOI: https://doi.org/10.1039/d6ob00943c.
