# Supplementary material for: 4-Diketopyrrole-2-carboxamide derivatives affecting HBV capsid assembly: structure–activity relationship around the GLP-26 scaffold
Source: Org Biomol Chem. 2026 Oct 5;24(40):8323–38. doi: 10.1039/d6ob00943c (PMC13650461; doi:10.1039/d6ob00943c)
Supplement: Supplementary file 1 [file OB-024-D6OB00943C-s001.pdf]

Supplementary Information (SI) for Organic & Biomolecular Chemistry.
This journal is © The Royal Society of Chemistry

Supporting Information

4-Diketopyrrole-2-carboxamide Derivatives Affecting HBV Capsid Assembly: Structure–Activity Relationship around the GLP-26 Scaffold

Zhe Chen, Shaoman Zhou, Peng Liu, John Wiseman, Longhu Zhou, Chia-Han (Vanessa) Fan, Heather B. Bowers, Mohammad Salman, Kiran Verma, Danielle R. Monroe, Jessica Downs-Bowen, Dharmeshkumar Patel, Leda Bassit, Raymond F. Schinazi, Franck Amblard**

[1]Center for ViroScience and Cure, Laboratory of Biochemical Pharmacology, Department of Pediatrics, Emory University School of Medicine, and Children's Healthcare of Atlanta, Atlanta, GA 30322, USA

*Correspondence: rschina@emory.edu (RFS); famblar@emory.edu (FA)

Table of Content:.

NMR spectra for compounds 7a-m, **13**, **16a-h**, 17, **18** and **23** 2-60

NMR of compound 7a

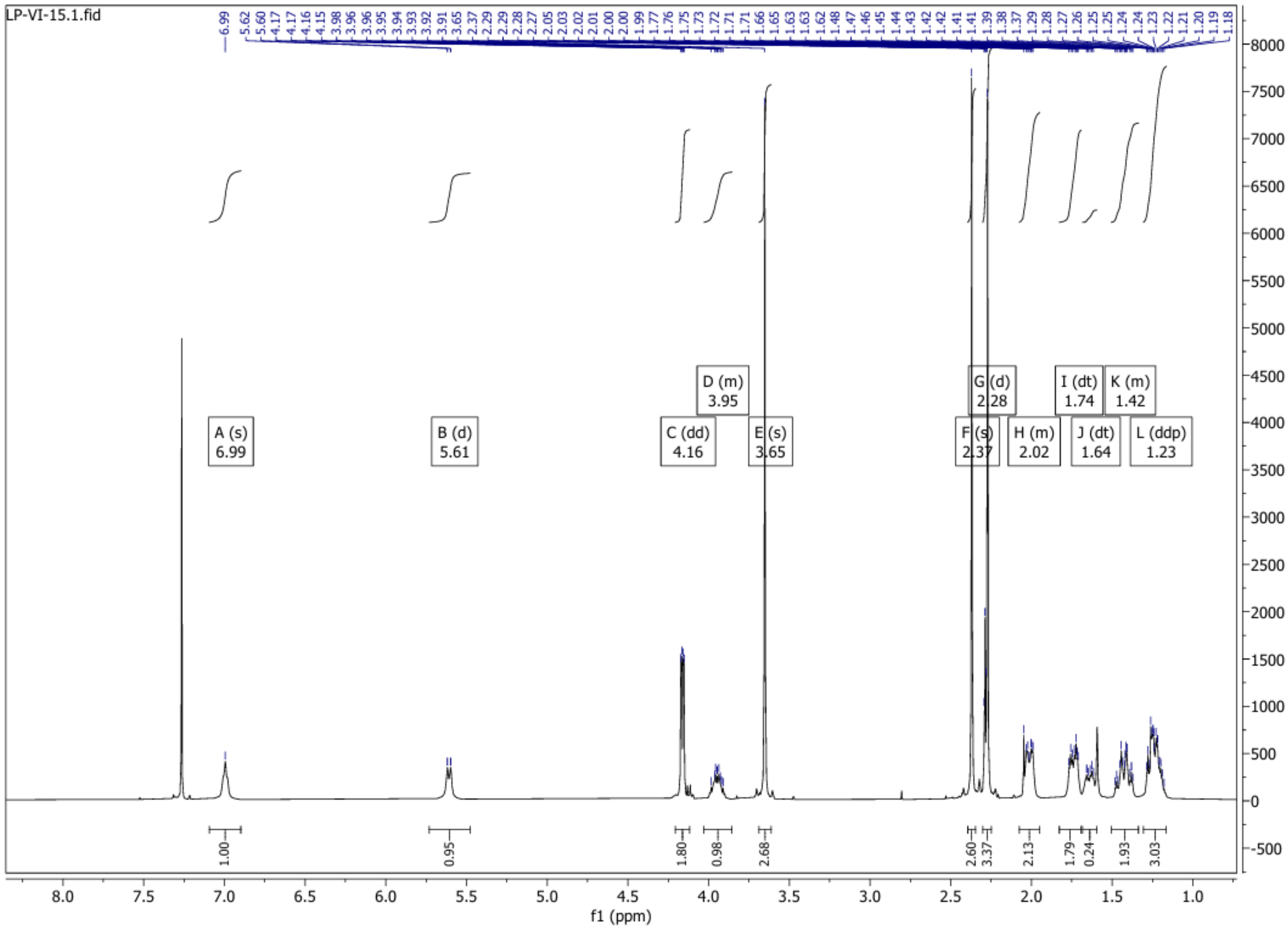
LP-VI-15.1.fid
6.99
5.62 5.60 4.17 4.16 4.15 3.98 3.96 3.96 3.95 3.94 3.93 3.92 3.91 3.65 2.37 2.29 2.29 2.28 2.27 2.05 2.03 2.02 2.01 2.00 2.00 1.99 1.77 1.76 1.75 1.73 1.72 1.71 1.71 1.66 1.65 1.63 1.63 1.62 1.48 1.47 1.46 1.45 1.44 1.43 1.42 1.42 1.41 1.41 1.39 1.38 1.37 1.29 1.28 1.27 1.26 1.25 1.25 1.24 1.24 1.23 1.22 1.21 1.20 1.19 1.18
A (s) 6.99
B (d) 5.61
C (dd) 4.16
D (m) 3.95
E (s) 3.65
F (s) 2.37
G (d) 2.28
H (m) 2.02
I (dt) 1.74
J (dt) 1.64
K (m) 1.42
L (ddp) 1.23
1.00
0.95
1.80
0.98
2.68
2.60
3.37
2.13
1.79
0.24
1.93
3.03
8.0 7.5 7.0 6.5 6.0 5.5 5.0 4.5 4.0 3.5 3.0 2.5 2.0 1.5 1.0
f1 (ppm)
8000 7500 7000 6500 6000 5500 5000 4500 4000 3500 3000 2500 2000 1500 1000 500 0 -500

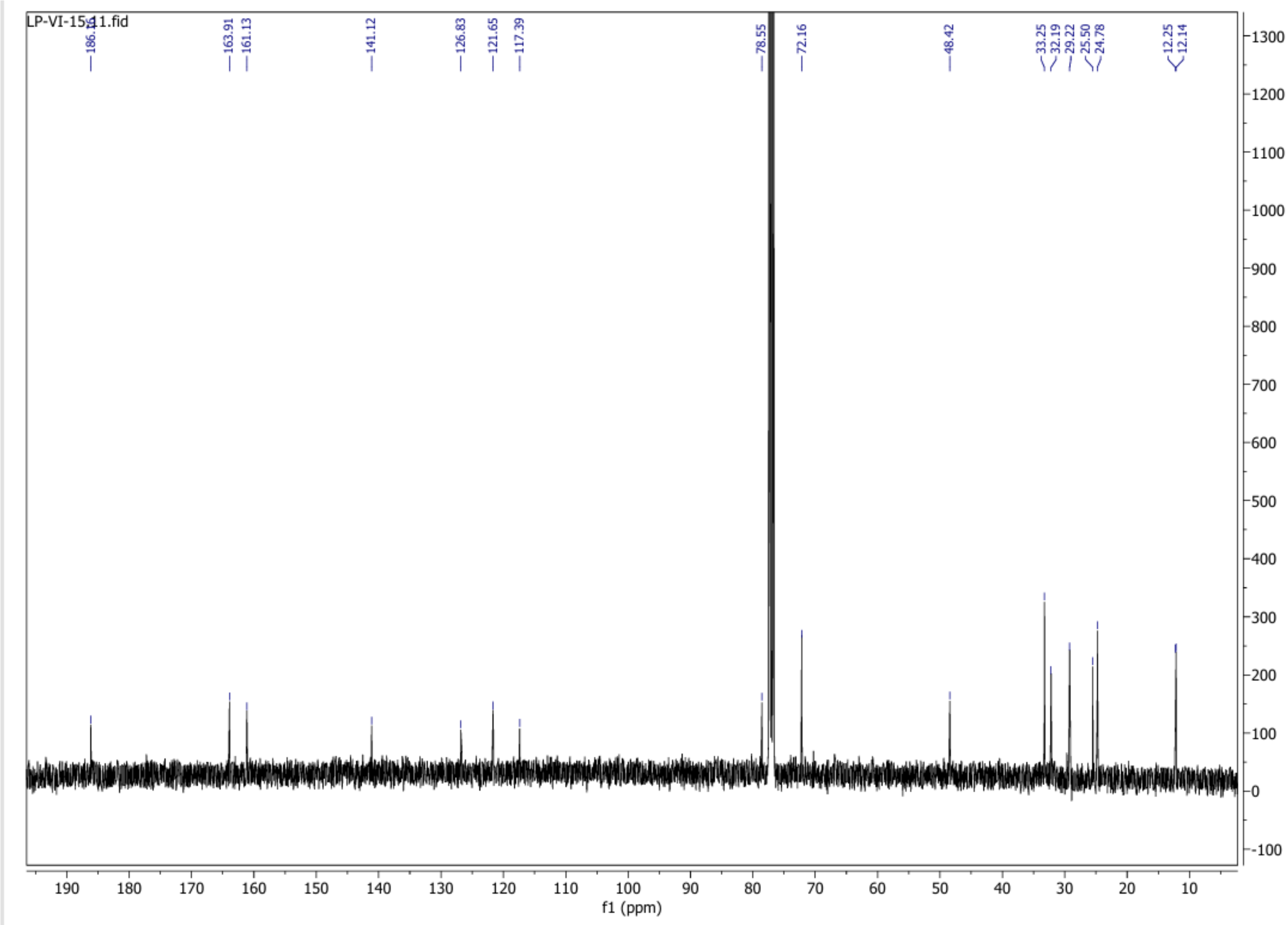

LP-VI-15.11.fid
186.1
163.91
161.13
141.12
126.83
121.65
117.39
78.55
72.16
48.42
33.25
32.19
29.22
25.50
24.78
12.25
12.14
f1 (ppm)

NMR of compound 7b

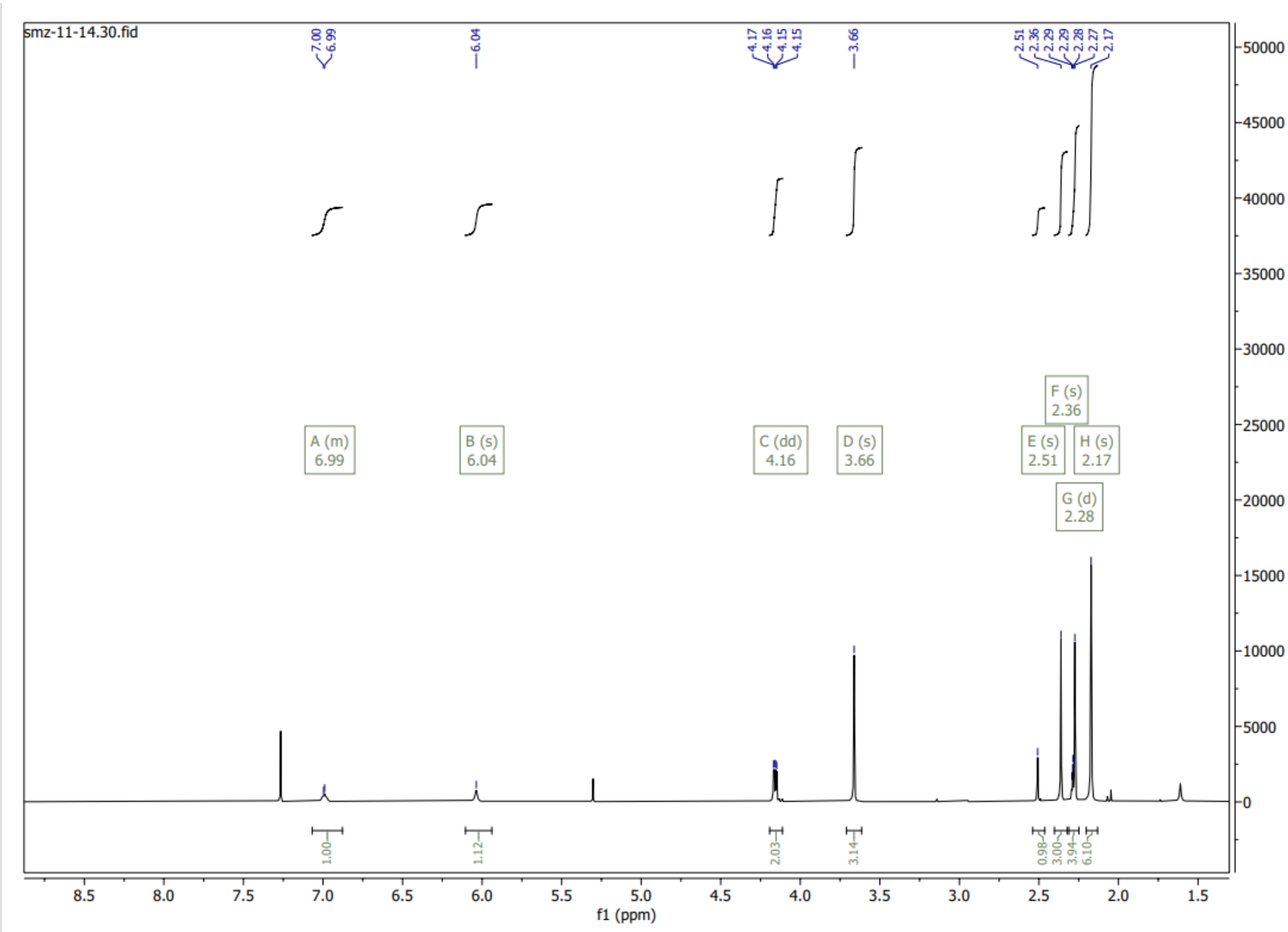

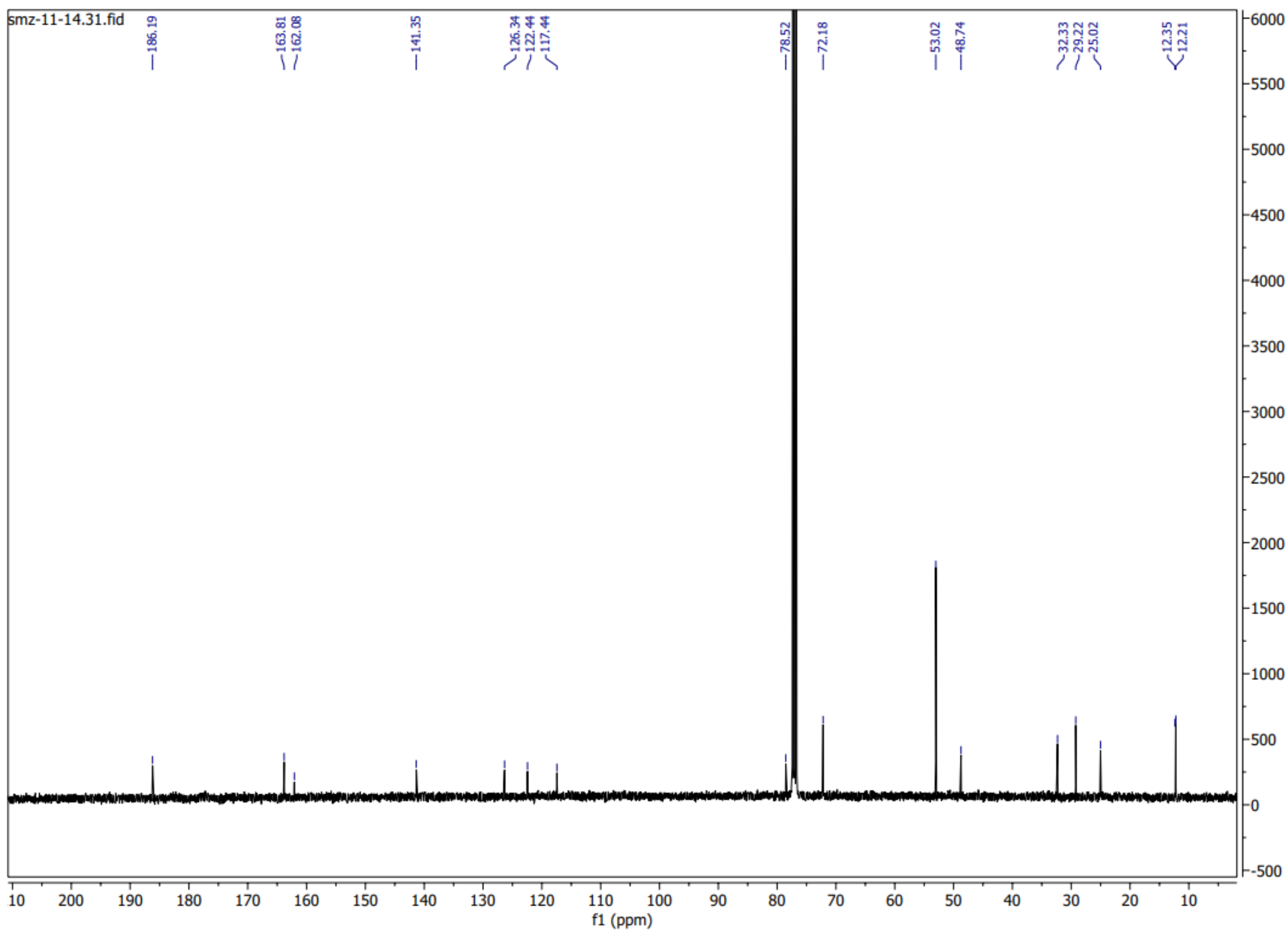

smz-11-14.31.fid
186.19
163.81
162.08
141.35
126.34
122.44
117.44
78.52
72.18
53.02
48.74
32.33
29.22
25.02
12.35
12.21
6000
5500
5000
4500
4000
3500
3000
2500
2000
1500
1000
500
0
-500
10
200
190
180
170
160
150
140
130
120
110
100
90
80
70
60
50
40
30
20
10
f1 (ppm)

NMR of compound 7c

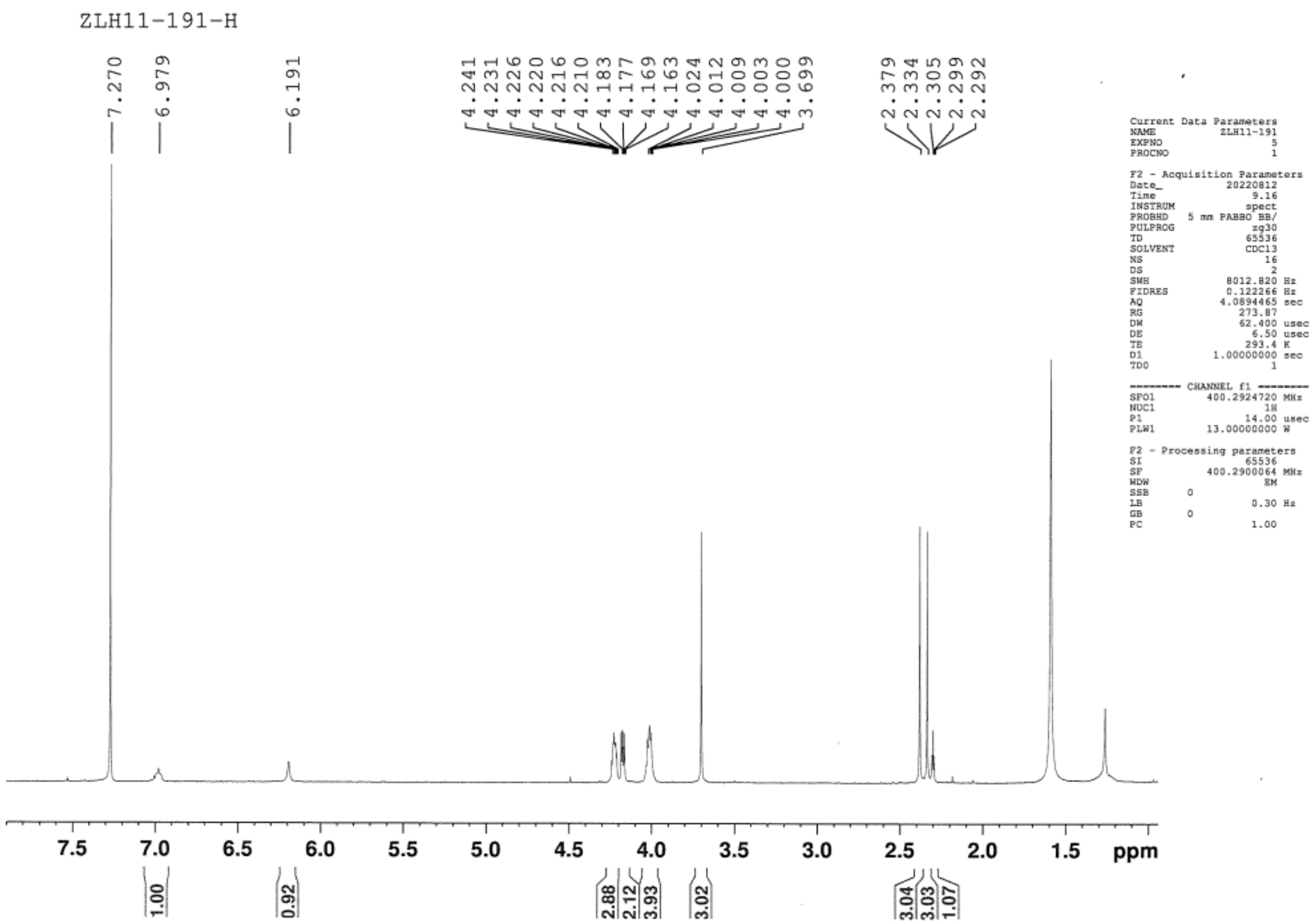

ZLH11-191-C

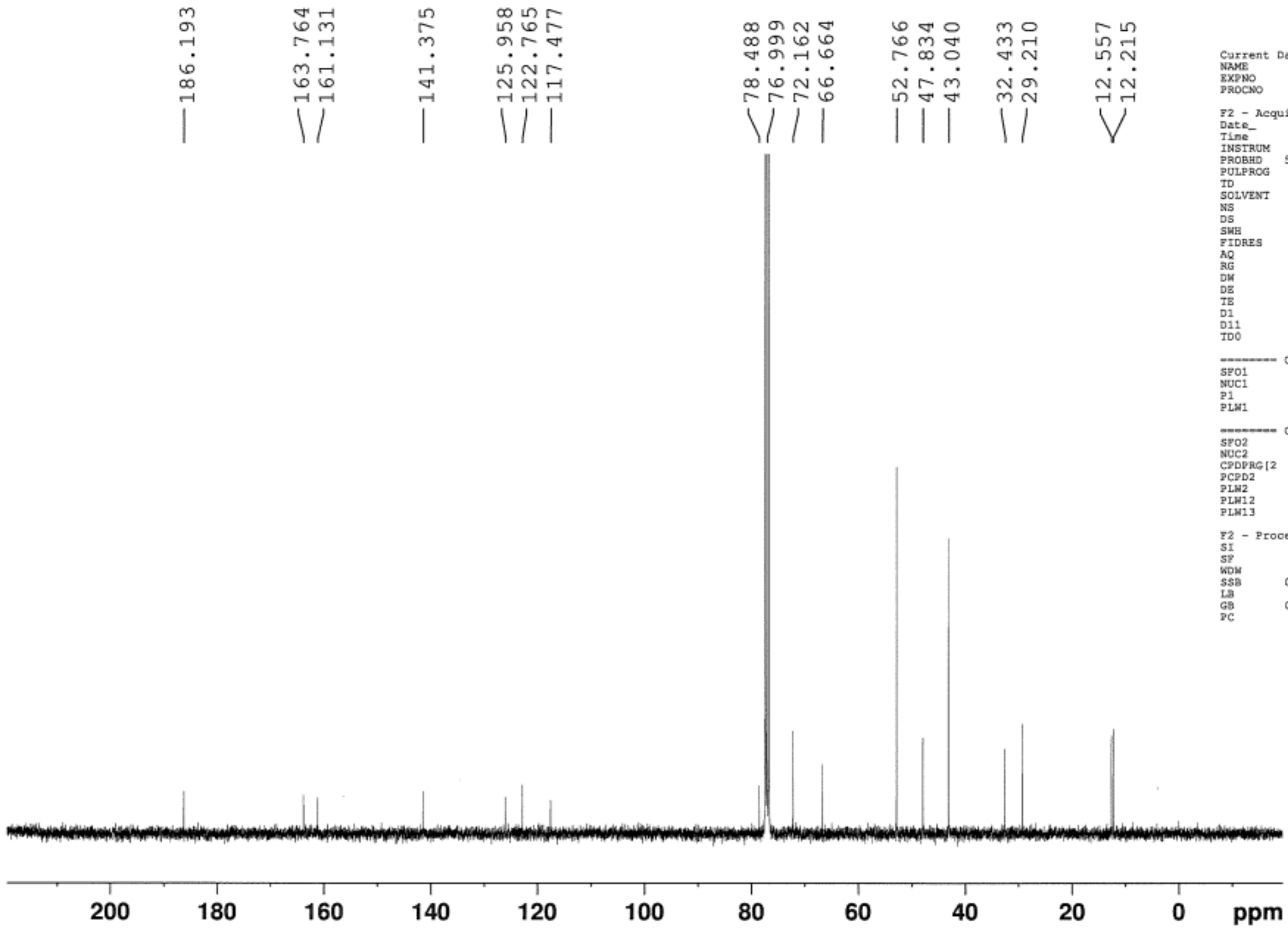


```
Current Data Parameters
NAME             ZLH11-191
EXPNO                   11
PROCNO                   1

F2 - Acquisition Parameters
Date_             20220811
Time                 13.03
INSTRUM              spect
PROBHD     5 mm PABBO BB/
PULPROG            zgpg30
TD                   65536
SOLVENT              CDCl3
NS                    1024
DS                       4
SWH              24038.461 Hz
FIDRES            0.366798 Hz
AQ               1.3631488 sec
RG                  177.06
DW                  20.800 usec
DE                    6.50 usec
TE                   294.6 K
D1              2.00000000 sec
D11             0.03000000 sec
TD0                      1

======== CHANNEL f1 ========
SFO1           100.6630654 MHz
NUC1                   13C
P1                   10.00 usec
PLW1           53.00000000 W

======== CHANNEL f2 ========
SFO2           400.2916012 MHz
NUC2                    1H
CPDPRG[2           waltz16
PCPD2                90.00 usec
PLW2           13.00000000 W
PLW12           0.31457001 W
PLW13           0.25479999 W

F2 - Processing parameters
SI                   32768
SF             100.6530039 MHz
WDW                     EM
SSB      0
LB                    1.00 Hz
GB       0
PC                    1.40
```

NMR of compound 7d

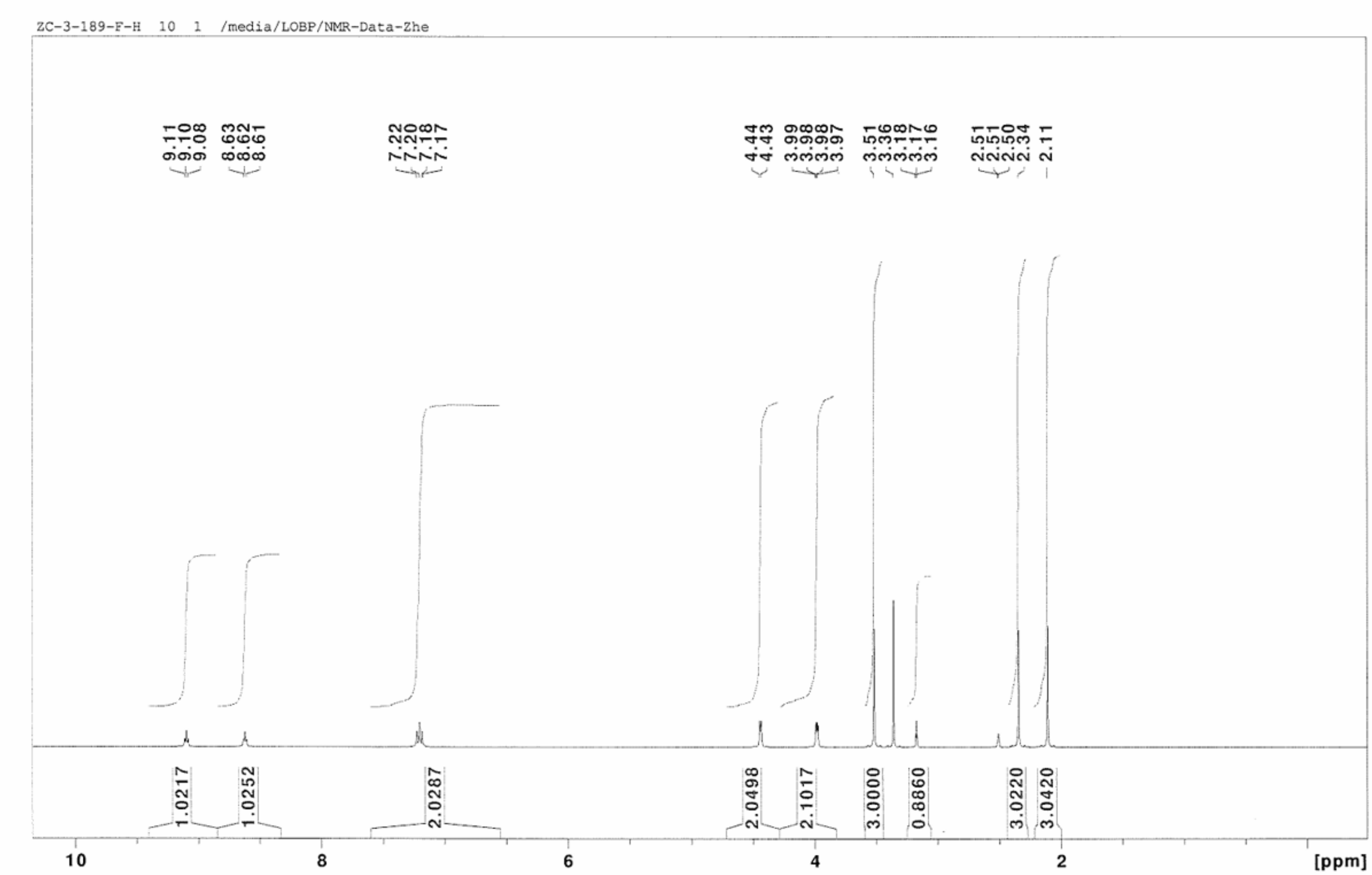

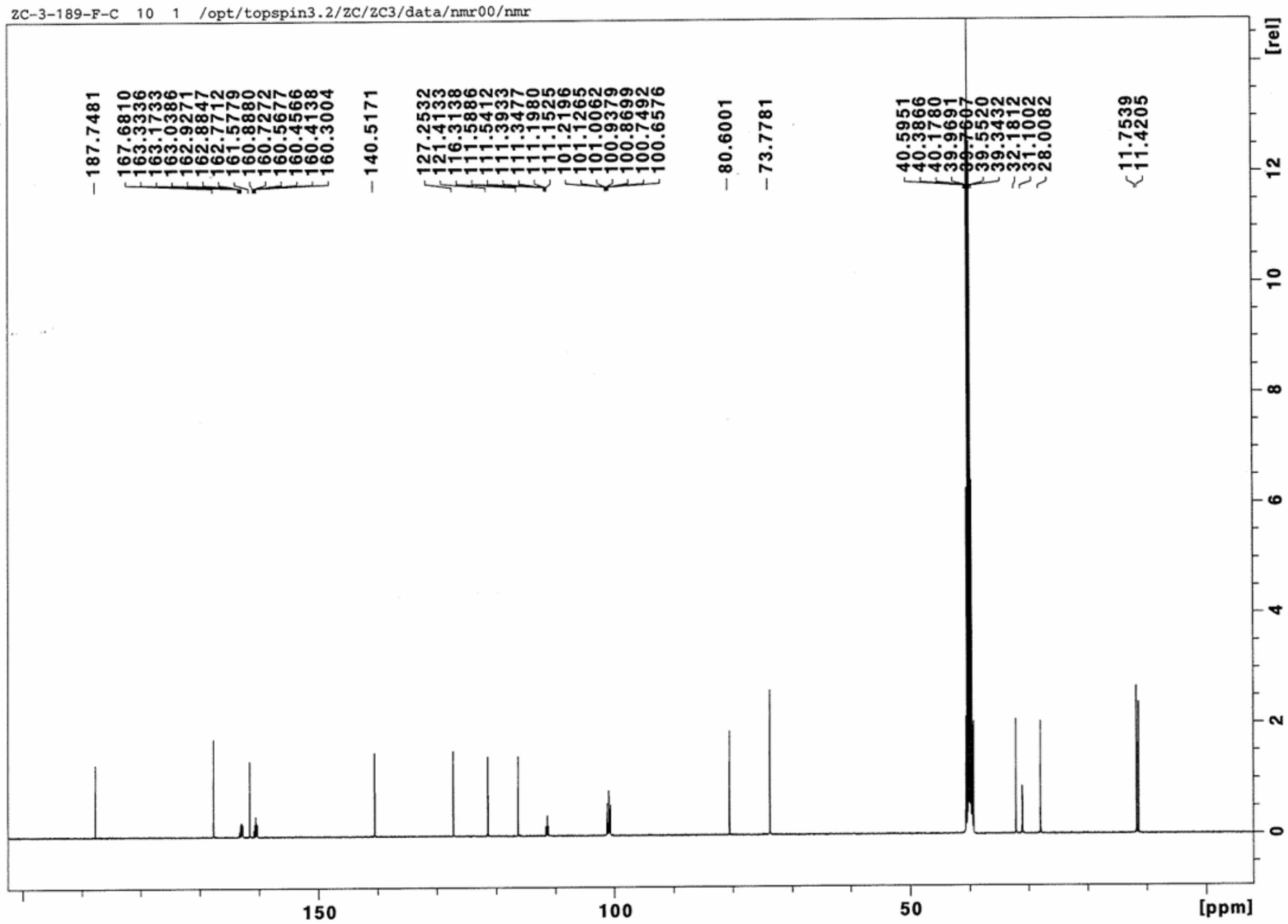
ZC-3-189-F-C 10 1 /opt/topspin3.2/ZC/ZC3/data/nmr00/nmr
187.7481
167.6810
163.3336
163.1733
163.0386
162.9271
162.8847
162.7712
161.5779
160.8880
160.7272
160.5677
160.4566
160.4138
160.3004
140.5171
127.2532
121.4133
116.3138
111.5886
111.5412
111.3933
111.3477
111.1980
111.1525
101.2196
101.1265
101.0062
100.9379
100.8699
100.7492
100.6576
80.6001
73.7781
40.5951
40.3866
40.1780
39.9691
39.7607
39.5520
39.3432
32.1812
31.1002
28.0082
11.7539
11.4205
[rel]
12
10
8
6
4
2
0
150
100
50
[ppm]

ZC-3-189-F-F 10 1 /opt/topspin3.2/ZC/ZC3/data/nmr00/nmr

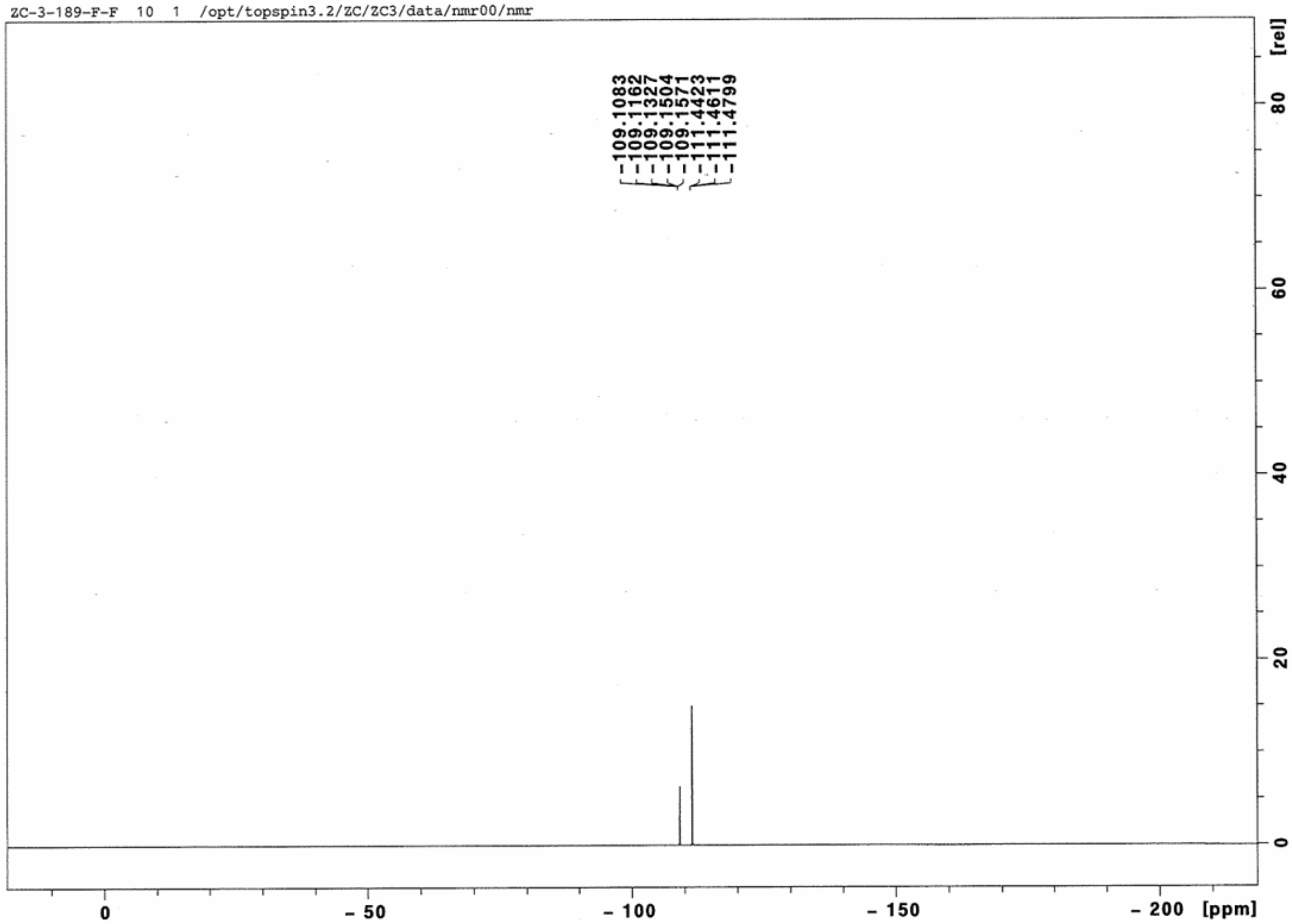

NMR of compound 7e

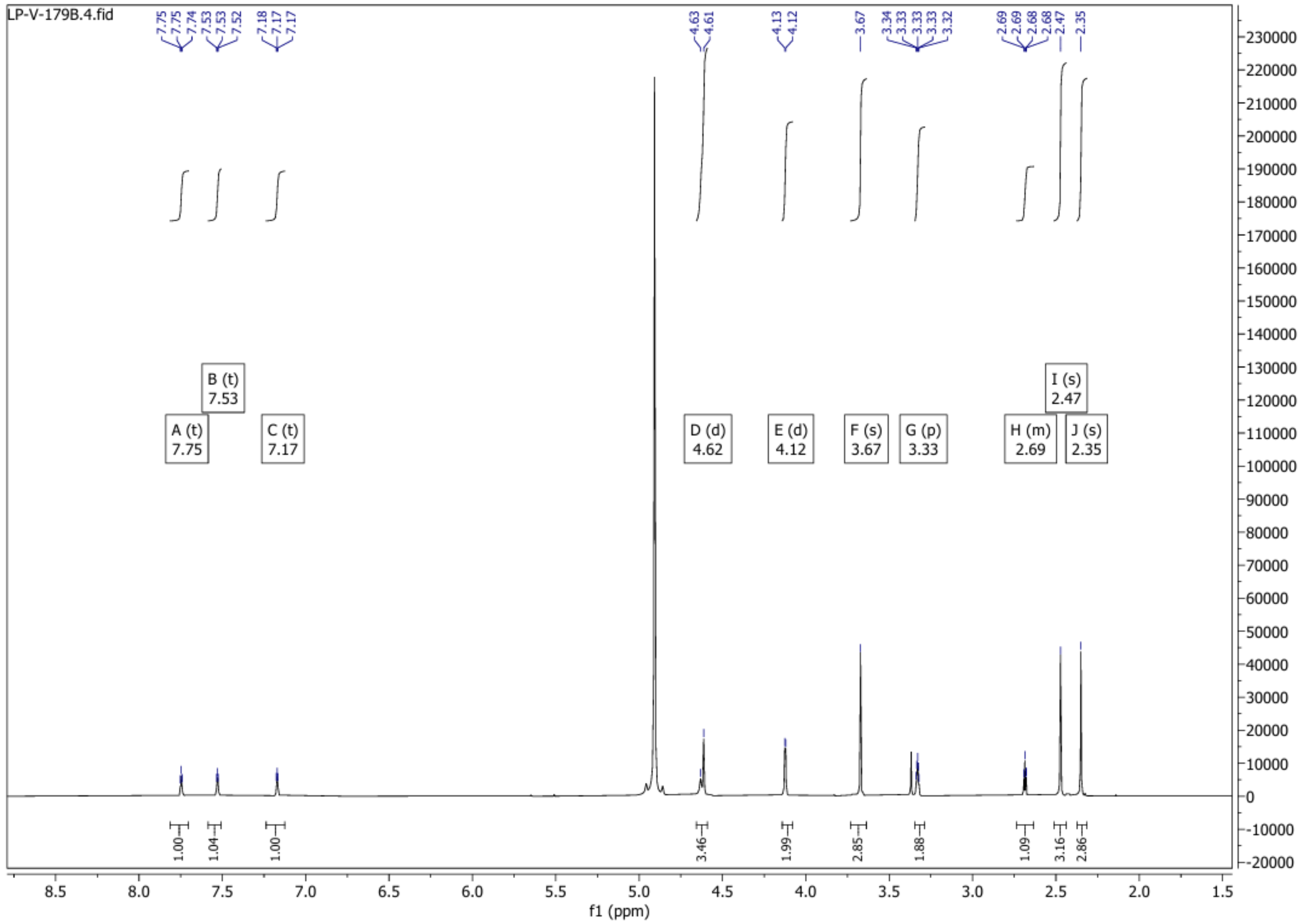

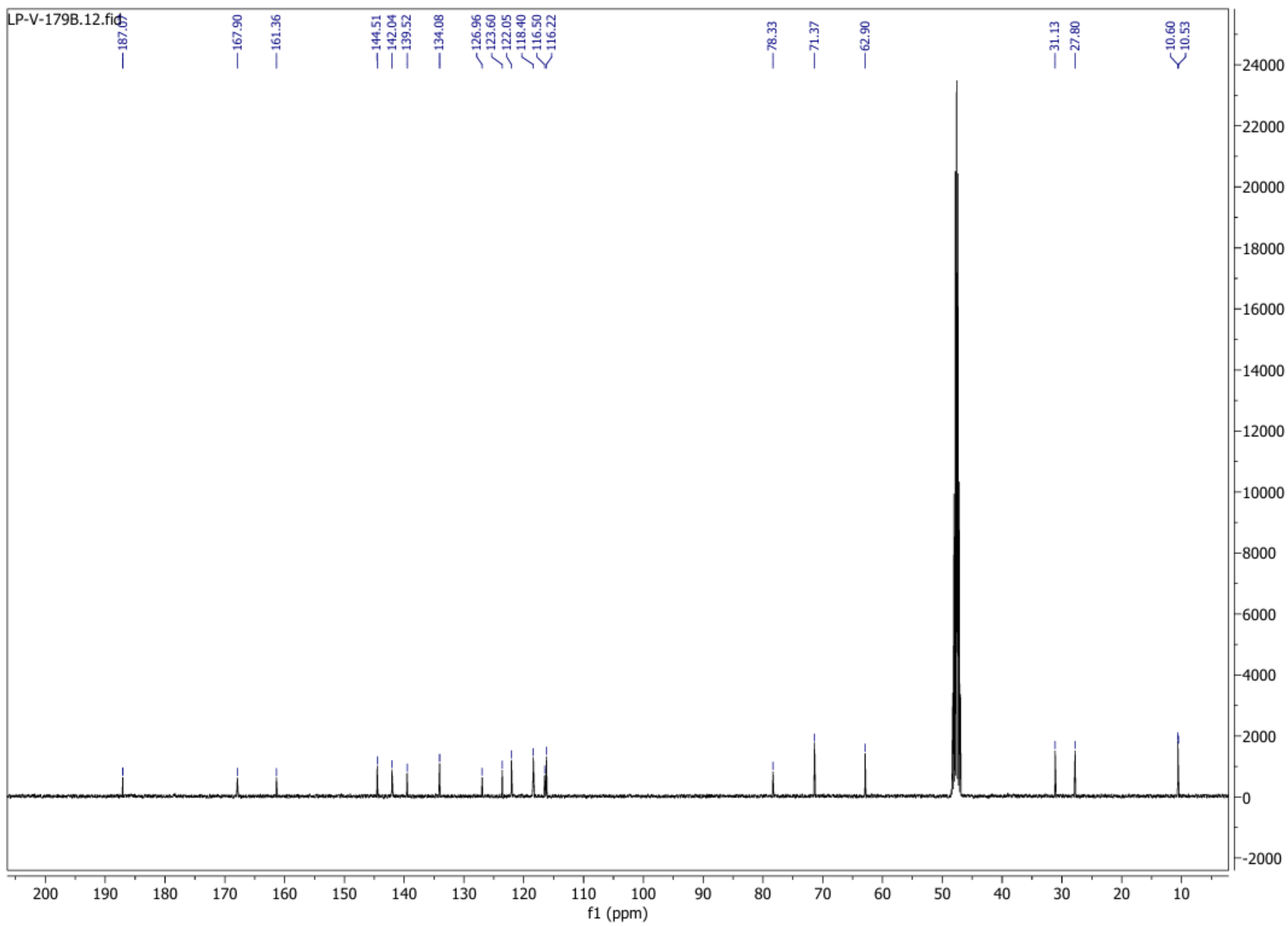
LP-V-179B.12.fid
187.07
167.90
161.36
144.51
142.04
139.52
134.08
126.96
123.60
122.05
118.40
116.50
116.22
78.33
71.37
62.90
31.13
27.80
10.60
10.53
f1 (ppm)

NMR of compound 7f

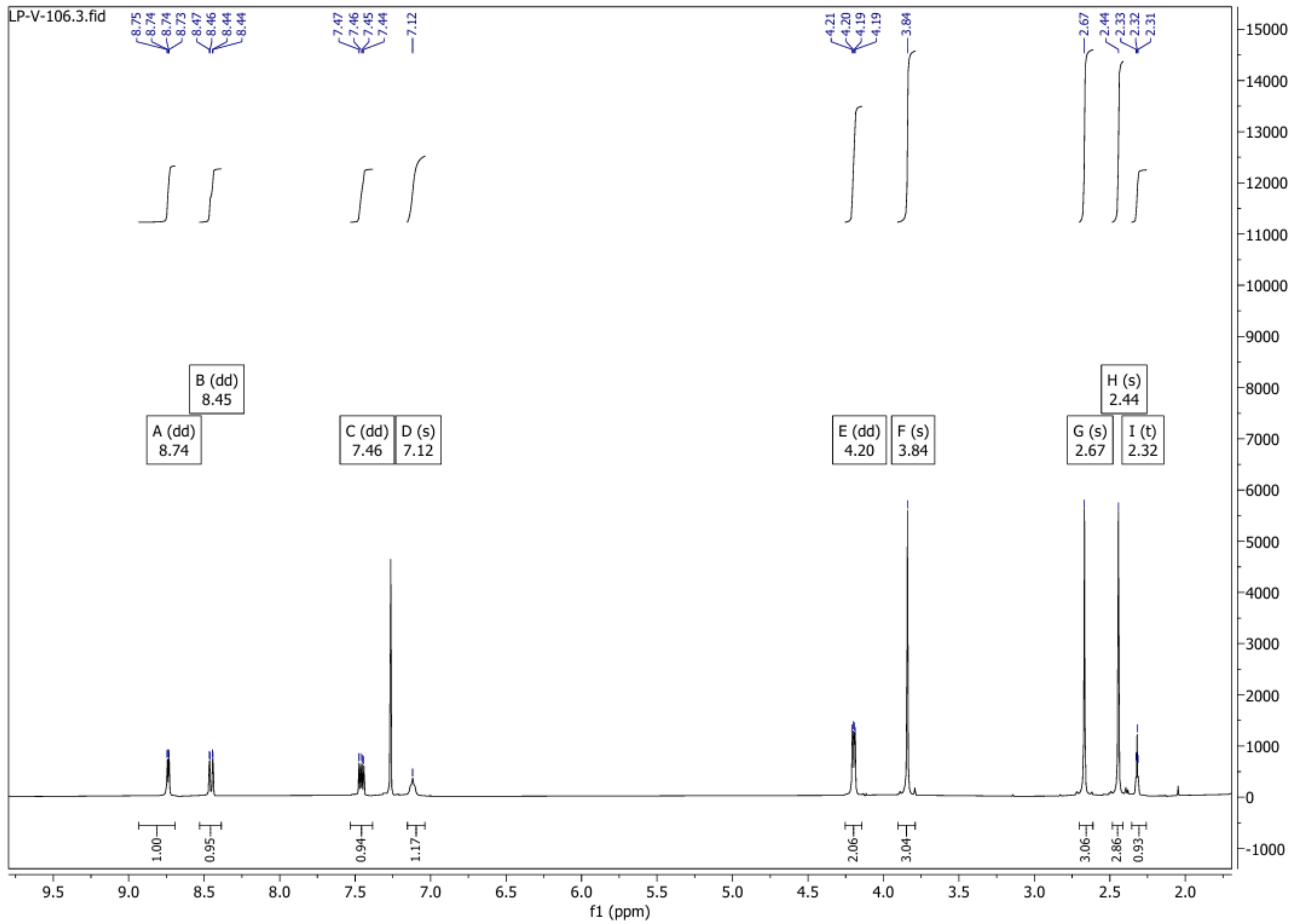

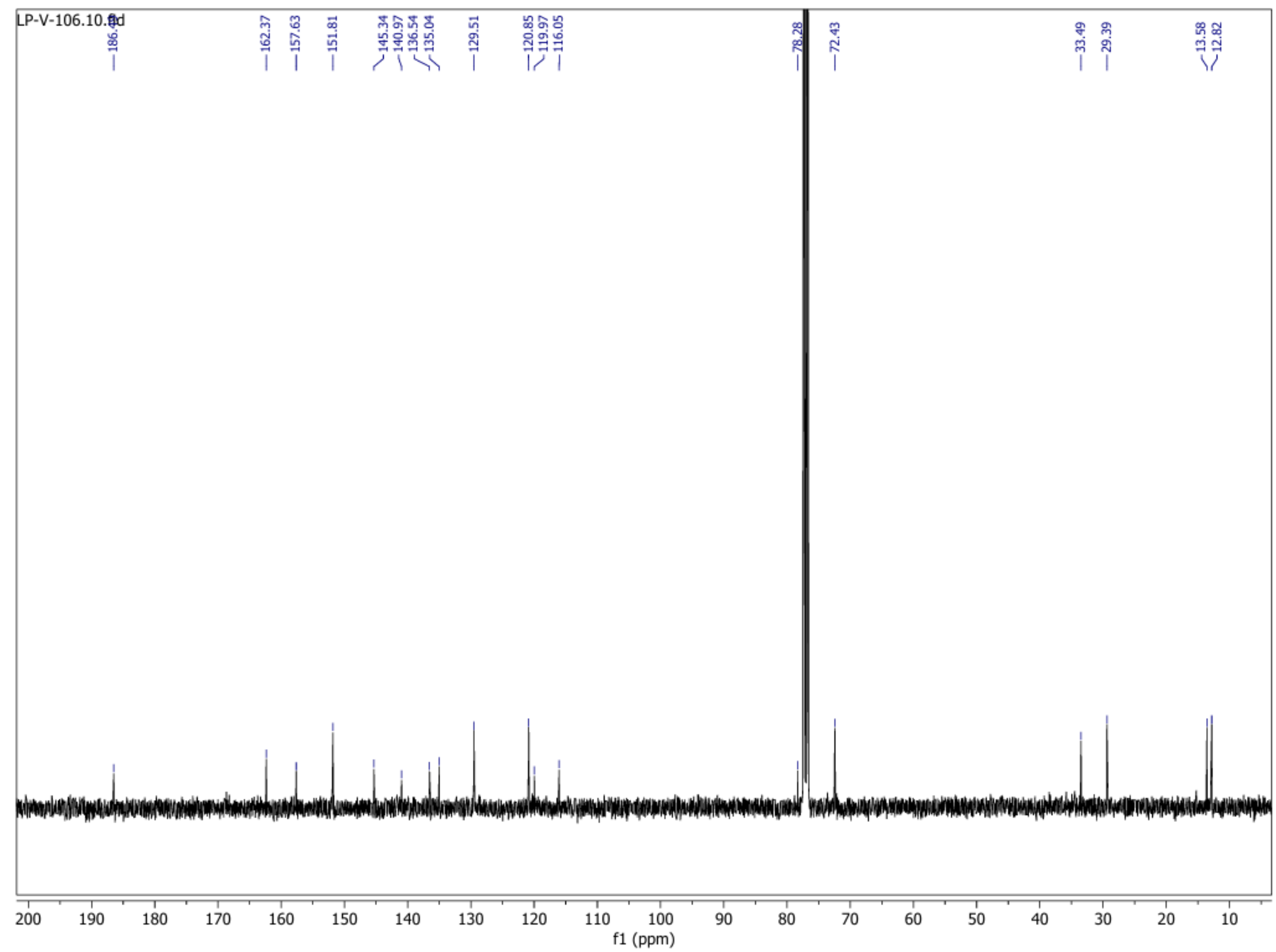

LP-V-106.10.fid
186.40
162.37
157.63
151.81
145.34
140.97
136.54
135.04
129.51
120.85
119.97
116.05
78.28
72.43
33.49
29.39
13.58
12.82
f1 (ppm)

NMR of compound 7g

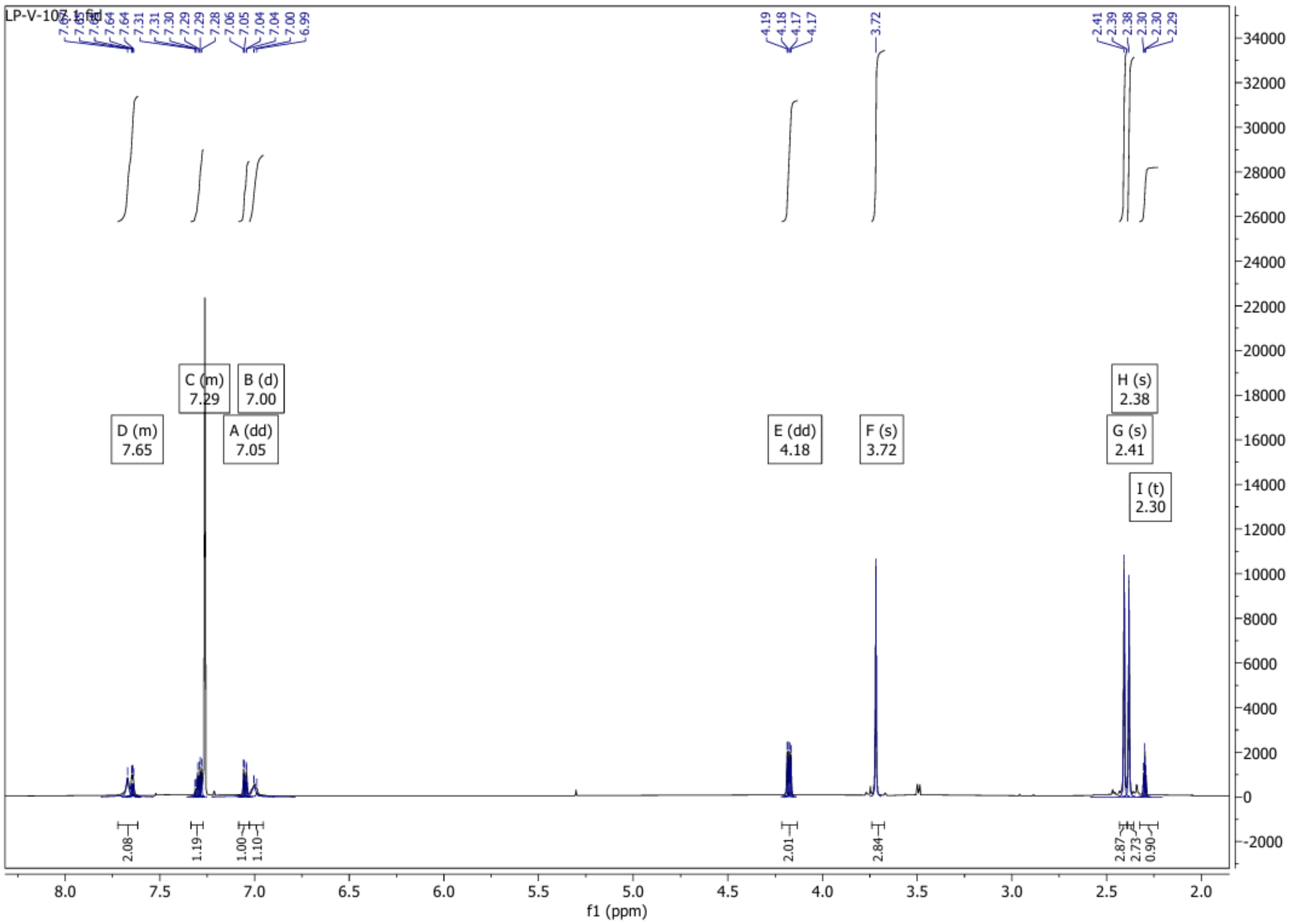

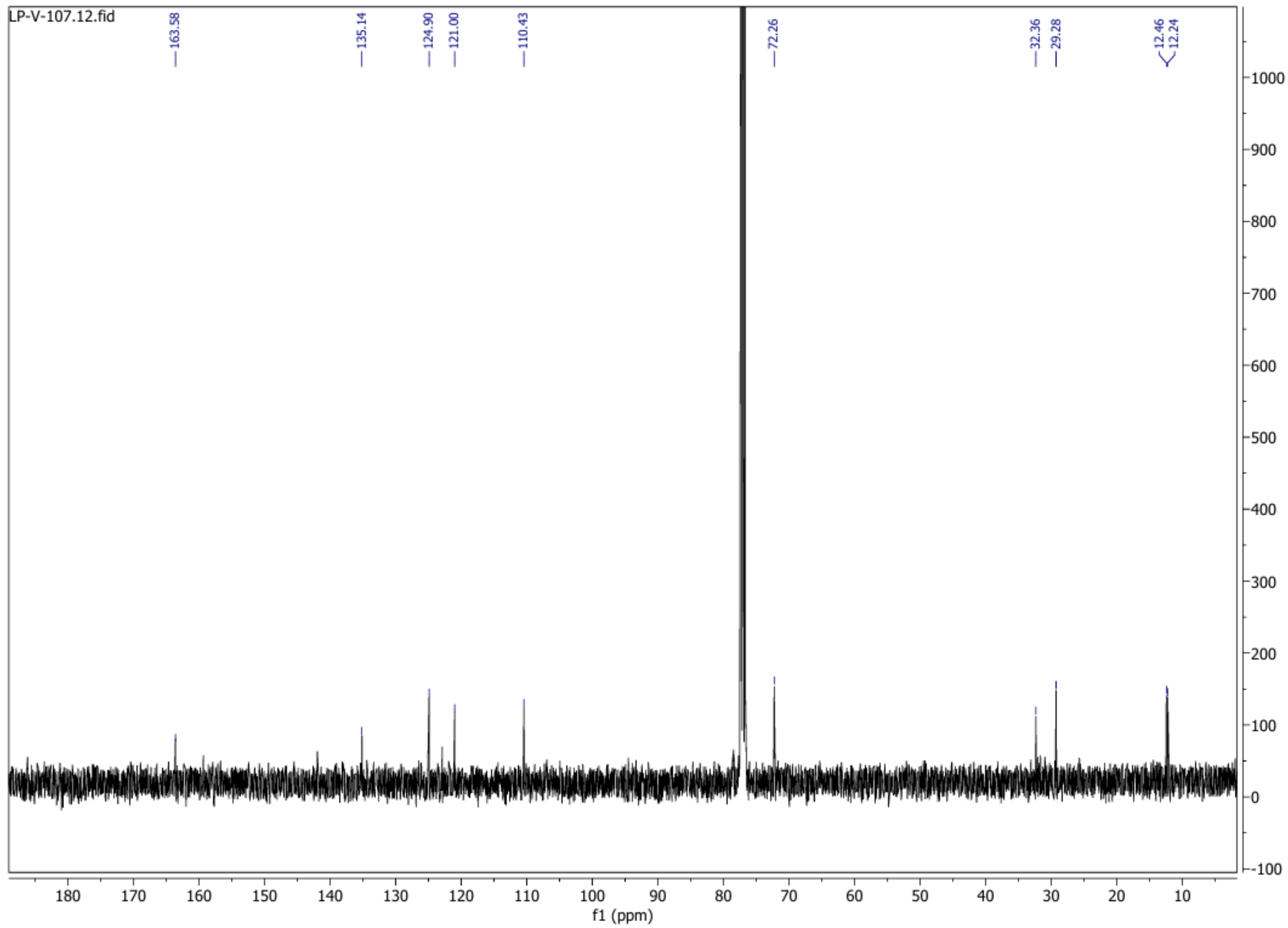

LP-V-107.12.fid
163.58
135.14
124.90
121.00
110.43
72.26
32.36
29.28
12.46
12.24
f1 (ppm)

NMR of compound 7h

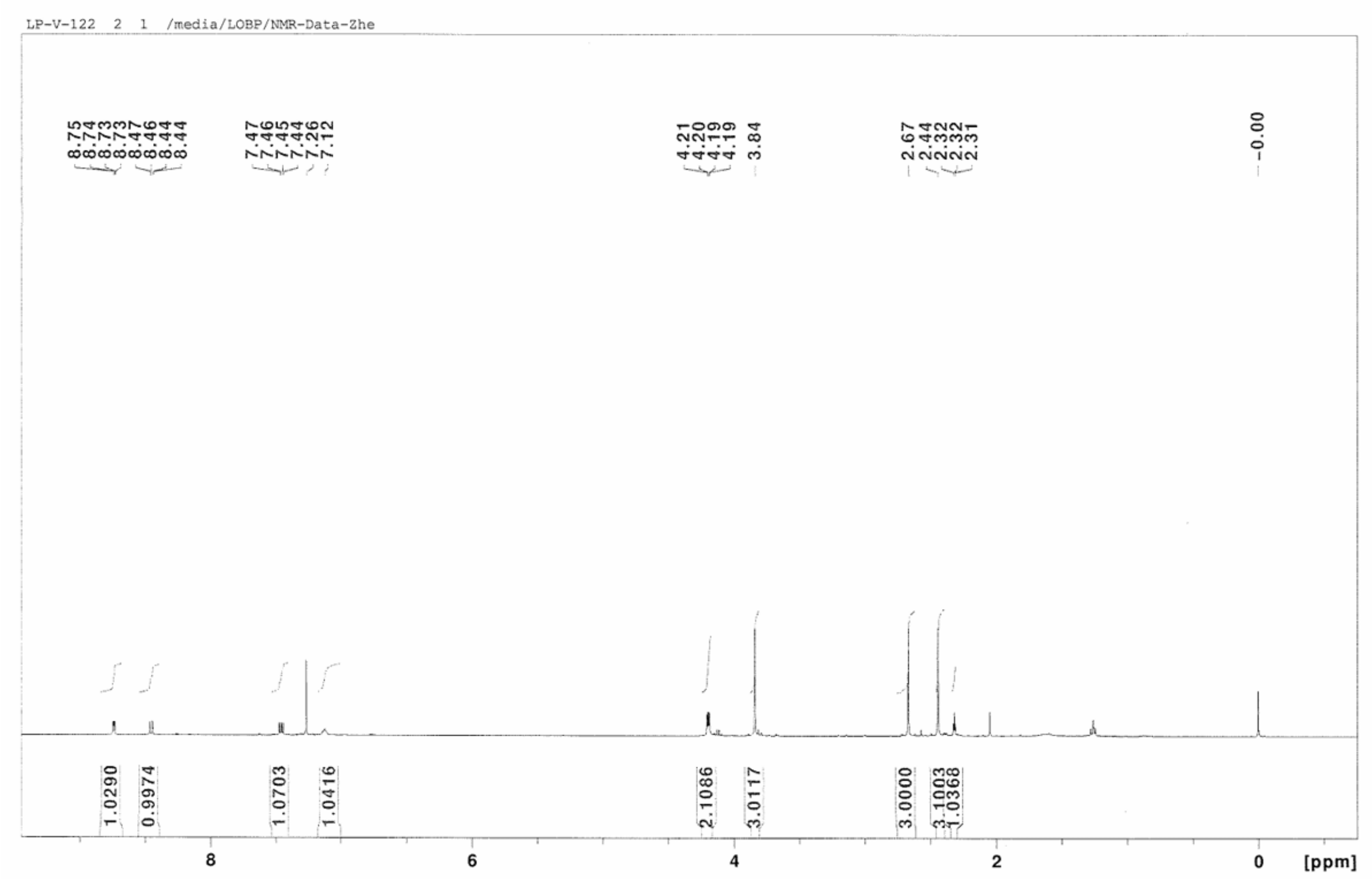

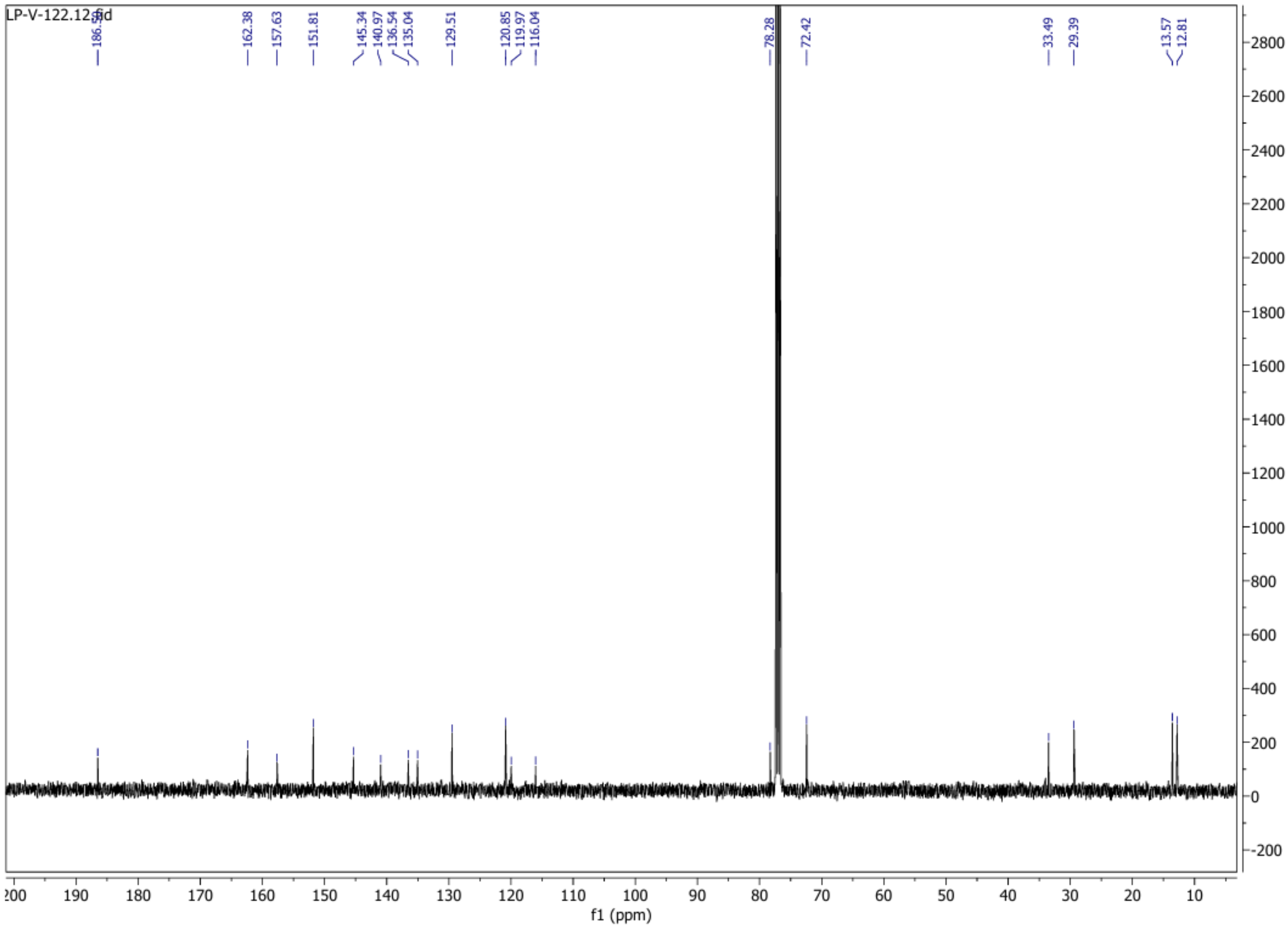
LP-V-122.12.fid
186.50
162.38
157.63
151.81
145.34
140.97
136.54
135.04
129.51
120.85
119.97
116.04
78.28
72.42
33.49
29.39
13.57
12.81
f1 (ppm)

NMR of compound 7i

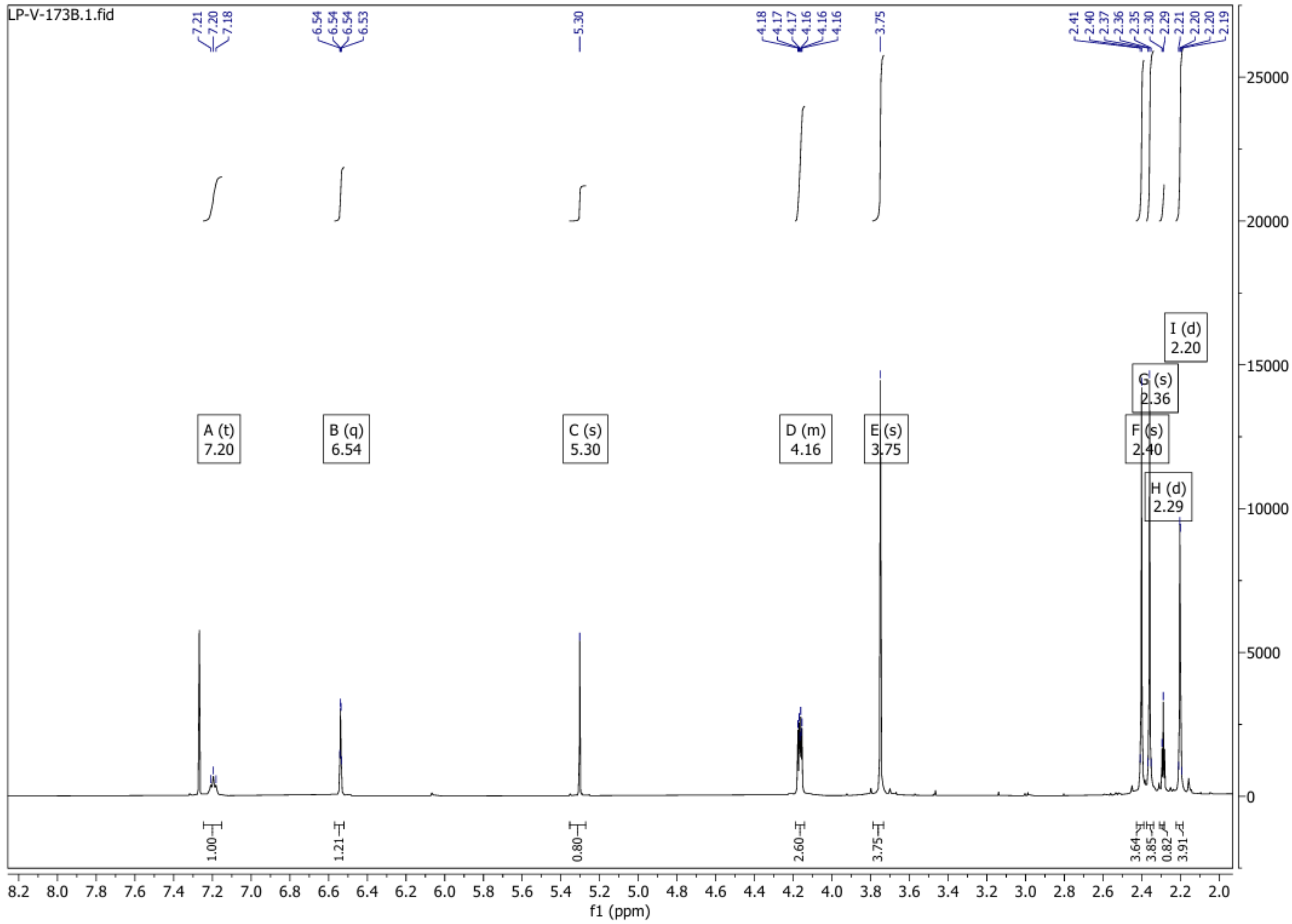

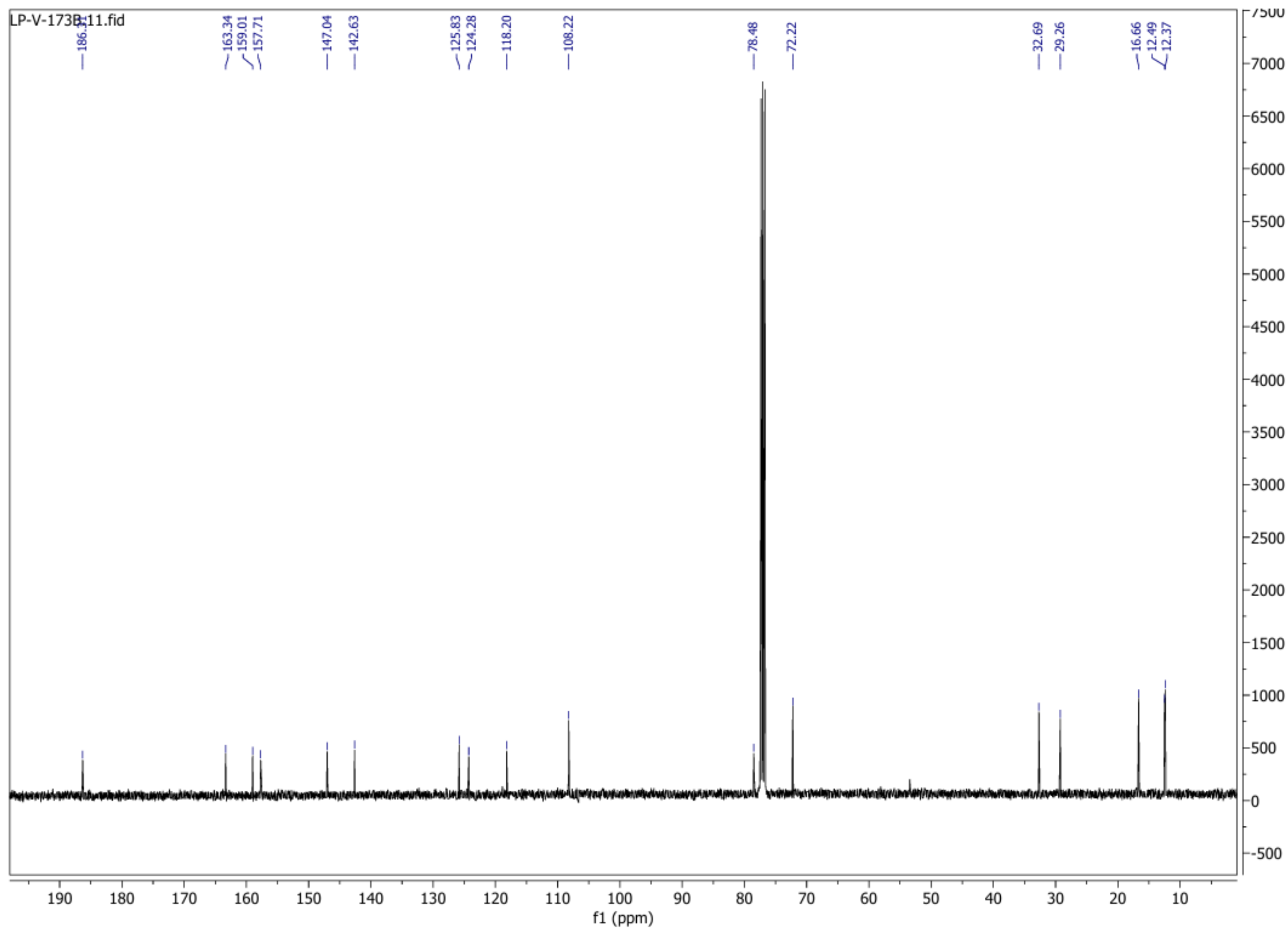

LP-V-173B.11.fid
186.1
163.34
159.01
157.71
147.04
142.63
125.83
124.28
118.20
108.22
78.48
72.22
32.69
29.26
16.66
12.49
12.37
f1 (ppm)

NMR of compound 7j

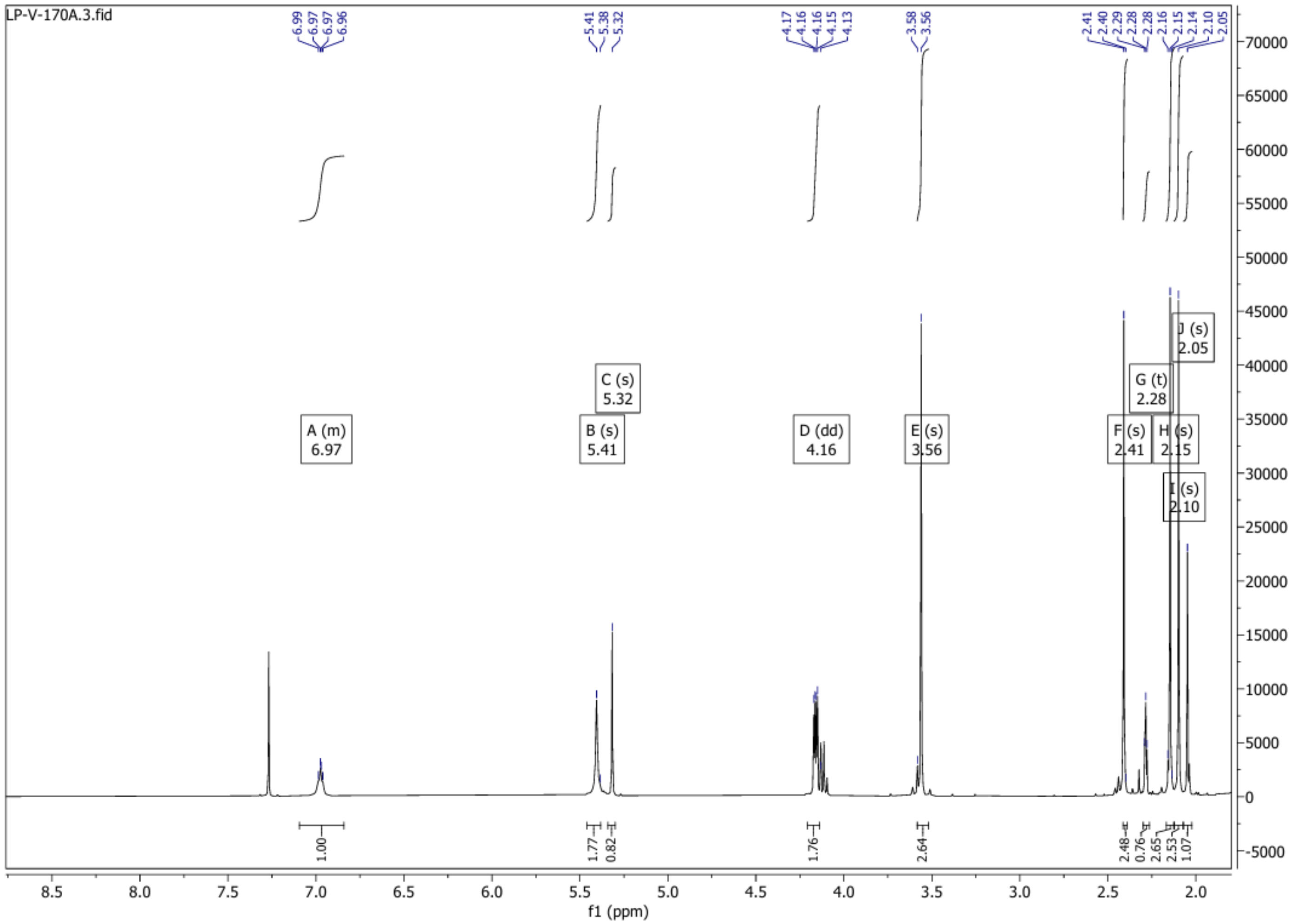

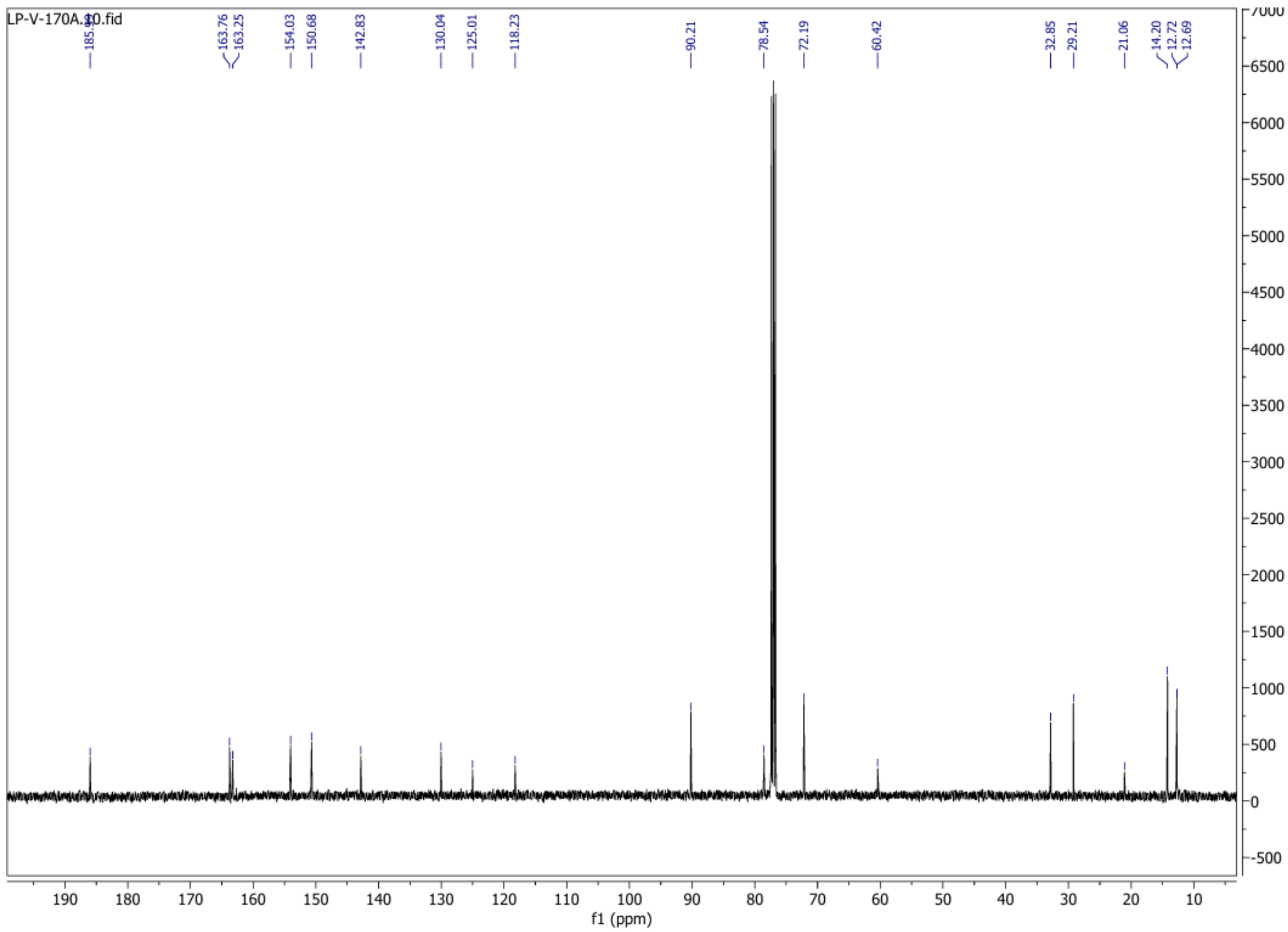

LP-V-170A.10.fid
185.90
163.76
163.25
154.03
150.68
142.83
130.04
125.01
118.23
90.21
78.54
72.19
60.42
32.85
29.21
21.06
14.20
12.72
12.69
f1 (ppm)

NMR of compound 7k

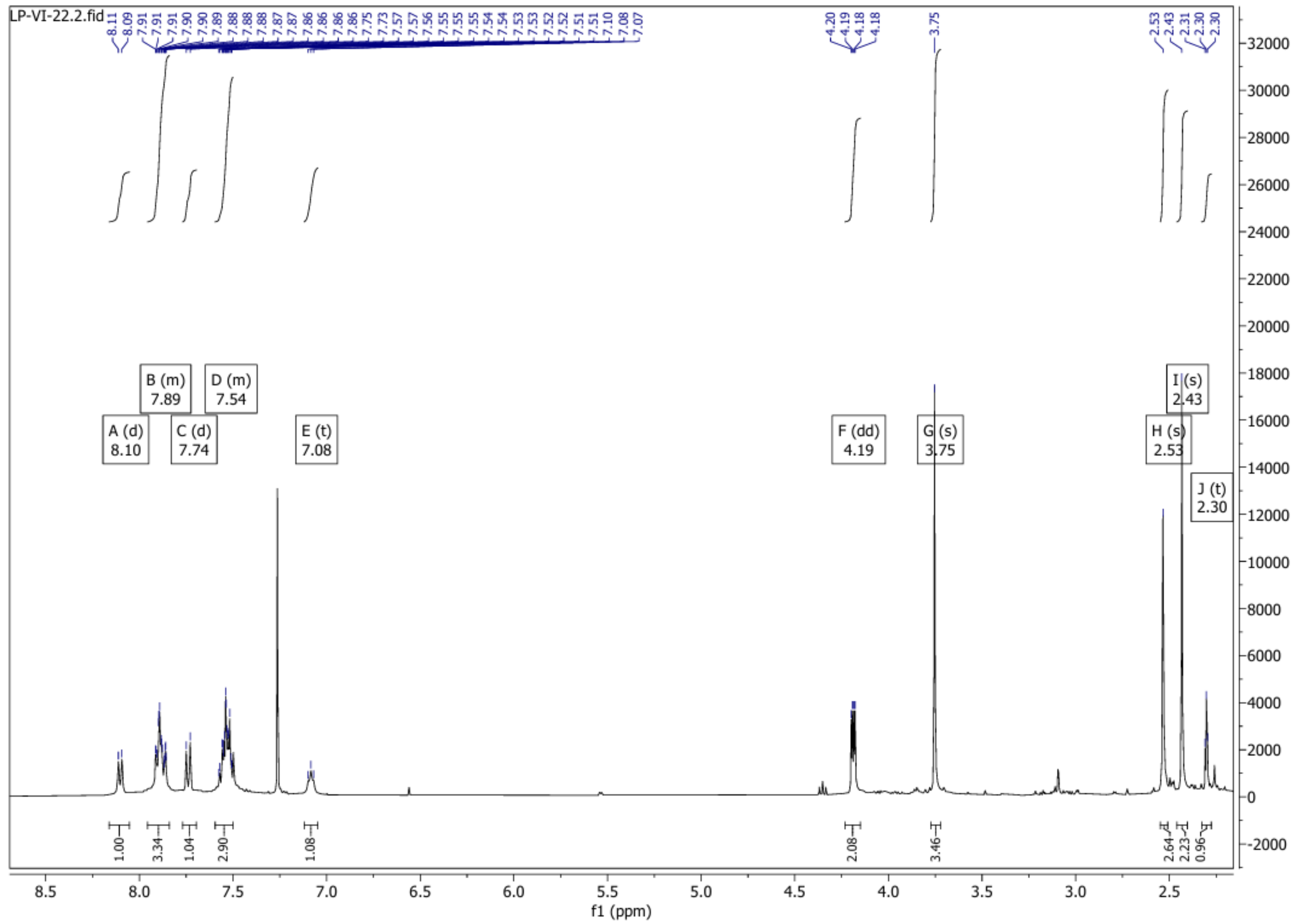

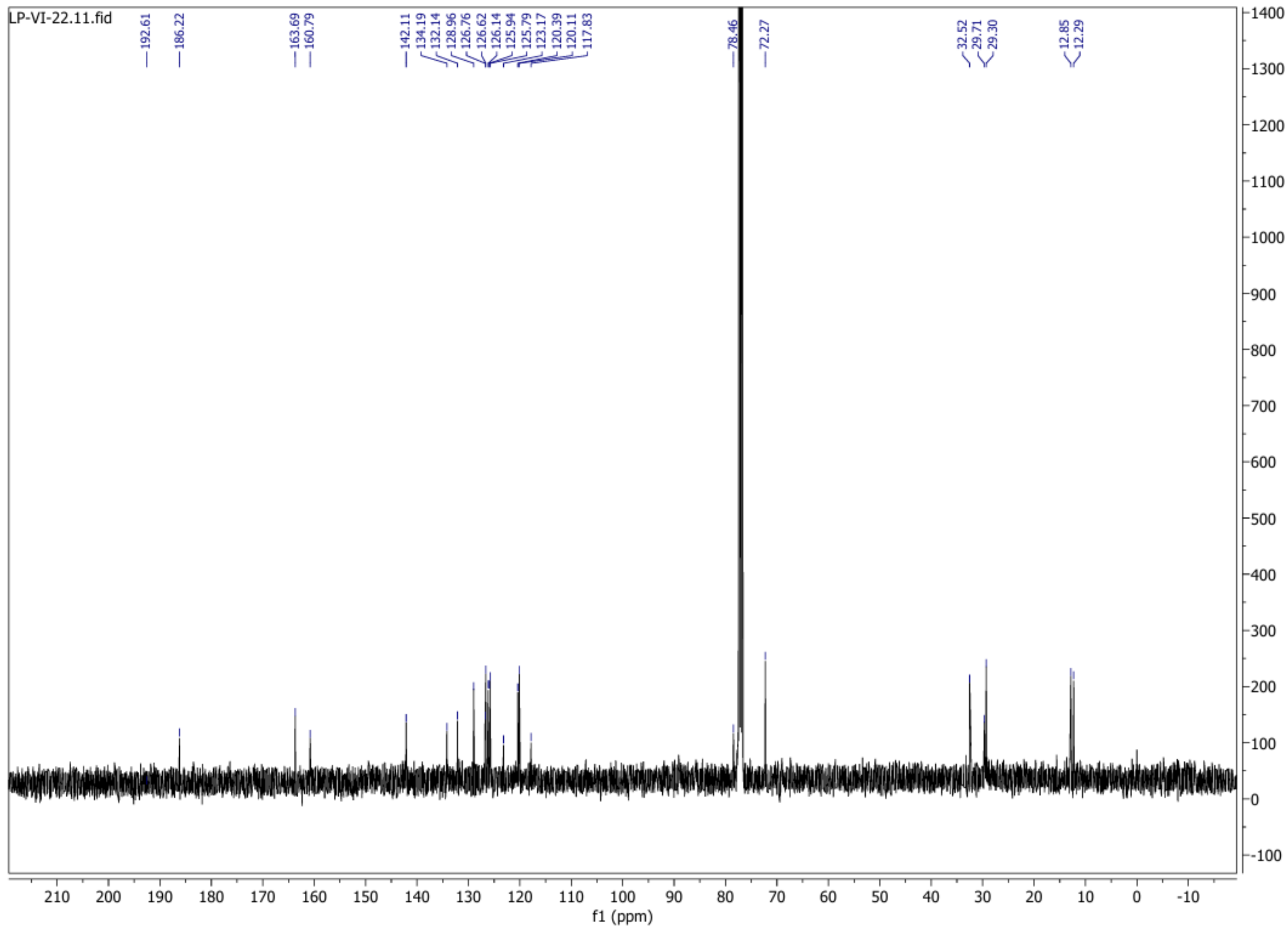
LP-VI-22.11.fid
192.61
186.22
163.69
160.79
142.11
134.19
132.14
128.96
126.76
126.62
126.14
125.94
125.79
123.17
120.39
120.11
117.83
78.46
72.27
32.52
29.71
29.30
12.85
12.29
f1 (ppm)

NMR of compound 7l

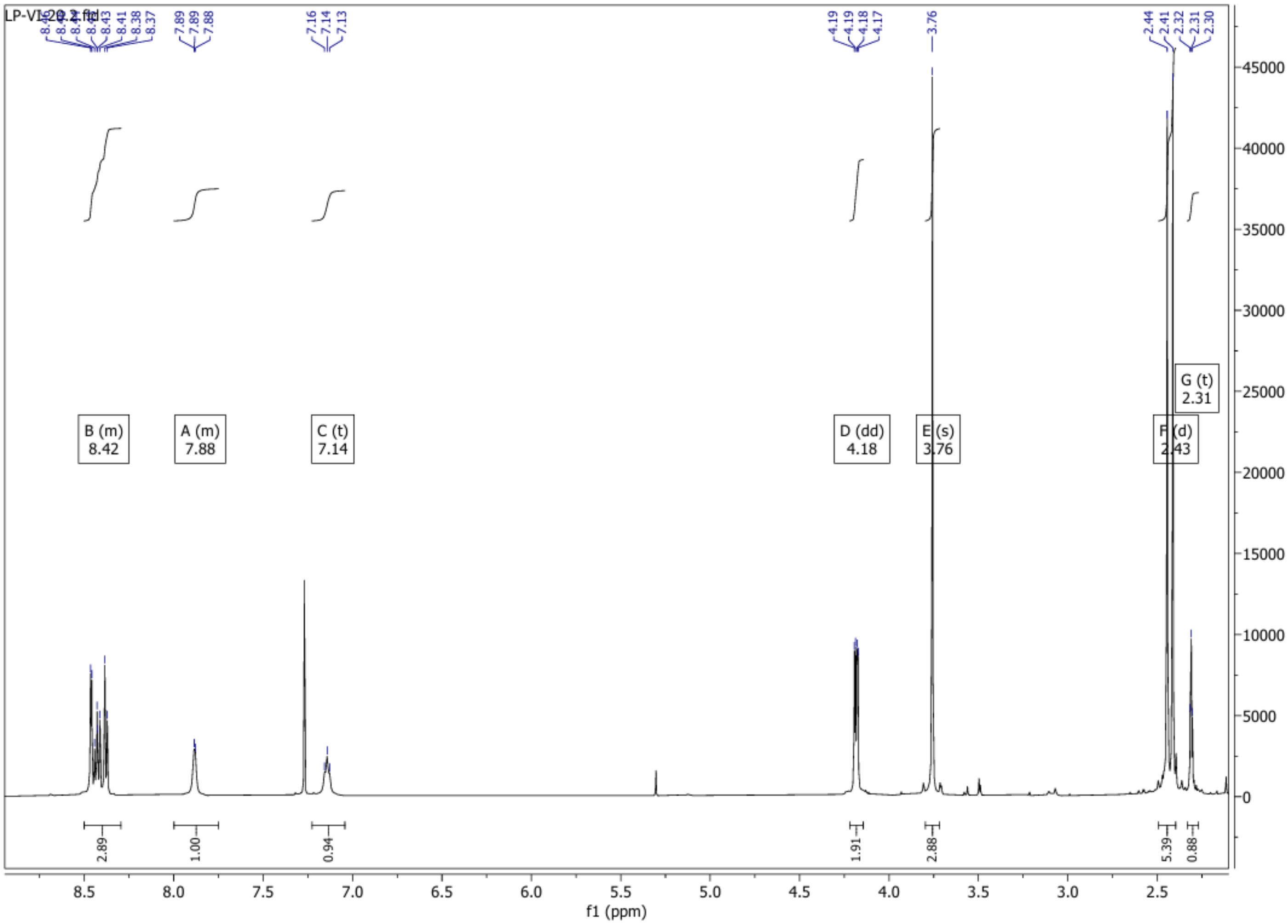

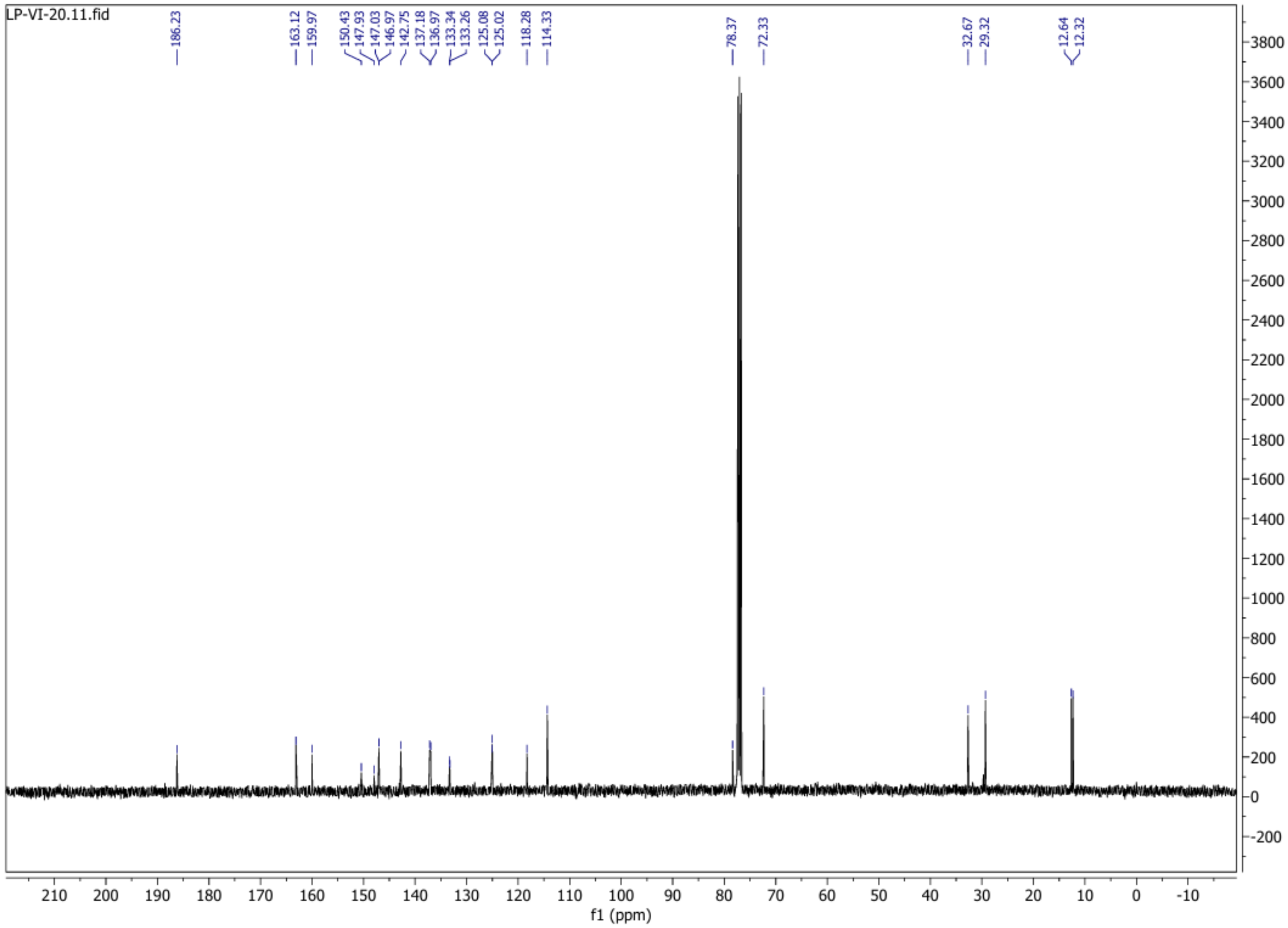
LP-VI-20.11.fid
186.23
163.12
159.97
150.43
147.93
147.03
146.97
142.75
137.18
136.97
133.34
133.26
125.08
125.02
118.28
114.33
78.37
72.33
32.67
29.32
12.64
12.32
3800
3600
3400
3200
3000
2800
2600
2400
2200
2000
1800
1600
1400
1200
1000
800
600
400
200
0
-200
210 200 190 180 170 160 150 140 130 120 110 100 90 80 70 60 50 40 30 20 10 0 -10
f1 (ppm)

NMR of compound 7m

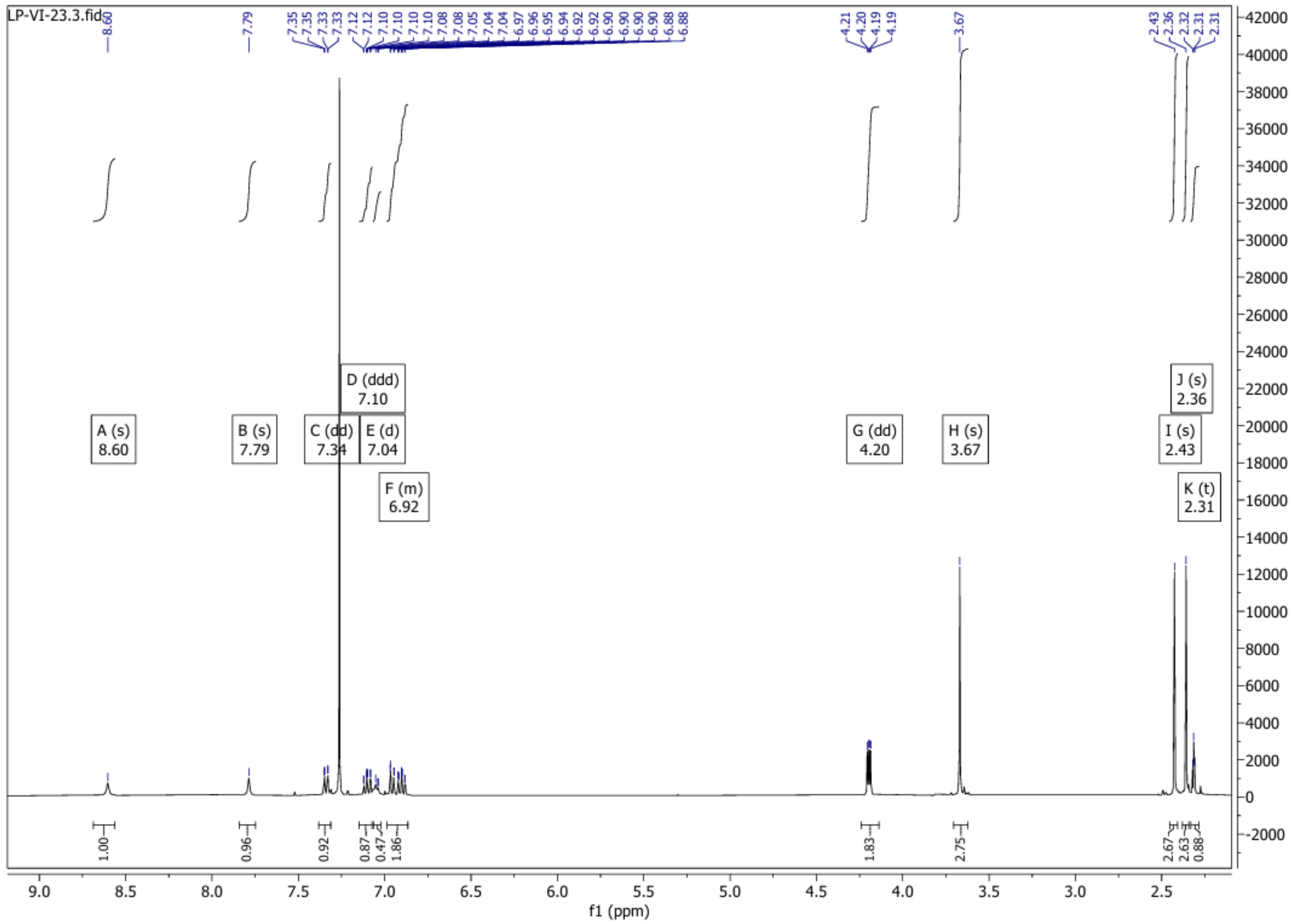

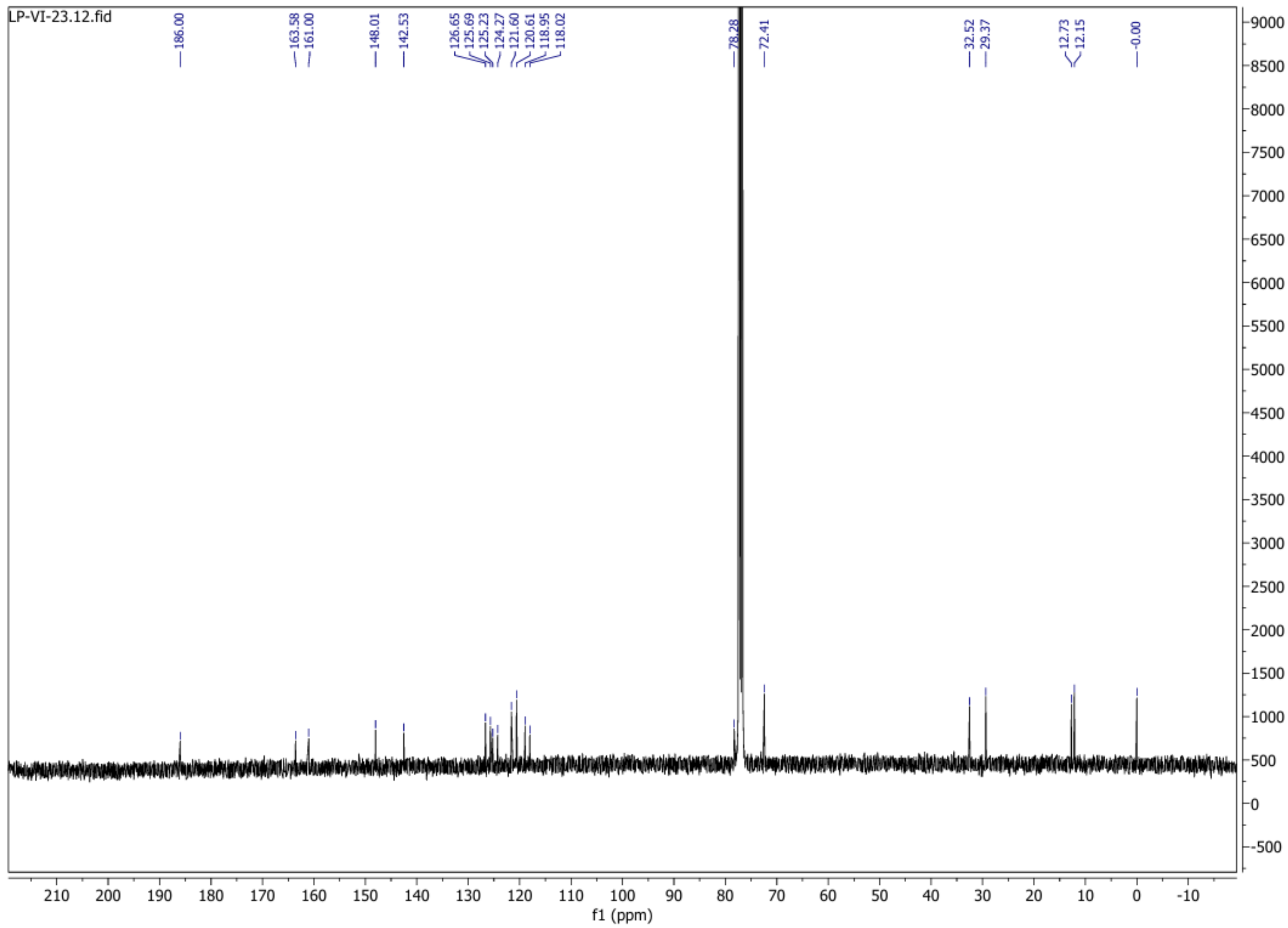

LP-VI-23.12.fid
186.00
163.58
161.00
148.01
142.53
126.65
125.69
125.23
124.27
121.60
120.61
118.95
118.02
78.28
72.41
32.52
29.37
12.73
12.15
-0.00
f1 (ppm)

NMR of compound 13

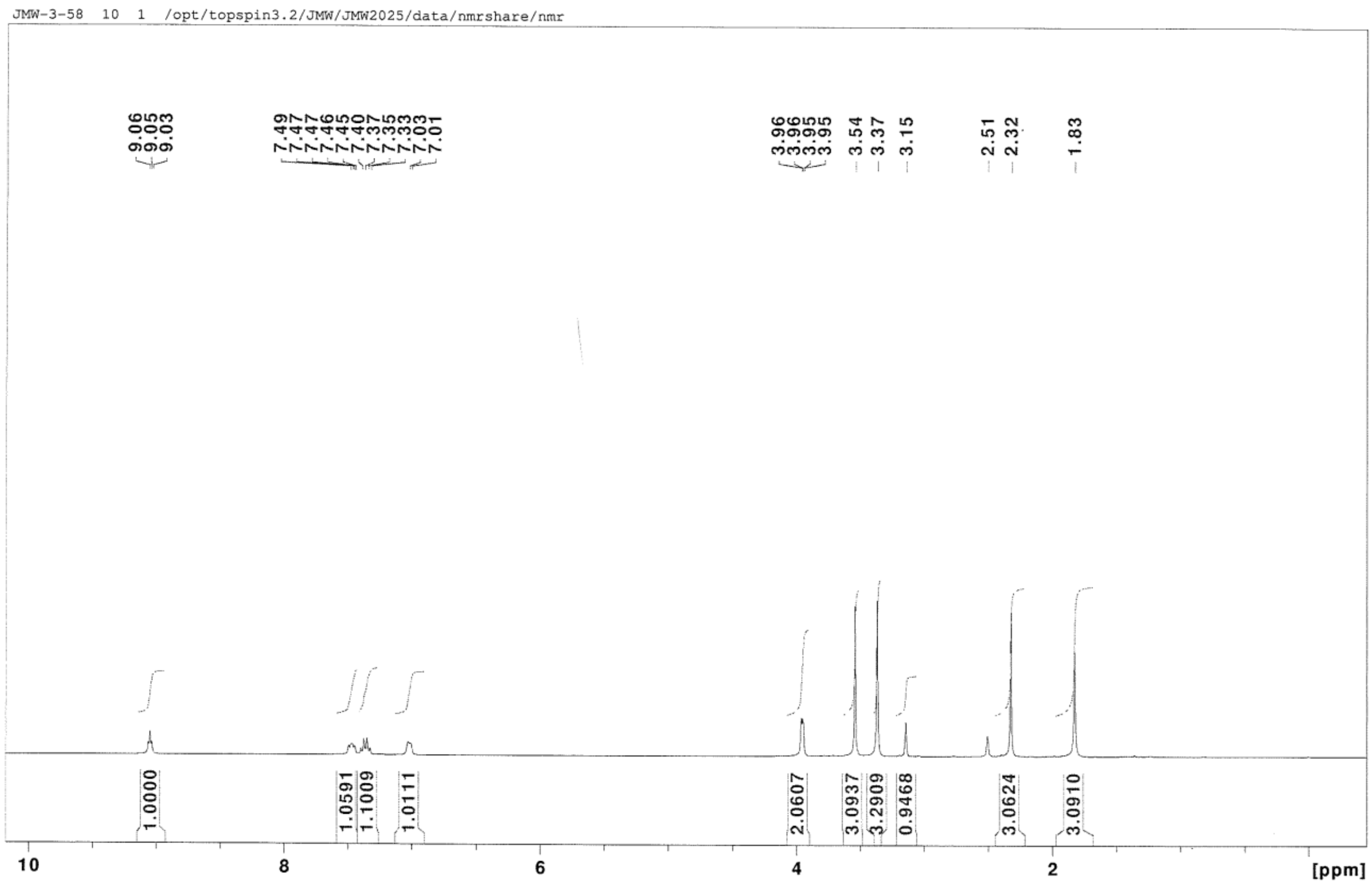

JMW-3-58 11 1 /opt/topspin3.2/JMW/JMW2025/data/nmrshare/nmr

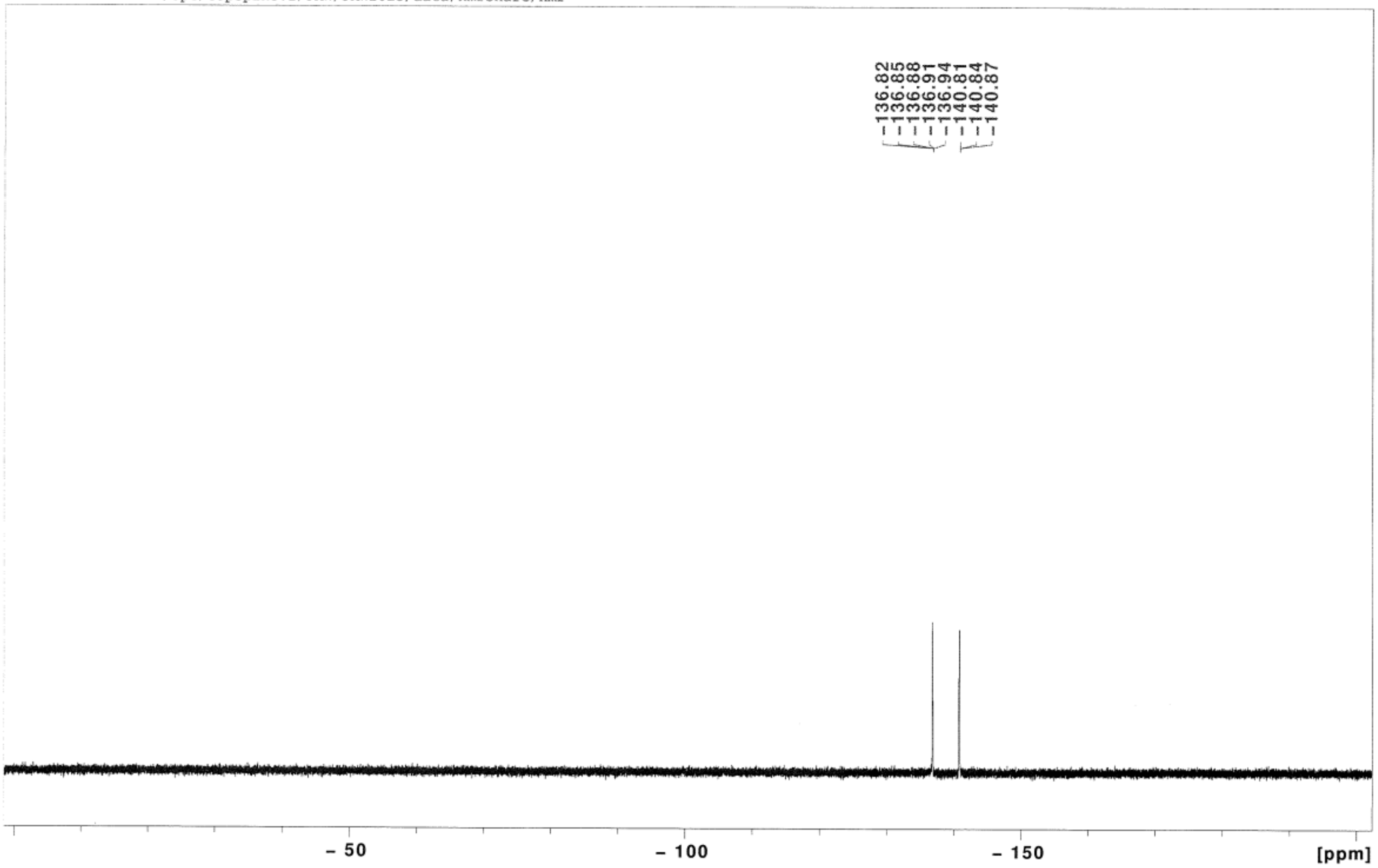

JMW-3-58 16 1 /opt/topspin3.2/JMW/JMW2025/data/nmrshare/nmr

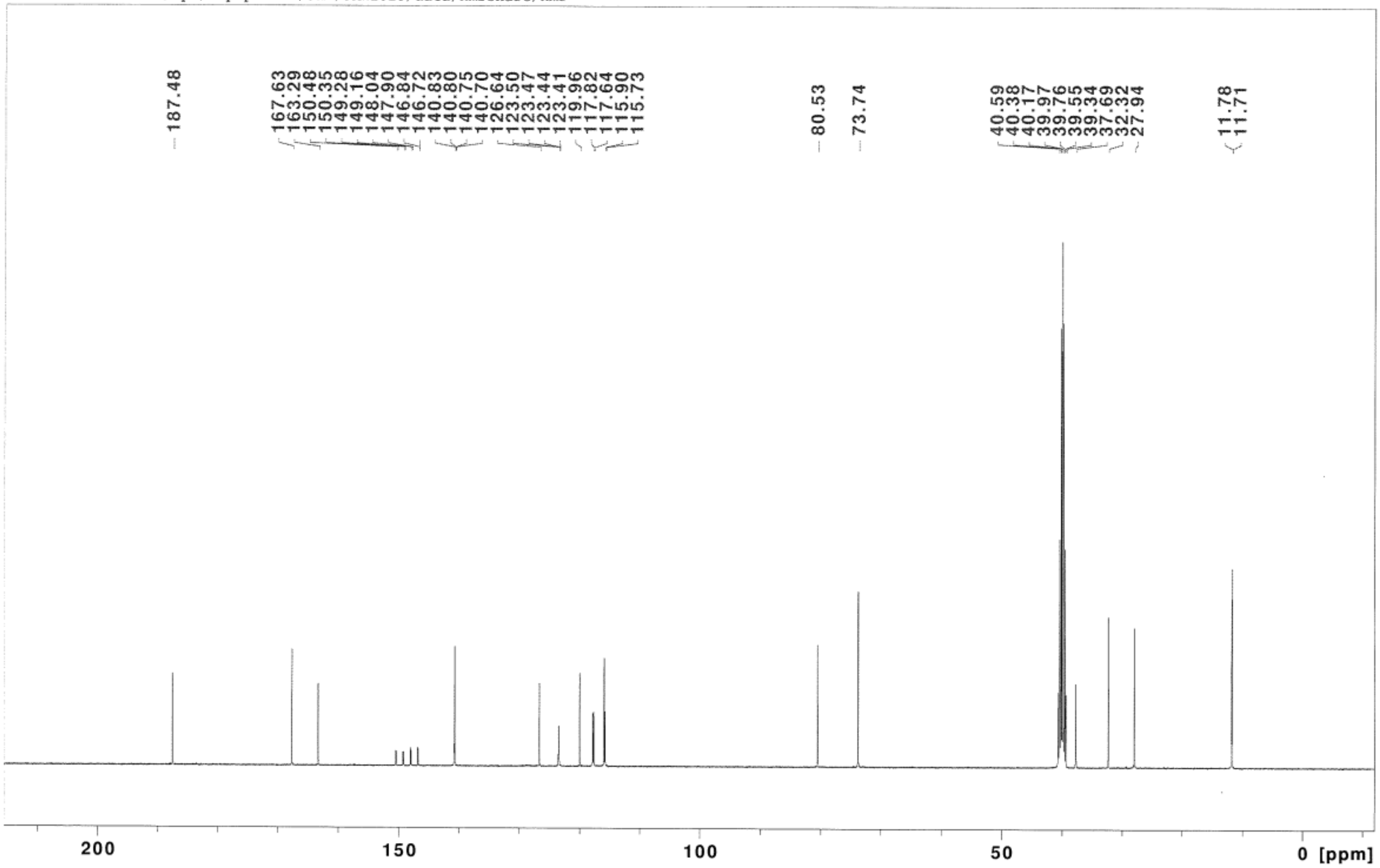

NMR of compound 16a

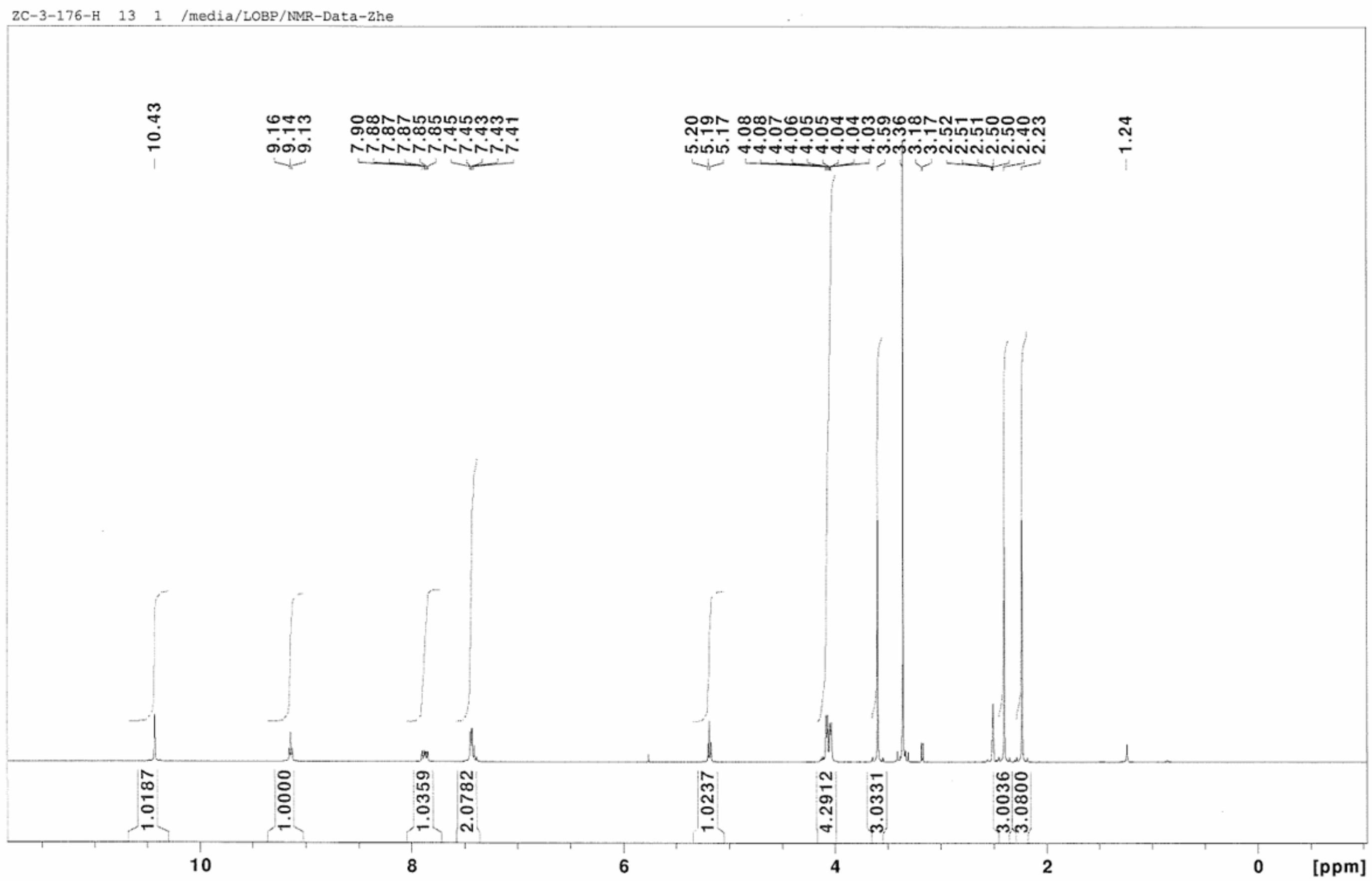

ZC-3-176-C 10 1 /opt/topspin3.2/ZC/ZC3/data/nmr00/nmr

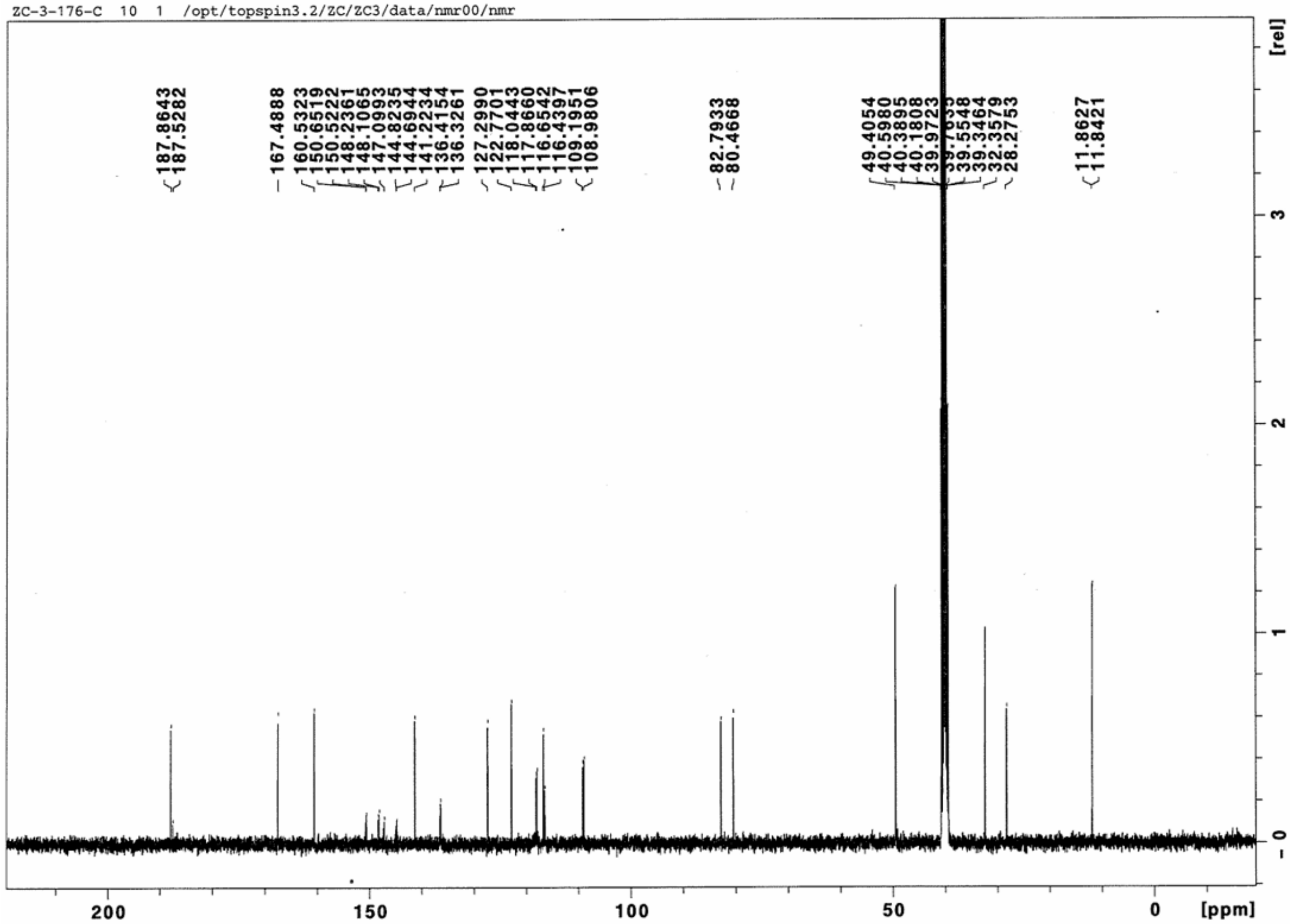

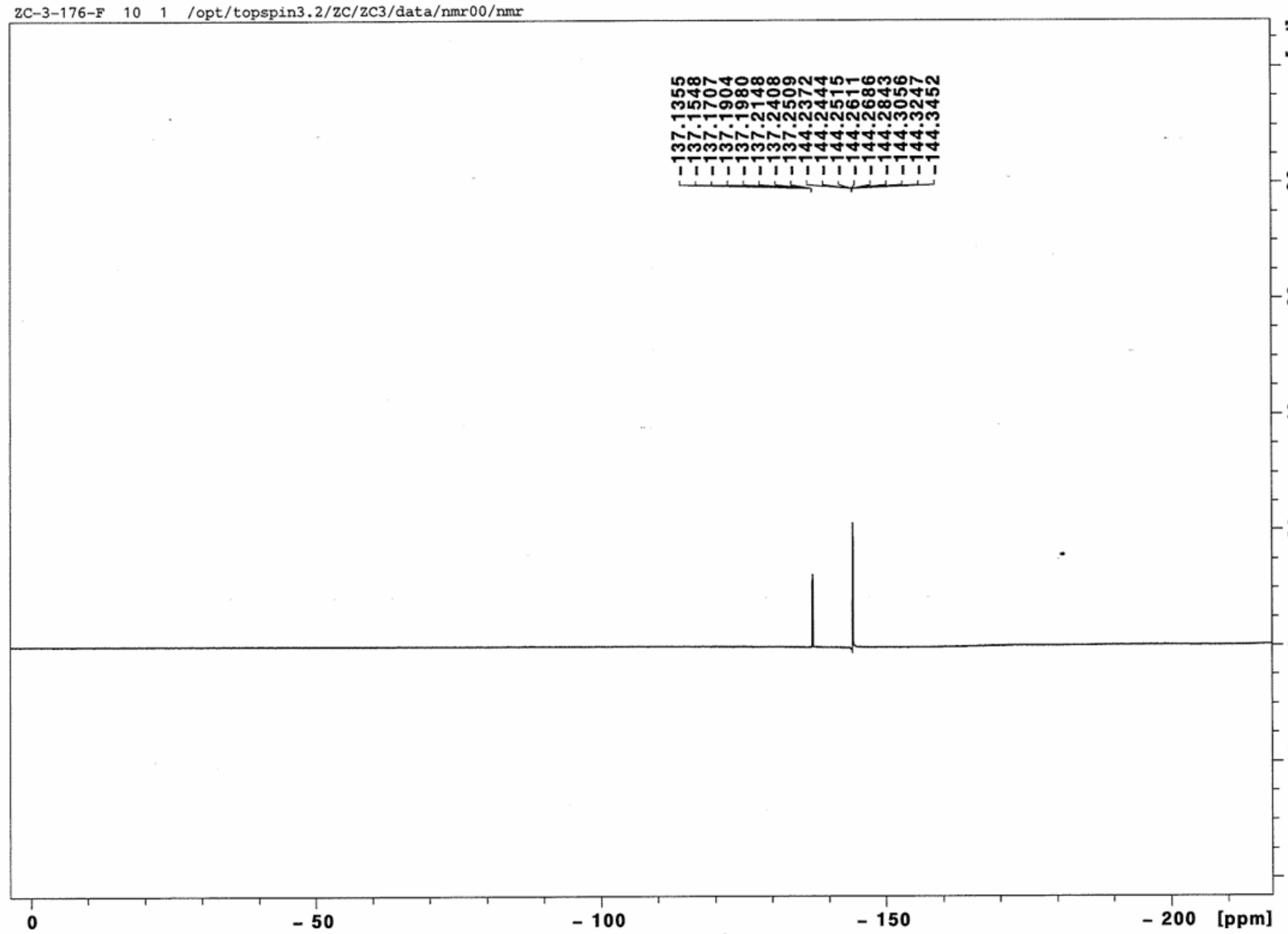
ZC-3-176-F 10 1 /opt/topspin3.2/ZC/ZC3/data/nmr00/nmr
-137.1355
-137.1548
-137.1707
-137.1904
-137.1980
-137.2148
-137.2408
-137.2509
-144.2372
-144.2444
-144.2515
-144.2611
-144.2686
-144.2843
-144.3056
-144.3247
-144.3452
[rel]
80
60
40
20
0
- 20
0
- 50
- 100
- 150
- 200
[ppm]

NMR of compound 16b

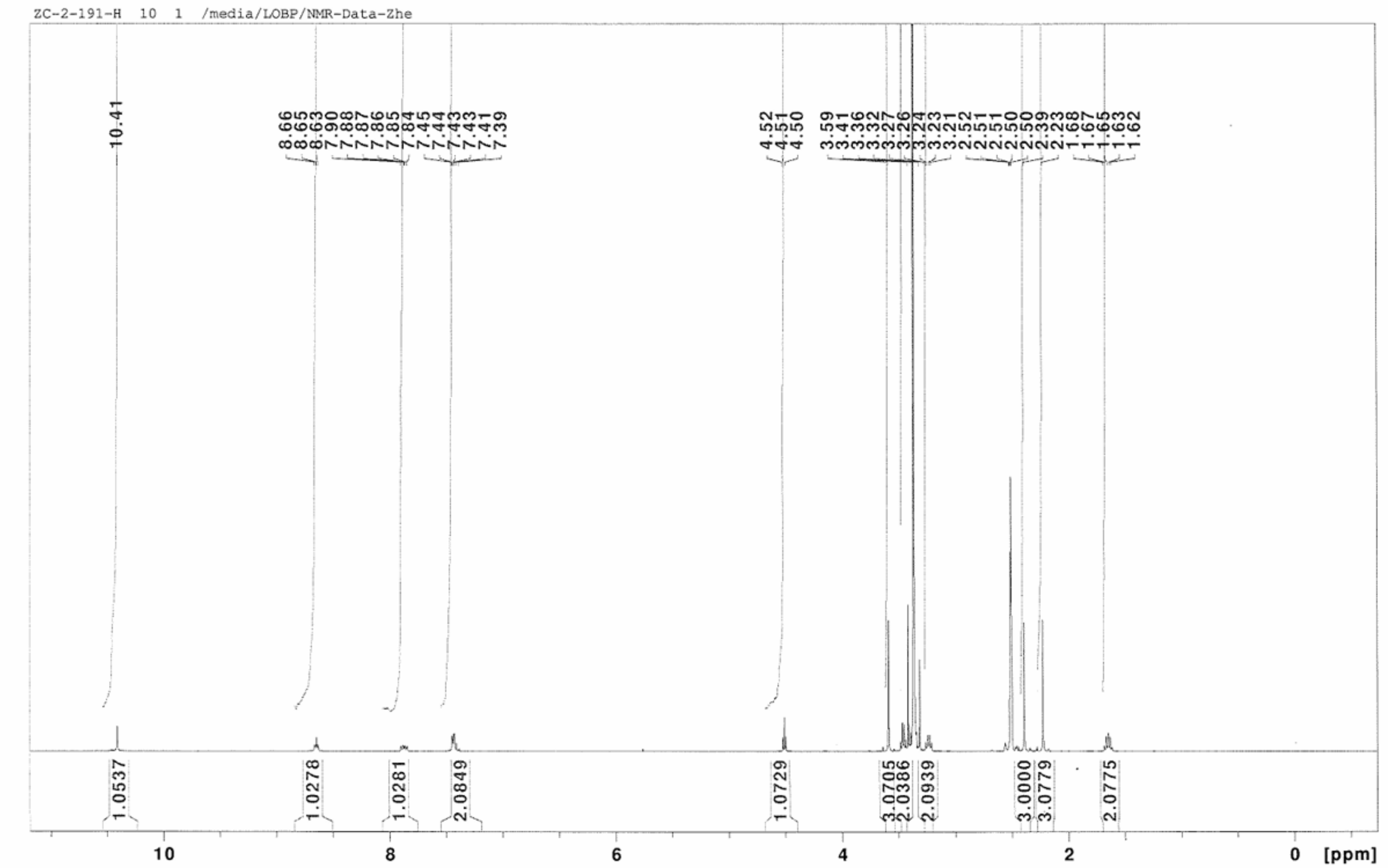

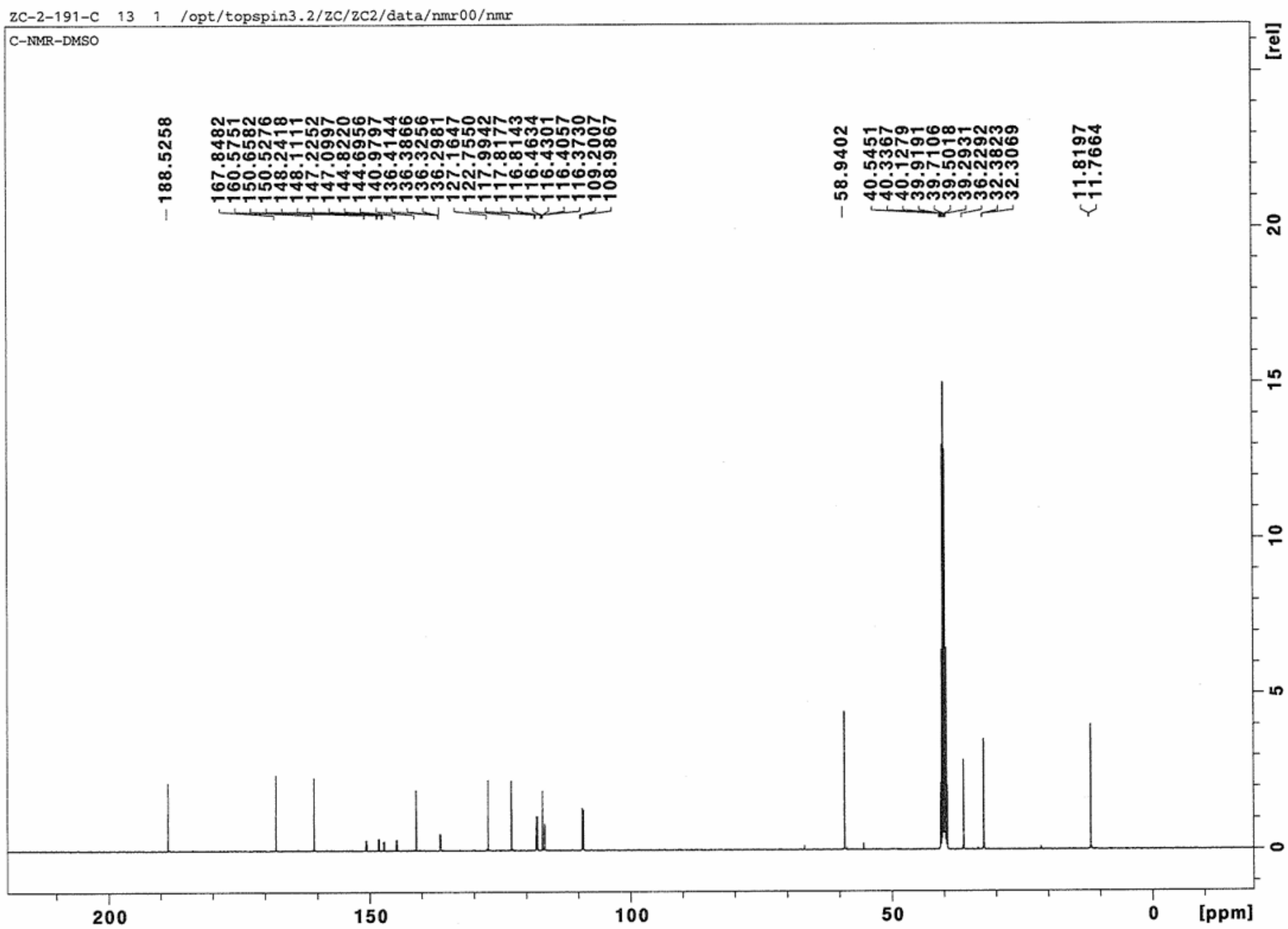
ZC-2-191-C 13 1 /opt/topspin3.2/ZC/ZC2/data/nmr00/nmr
C-NMR-DMSO
188.5258
167.8482
160.5751
150.6582
150.5276
148.2418
148.1111
147.2252
147.0997
144.8220
144.6956
140.9797
136.4144
136.3866
136.3256
136.2981
127.1647
122.7550
117.9942
117.8177
116.8143
116.4634
116.4301
116.4057
116.3730
109.2007
108.9867
58.9402
40.5451
40.3367
40.1279
39.9191
39.7106
39.5018
39.2931
36.2292
32.3823
32.3069
11.8197
11.7664
[rel]
20
15
10
5
0
200
150
100
50
0
[ppm]

ZC-2-191-F 19 1 /opt/topspin3.2/ZC/ZC2/data/nmr00/nmr

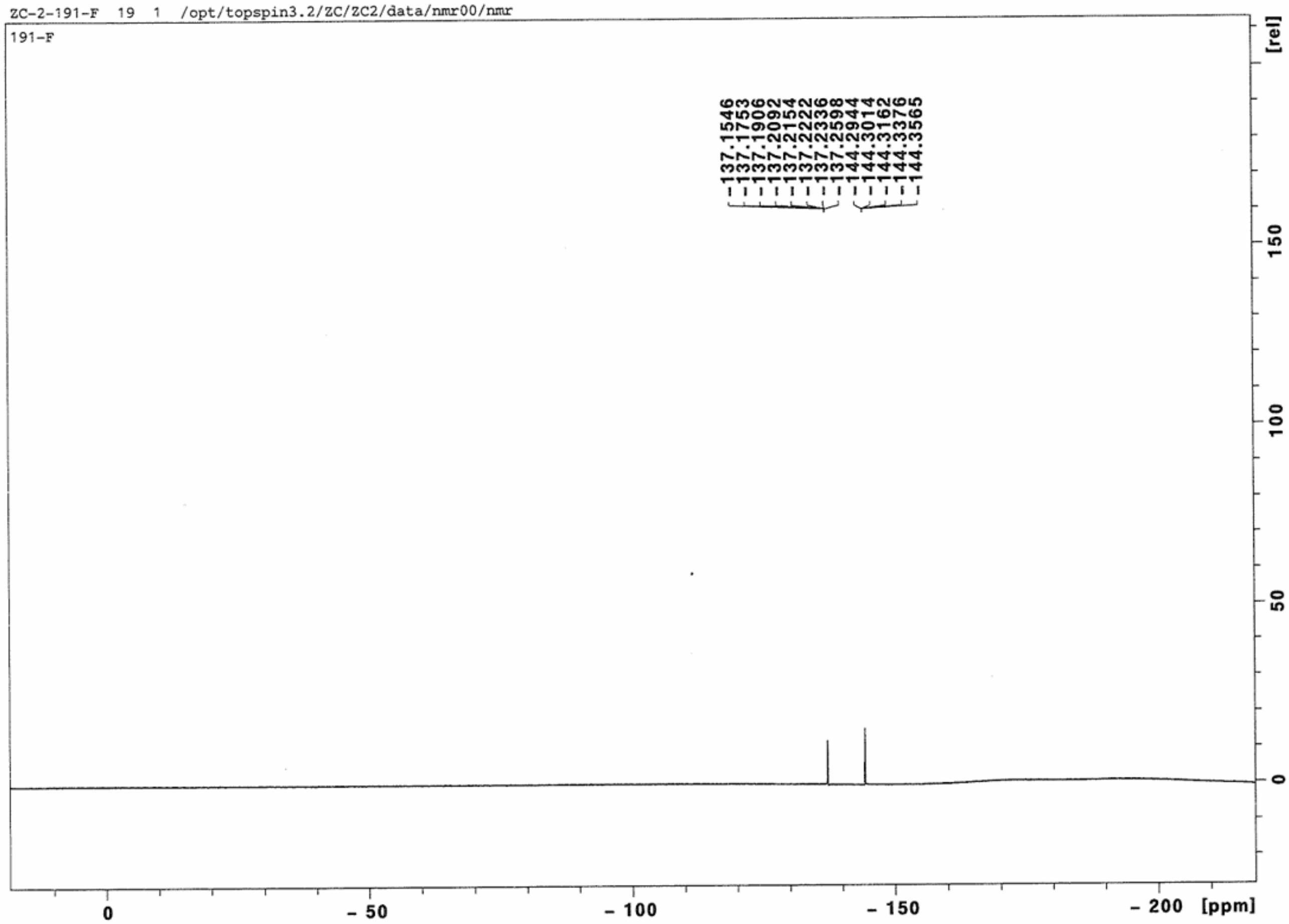

NMR of compound 16c

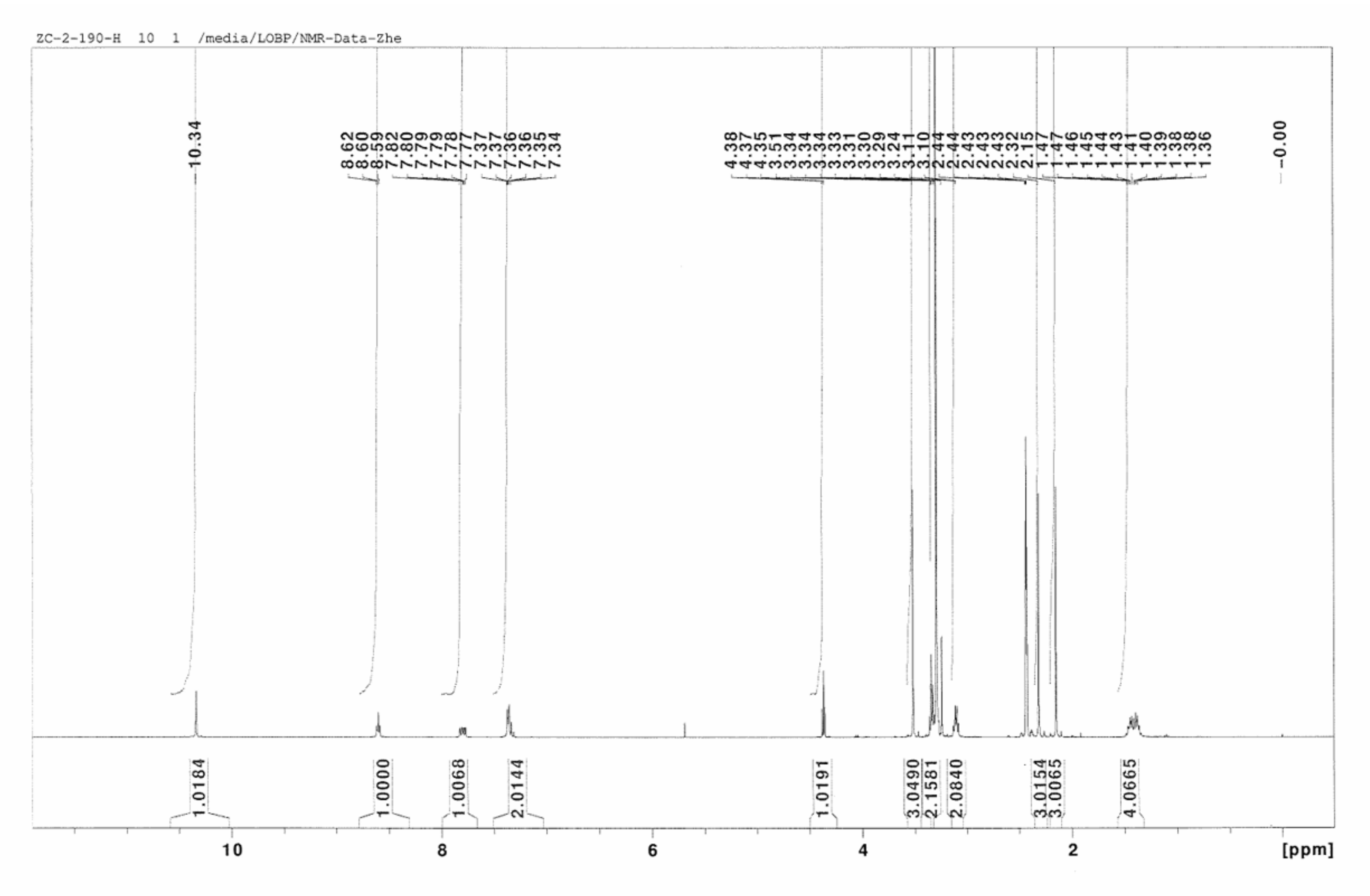

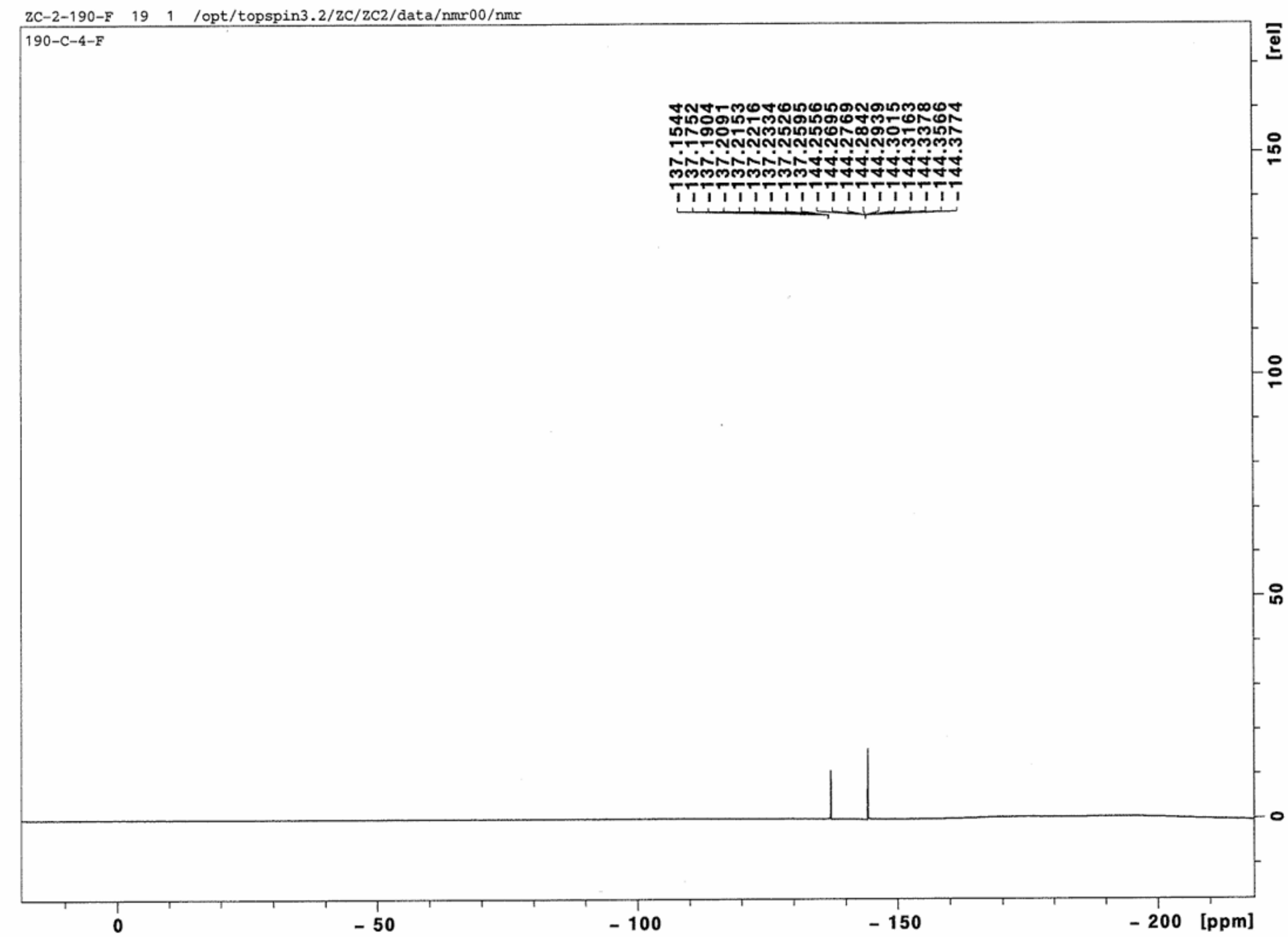
ZC-2-190-F 19 1 /opt/topspin3.2/ZC/ZC2/data/nmr00/nmr
190-C-4-F
-137.1544
-137.1752
-137.1904
-137.2091
-137.2153
-137.2216
-137.2334
-137.2526
-137.2595
-144.2556
-144.2695
-144.2769
-144.2842
-144.2939
-144.3015
-144.3163
-144.3378
-144.3566
-144.3774
[rel]
150
100
50
0
0
- 50
- 100
- 150
- 200
[ppm]

NMR of compound 16e

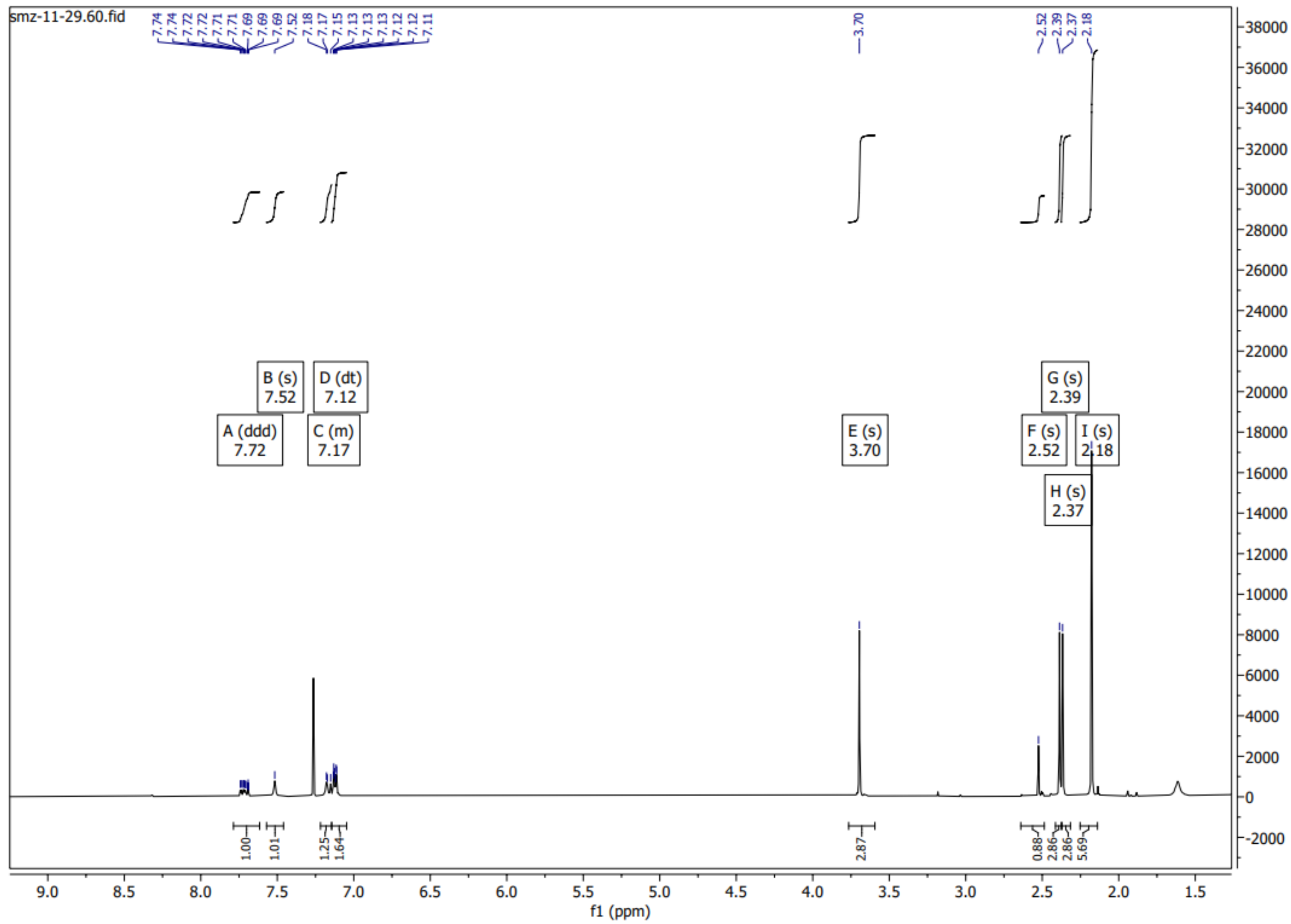

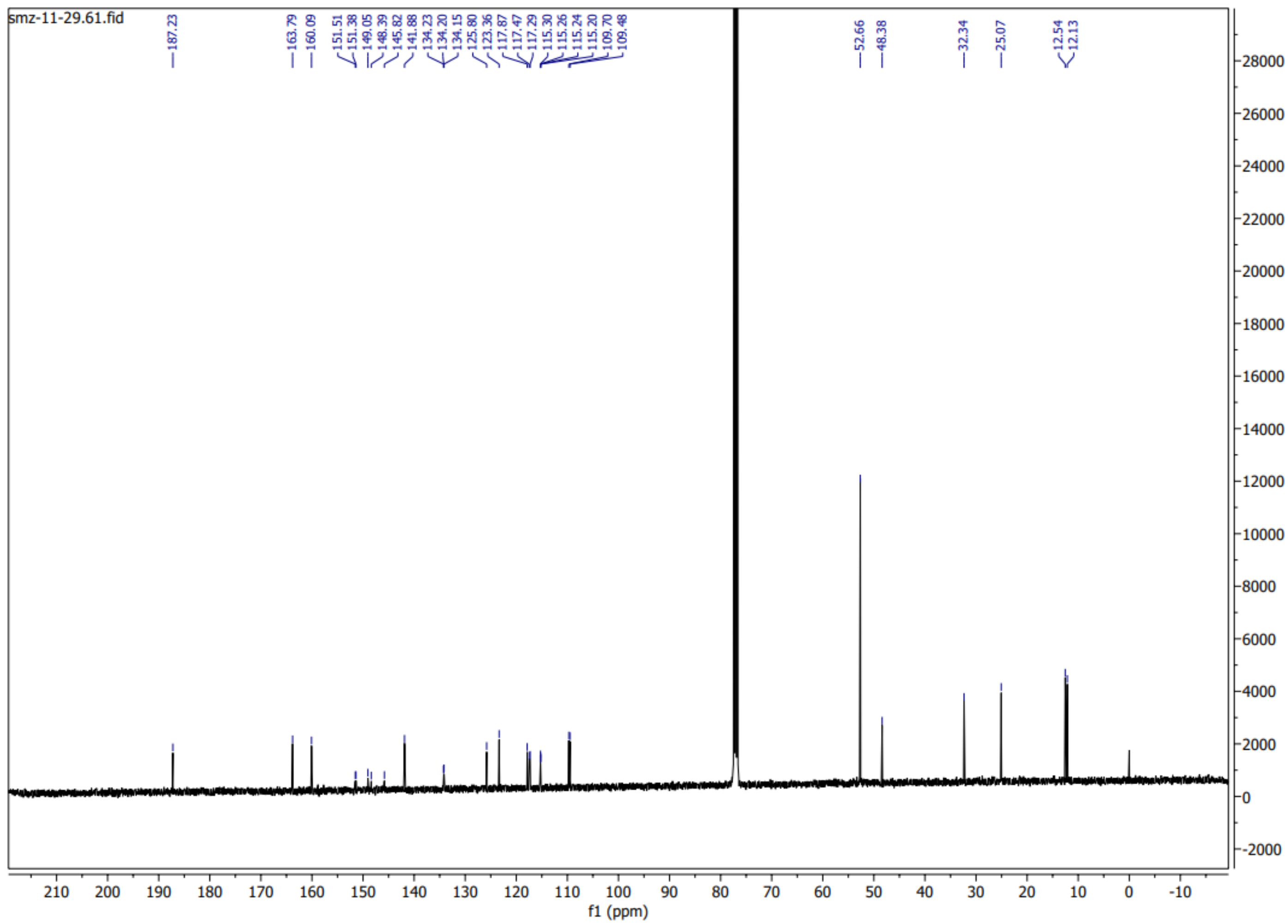
smz-11-29.61.fid
187.23
163.79
160.09
151.51
151.38
149.05
148.39
145.82
141.88
134.23
134.20
134.15
125.80
123.36
117.87
117.47
117.29
115.30
115.26
115.24
115.20
109.70
109.48
52.66
48.38
32.34
25.07
12.54
12.13
f1 (ppm)

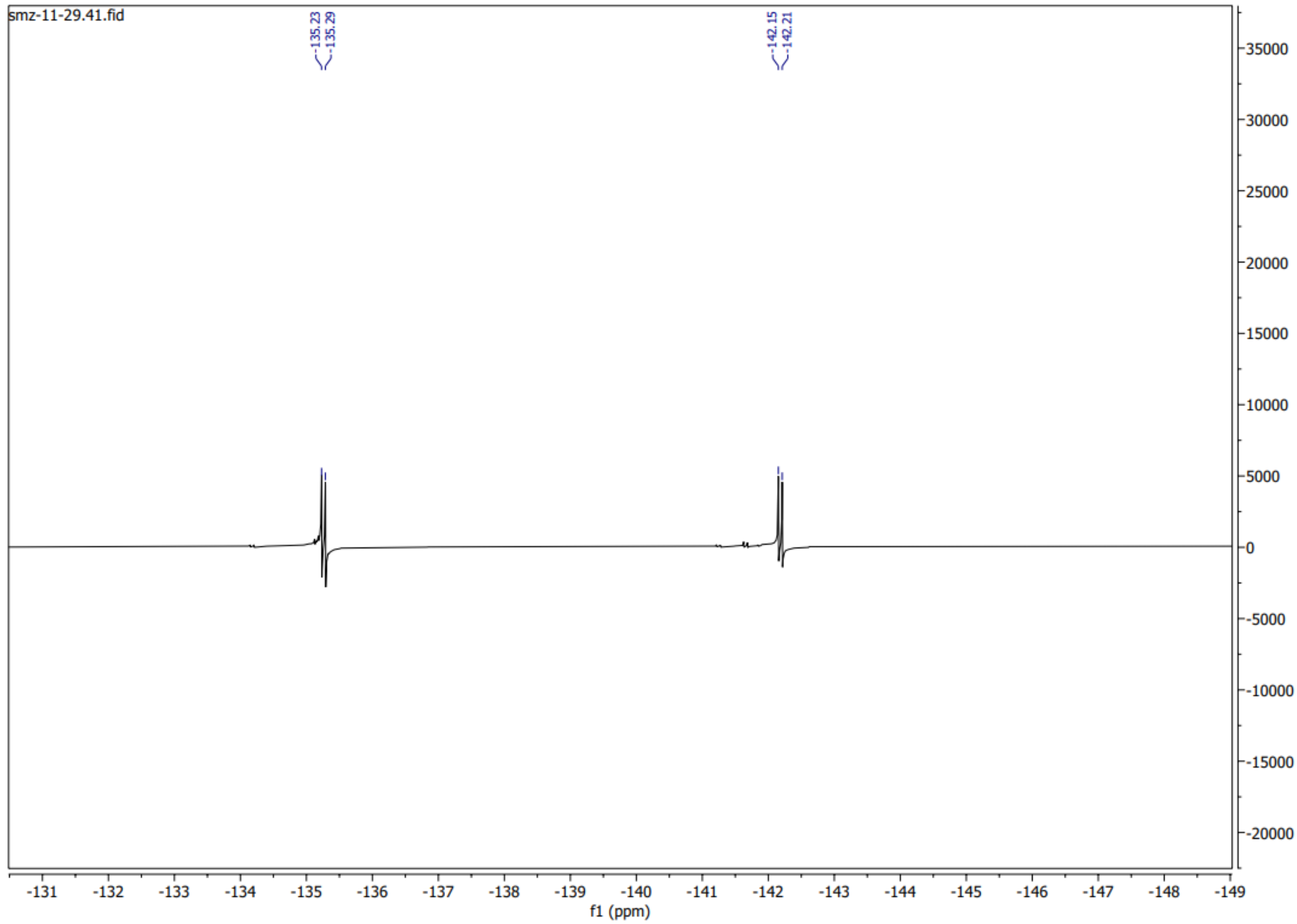
smz-11-29.41.fid
-135.23
-135.29
-142.15
-142.21
35000
30000
25000
20000
15000
10000
5000
0
-5000
-10000
-15000
-20000
-131 -132 -133 -134 -135 -136 -137 -138 -139 -140 -141 -142 -143 -144 -145 -146 -147 -148 -149
f1 (ppm)

NMR of compound 16f

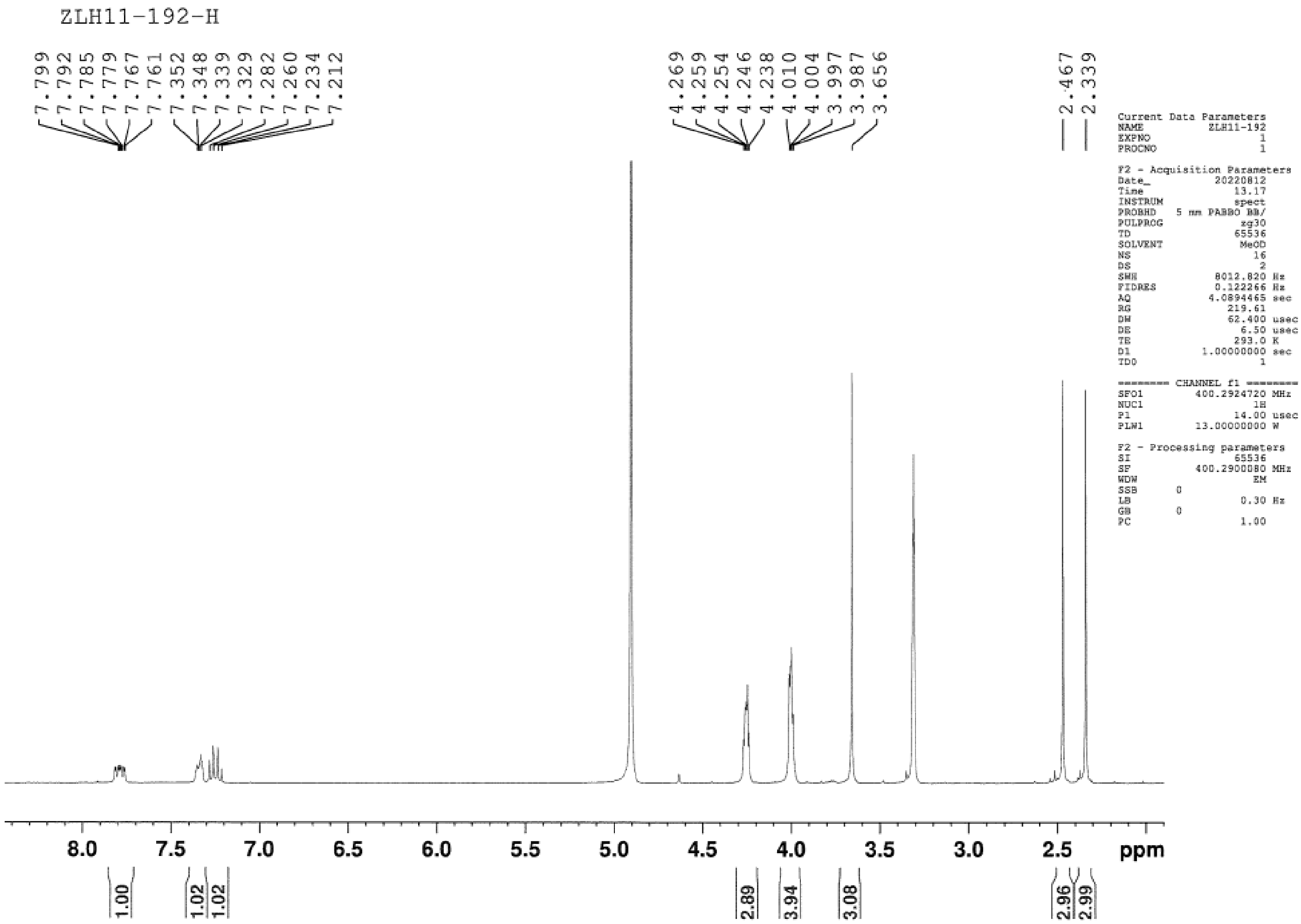

ZLH11-192-C

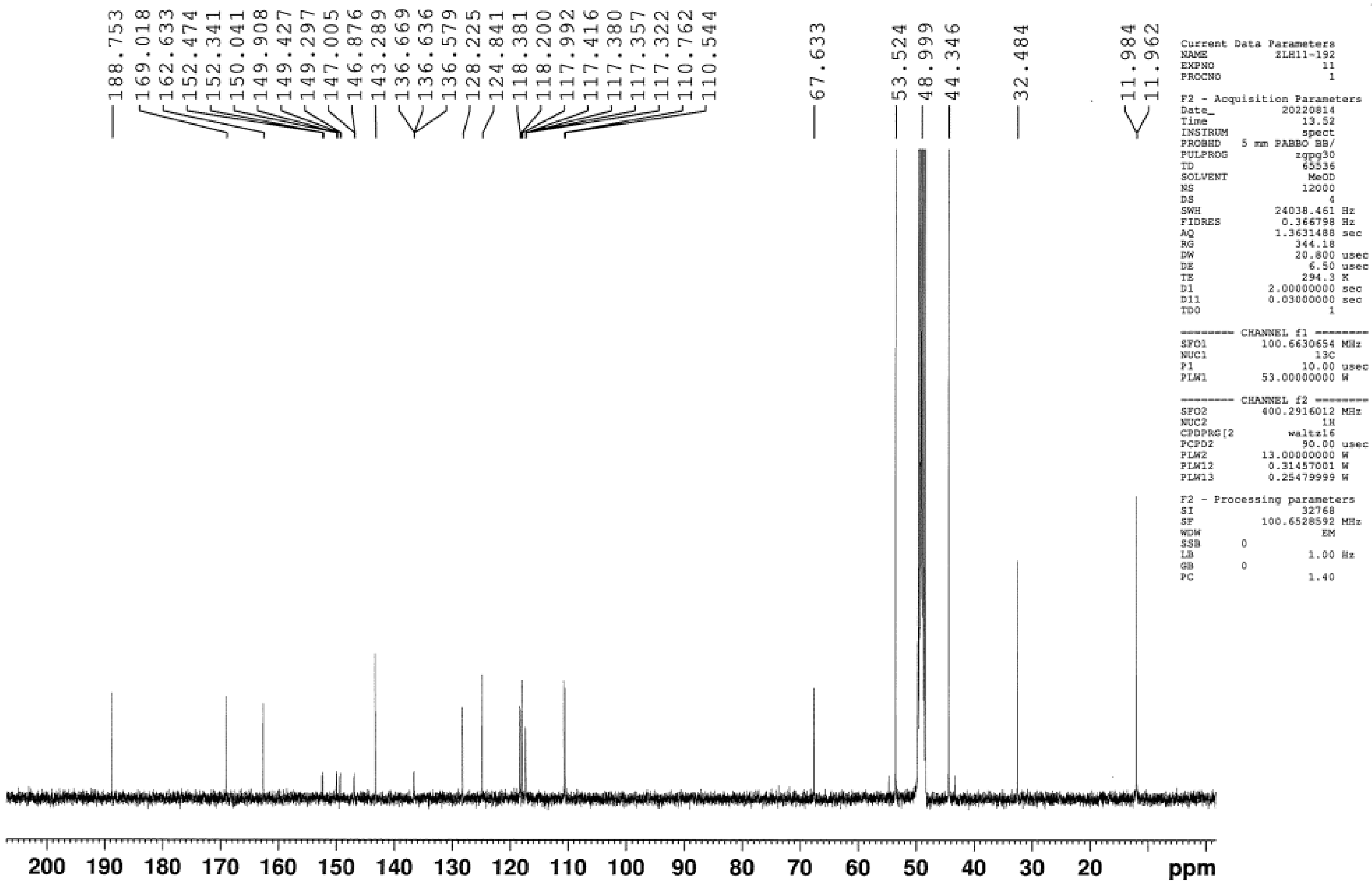
188.753
169.018
162.633
152.474
152.341
150.041
149.908
149.427
149.297
147.005
146.876
143.289
136.669
136.636
136.579
128.225
124.841
118.381
118.200
117.992
117.416
117.380
117.357
117.322
110.762
110.544
67.633
53.524
48.999
44.346
32.484
11.984
11.962
Current Data Parameters
NAME ZLH11-192
EXPNO 11
PROCNO 1
F2 - Acquisition Parameters
Date_ 20220814
Time 13.52
INSTRUM spect
PROBHD 5 mm PABBO BB/
PULPROG zgpg30
TD 65536
SOLVENT MeOD
NS 12000
DS 4
SWH 24038.461 Hz
FIDRES 0.366798 Hz
AQ 1.3631488 sec
RG 344.18
DW 20.800 usec
DE 6.50 usec
TE 294.3 K
D1 2.00000000 sec
D11 0.03000000 sec
TD0 1
======== CHANNEL f1 ========
SFO1 100.6630654 MHz
NUC1 13C
P1 10.00 usec
PLW1 53.00000000 W
======== CHANNEL f2 ========
SFO2 400.2916012 MHz
NUC2 1H
CPDPRG[2 waltz16
PCPD2 90.00 usec
PLW2 13.00000000 W
PLW12 0.31457001 W
PLW13 0.25479999 W
F2 - Processing parameters
SI 32768
SF 100.6528592 MHz
WDW EM
SSB 0
LB 1.00 Hz
GB 0
PC 1.40
200 190 180 170 160 150 140 130 120 110 100 90 80 70 60 50 40 30 20 ppm

ZLH11-192-F

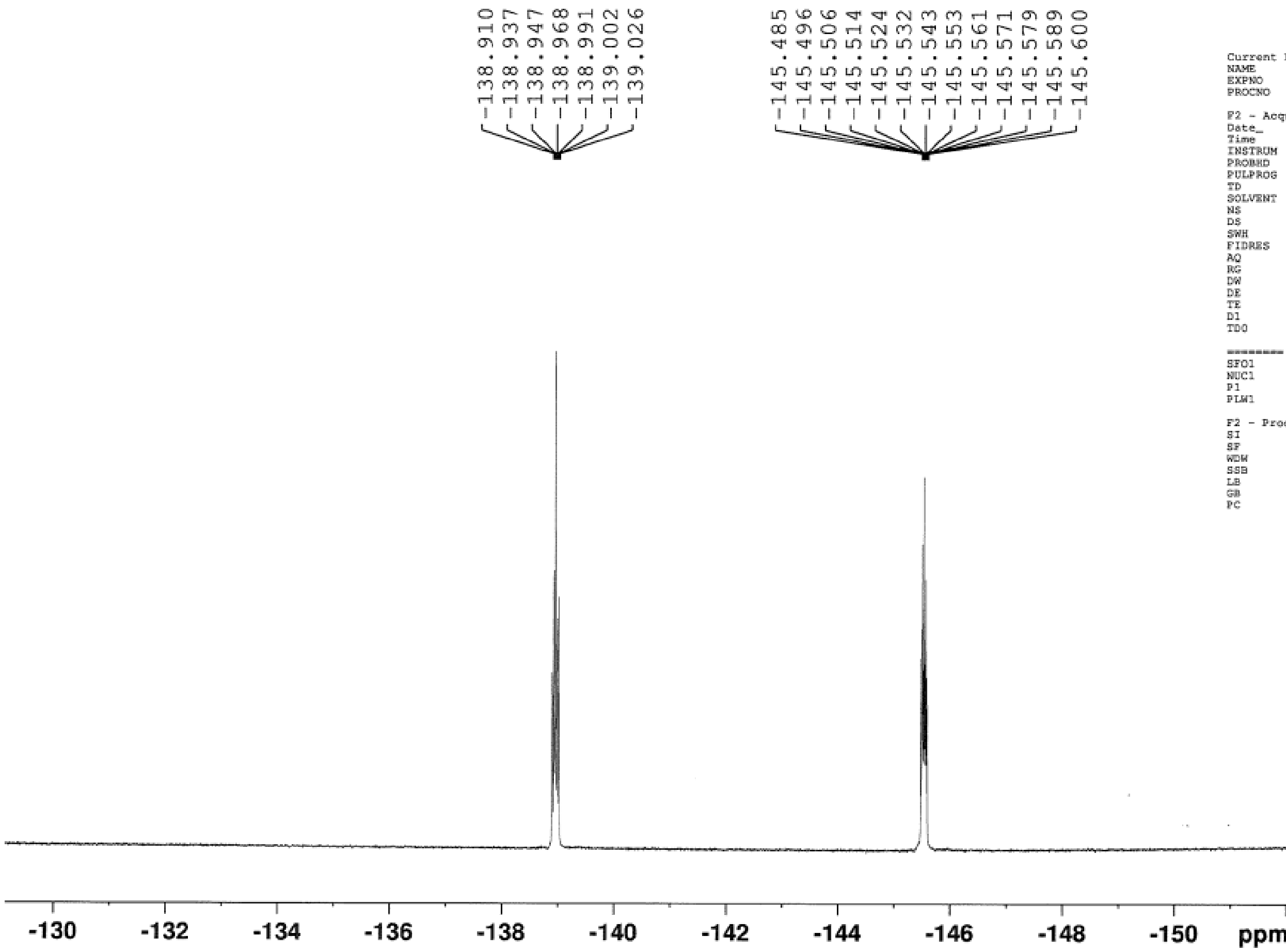


```
Current Data Parameters
NAME             ZLH11-192
EXPNO                   21
PROCNO                   1

F2 - Acquisition Parameters
Date_             20220815
Time                  8.55
INSTRUM              spect
PROBHD    5 mm PABBO BB/
PULPROG             zgflqn
TD                  131072
SOLVENT               MeOD
NS                     128
DS                       4
SWH              89285.711 Hz
FIDRES            0.681196 Hz
AQ               0.7340032 sec
RG                  548.76
DW                   5.600 usec
DE                    6.50 usec
TE                   293.7 K
D1              1.00000000 sec
TDO                      1

======== CHANNEL f1 ========
SFO1           376.6112517 MHz
NUC1                   19F
P1                   15.00 usec
PLW1           17.00000000 W

F2 - Processing parameters
SI                   65536
SF             376.6489170 MHz
WDW                     EM
SSB      0
LB                    0.30 Hz
GB       0
PC                    1.00
```

NMR of compound 16g

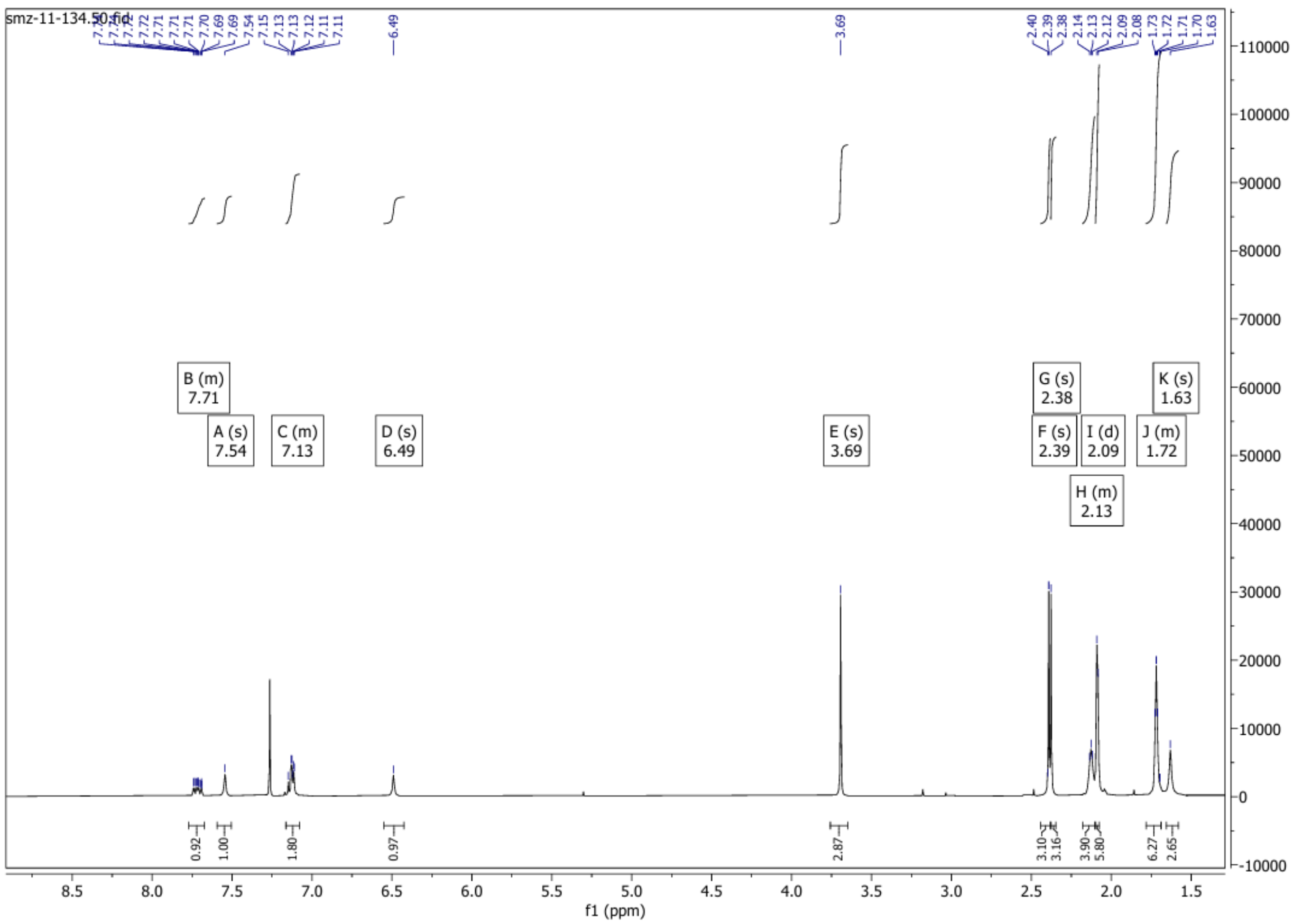

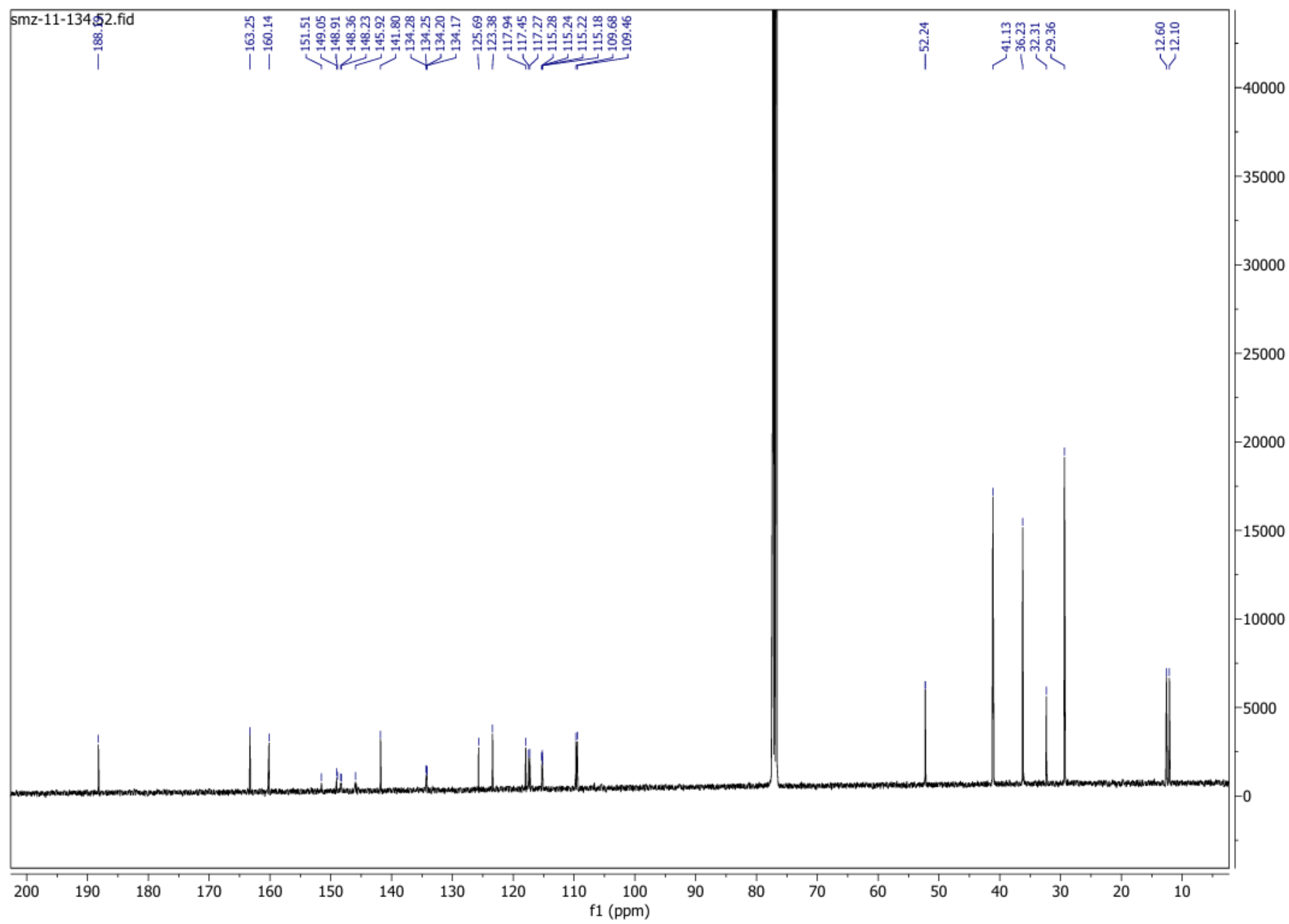
smz-11-134.52.fid
163.25
160.14
151.51
149.05
148.91
148.36
148.23
145.92
141.80
134.28
134.25
134.20
134.17
125.69
123.38
117.94
117.45
117.27
115.28
115.24
115.22
115.18
109.68
109.46
52.24
41.13
36.23
32.31
29.36
12.60
12.10
40000
35000
30000
25000
20000
15000
10000
5000
0
200 190 180 170 160 150 140 130 120 110 100 90 80 70 60 50 40 30 20 10
f1 (ppm)

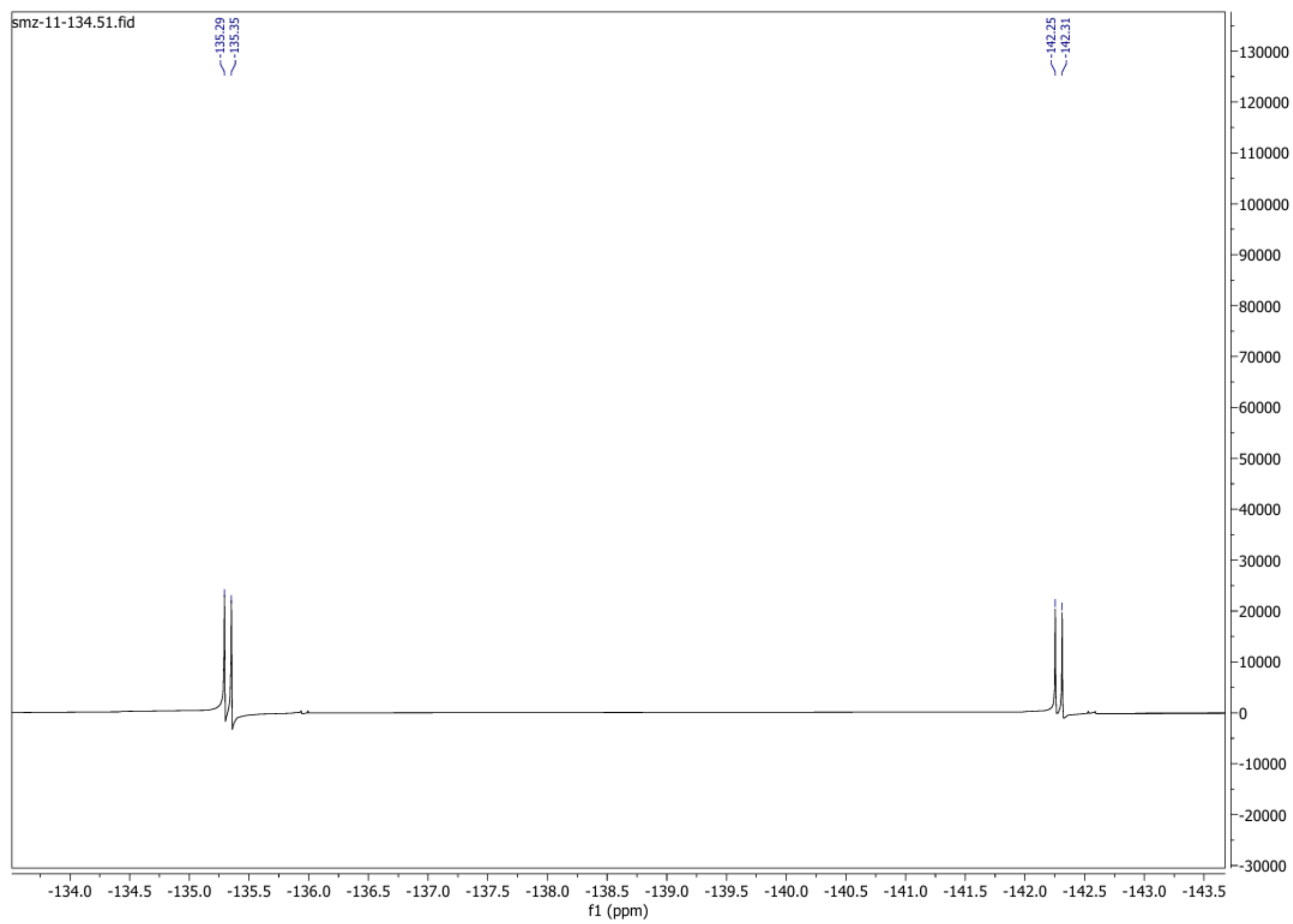
smz-11-134.51.fid
-135.29
-135.35
-142.25
-142.31
130000
120000
110000
100000
90000
80000
70000
60000
50000
40000
30000
20000
10000
0
-10000
-20000
-30000
-134.0 -134.5 -135.0 -135.5 -136.0 -136.5 -137.0 -137.5 -138.0 -138.5 -139.0 -139.5 -140.0 -140.5 -141.0 -141.5 -142.0 -142.5 -143.0 -143.5
f1 (ppm)

NMR of compound 16h

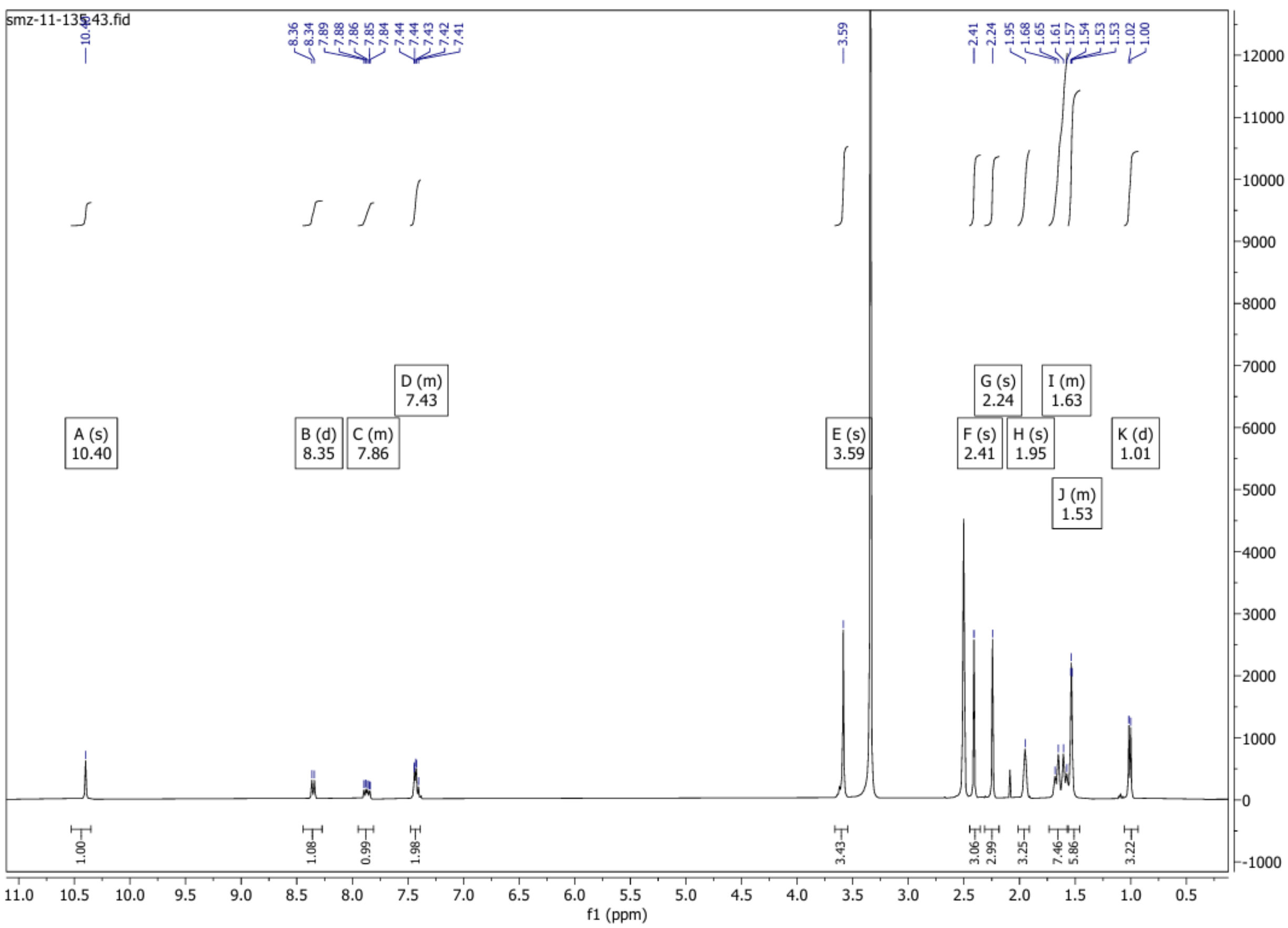

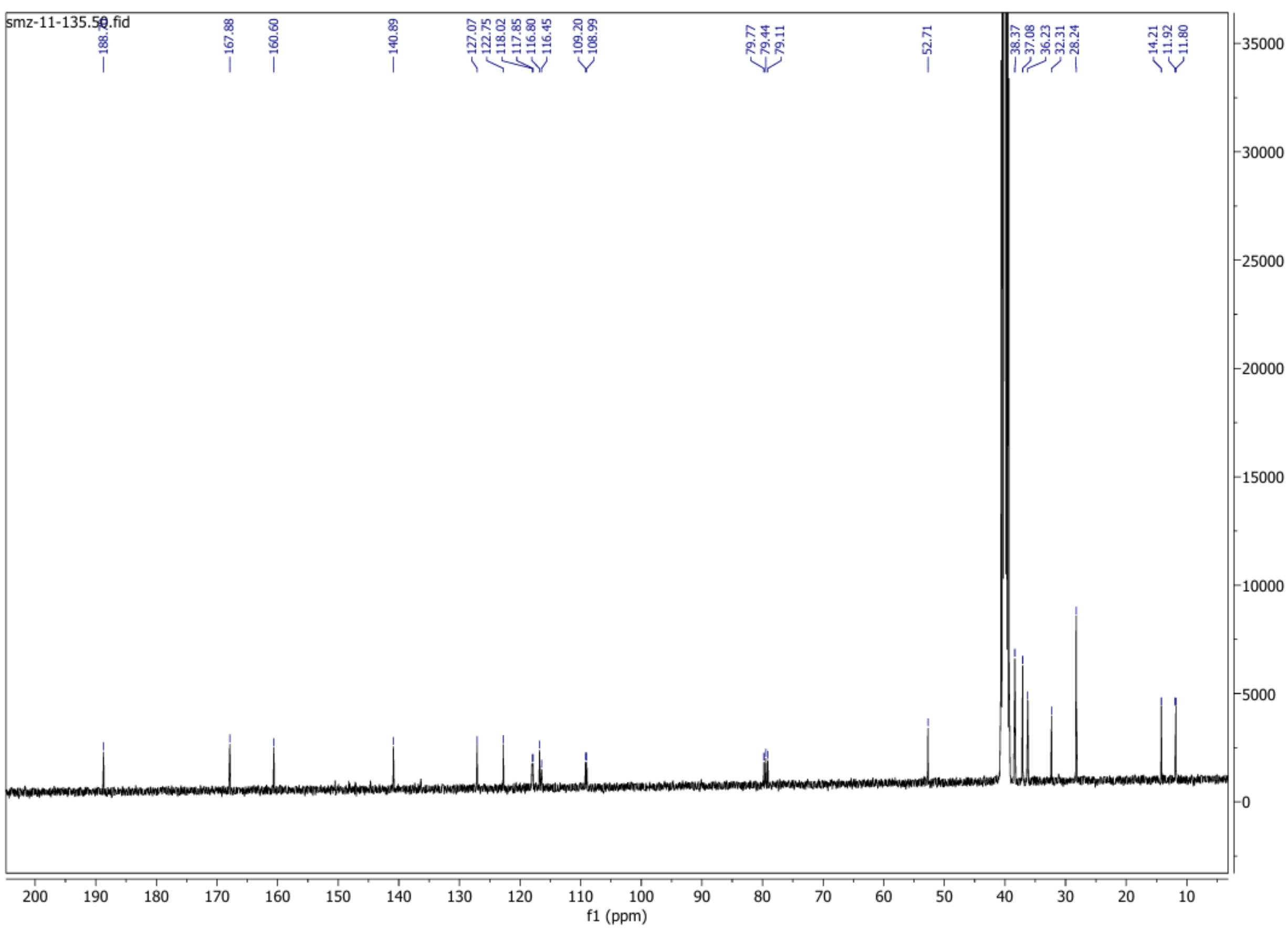
smz-11-135.50.fid
188.30
167.88
160.60
140.89
127.07
122.75
118.02
117.85
116.80
116.45
109.20
108.99
79.77
79.44
79.11
52.71
38.37
37.08
36.23
32.31
28.24
14.21
11.92
11.80
35000
30000
25000
20000
15000
10000
5000
0
200
190
180
170
160
150
140
130
120
110
100
90
80
70
60
50
40
30
20
10
f1 (ppm)

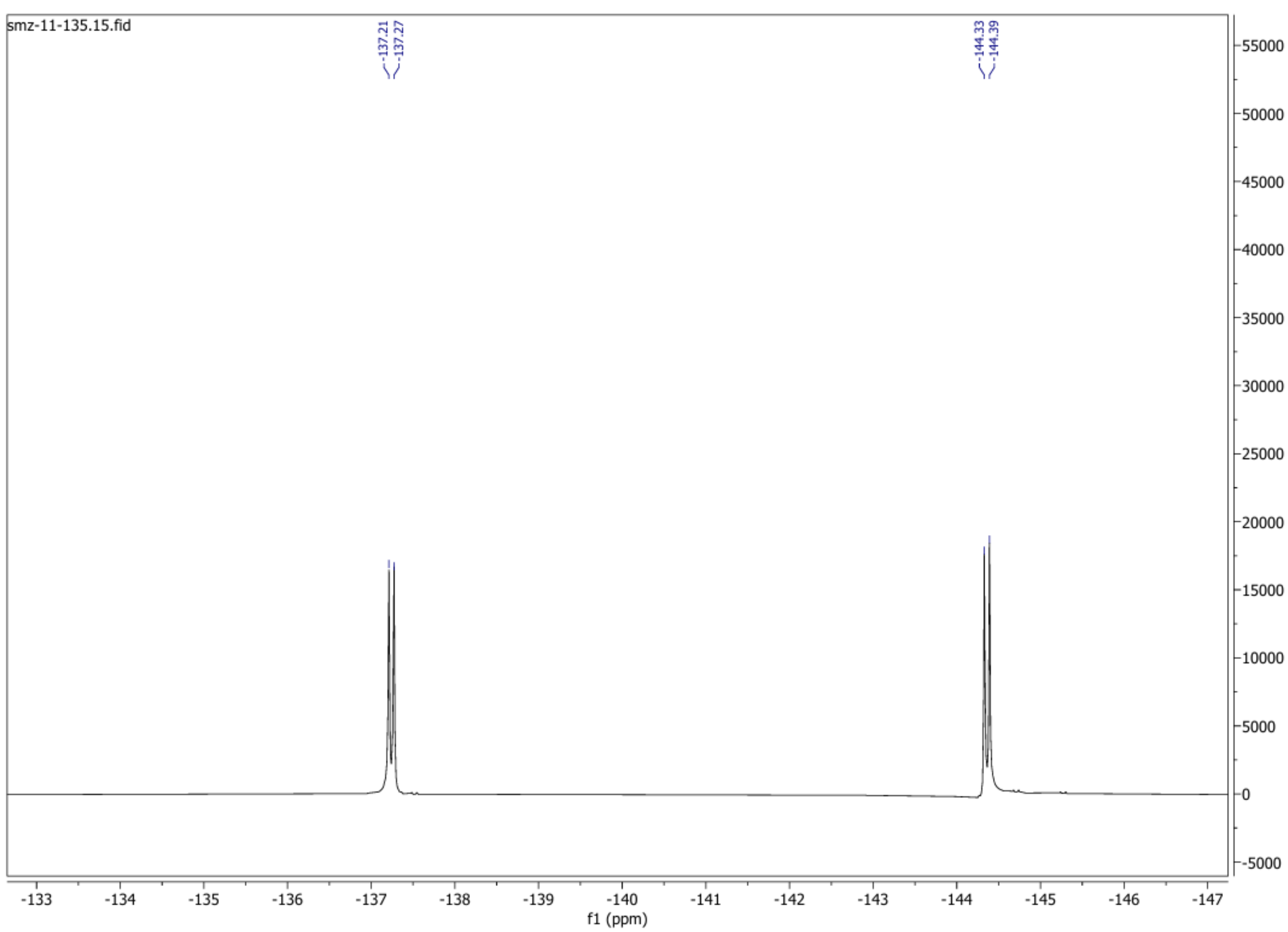
smz-11-135.15.fid
-137.21
-137.27
-144.33
-144.39
55000
50000
45000
40000
35000
30000
25000
20000
15000
10000
5000
0
-5000
-133
-134
-135
-136
-137
-138
-139
-140
-141
-142
-143
-144
-145
-146
-147
f1 (ppm)

NMR of compound 17

ZC-3-67-H-1 10 1 /media/LOBP/NMR-Data-Zhe

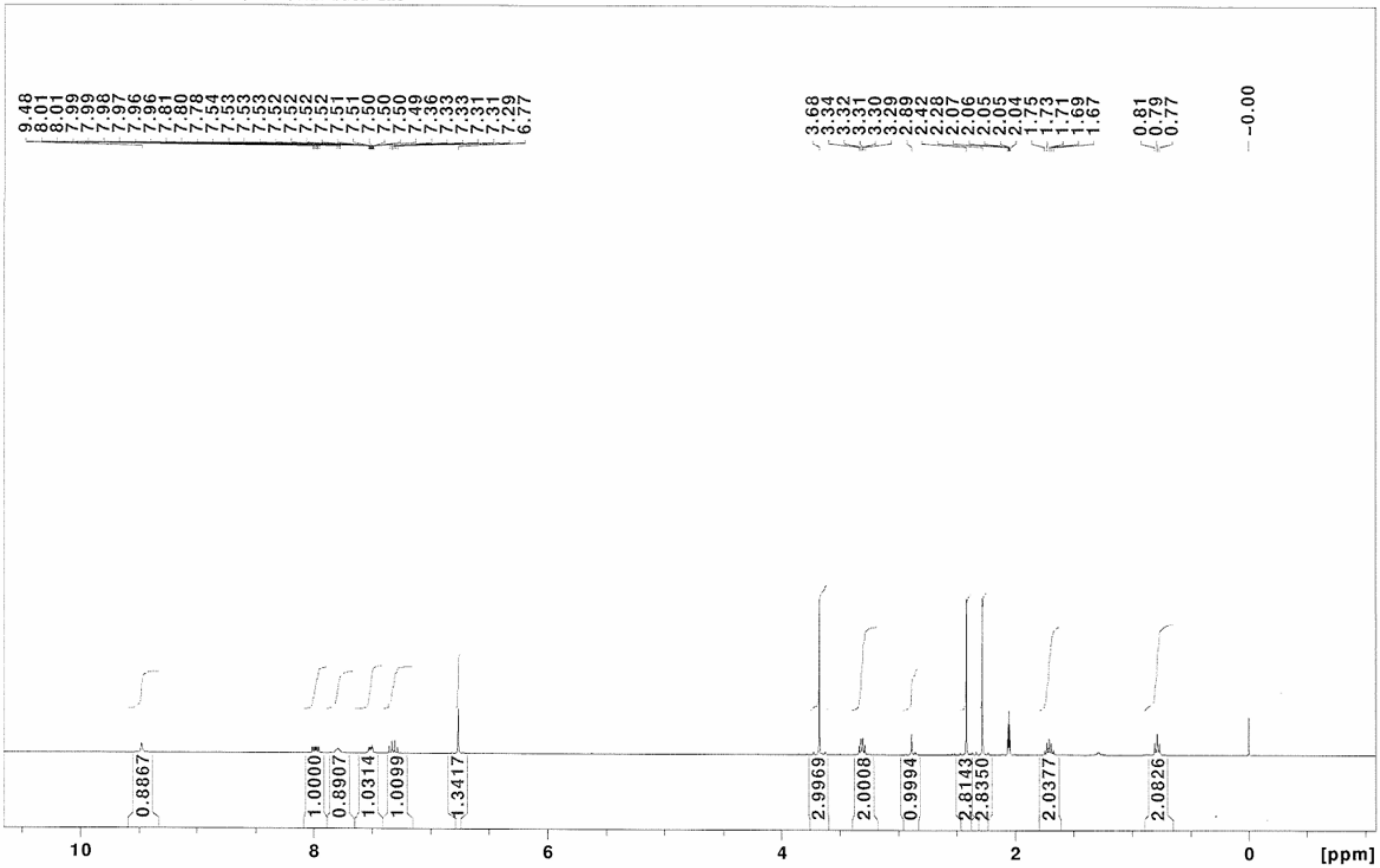

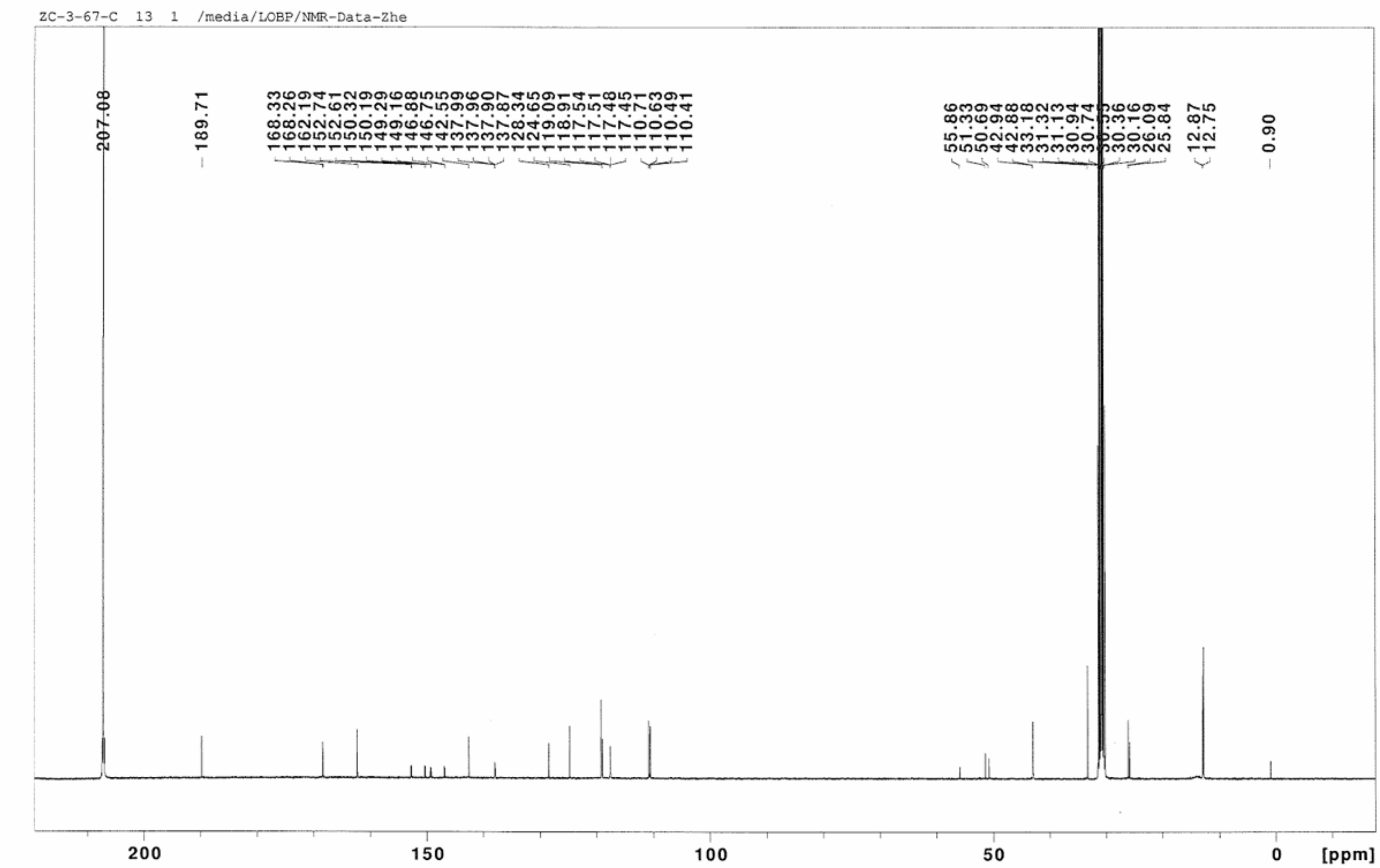
ZC-3-67-C 13 1 /media/LOBP/NMR-Data-Zhe
207.08
189.71
168.33
168.26
162.19
152.74
152.61
150.32
150.19
149.29
149.16
146.88
146.75
142.55
137.99
137.96
137.90
137.87
128.34
124.65
119.09
118.91
117.54
117.51
117.48
117.45
110.71
110.63
110.49
110.41
55.86
51.33
50.69
42.94
42.88
33.18
31.32
31.13
30.94
30.74
30.55
30.36
30.16
26.09
25.84
12.87
12.75
0.90
200
150
100
50
0
[ppm]

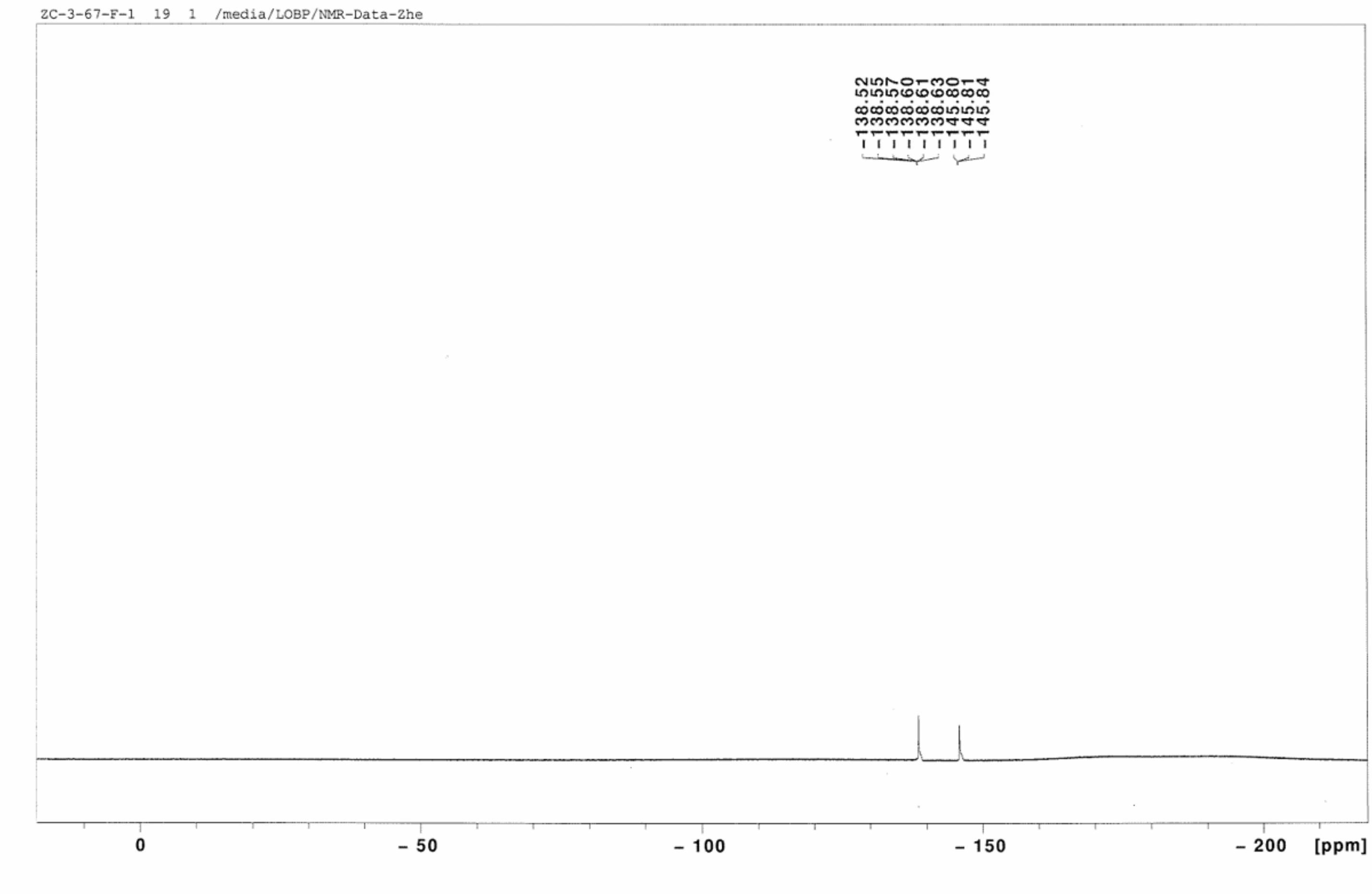
ZC-3-67-F-1 19 1 /media/LOBP/NMR-Data-Zhe
-138.52
-138.55
-138.57
-138.60
-138.61
-138.63
-145.80
-145.81
-145.84
0
- 50
- 100
- 150
- 200
[ppm]

NMR of compound 18

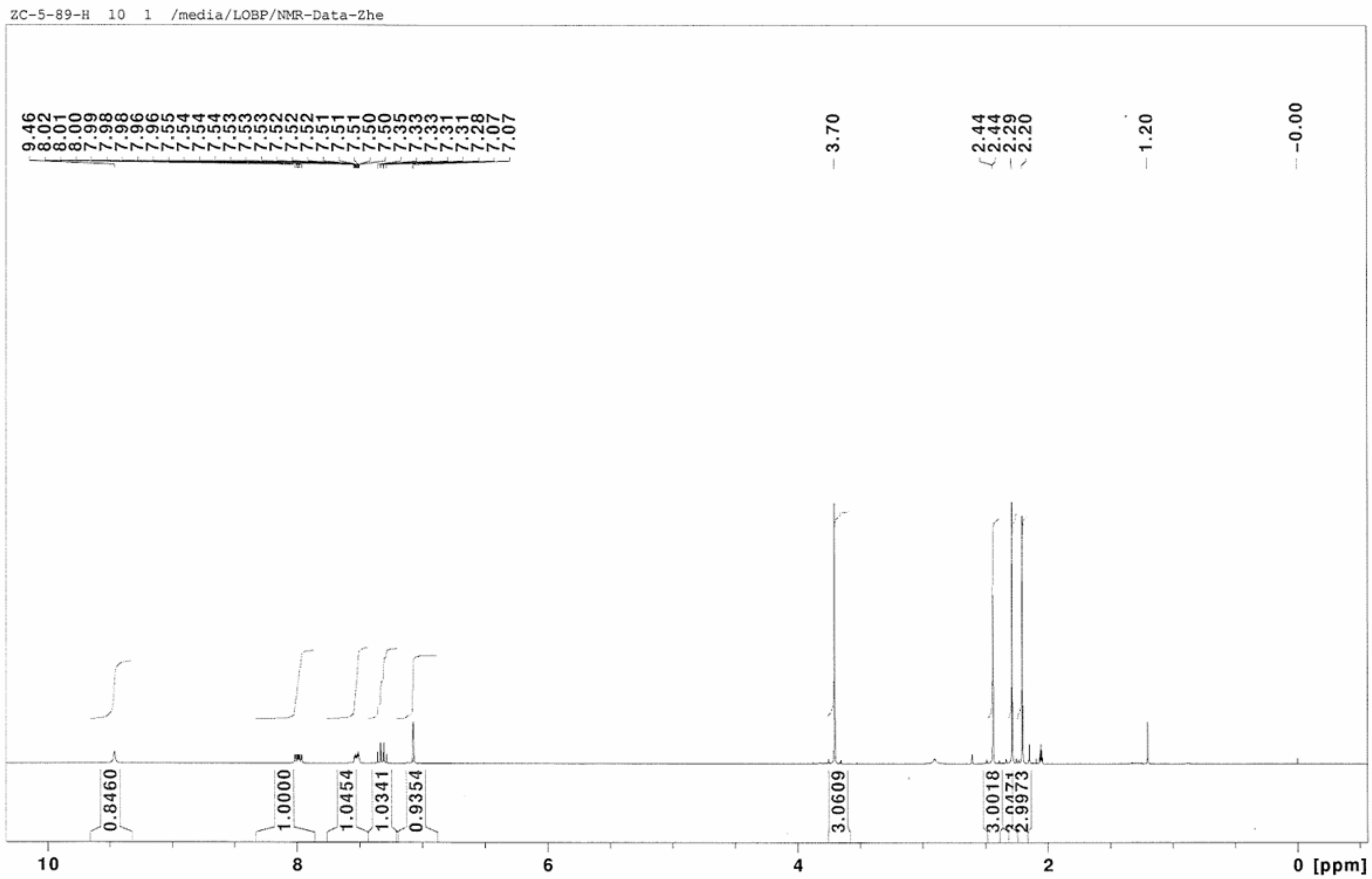

ZC-5-89-C 10 1 /opt/topspin3.2/ZC/ZC5/data/nmr00/nmr

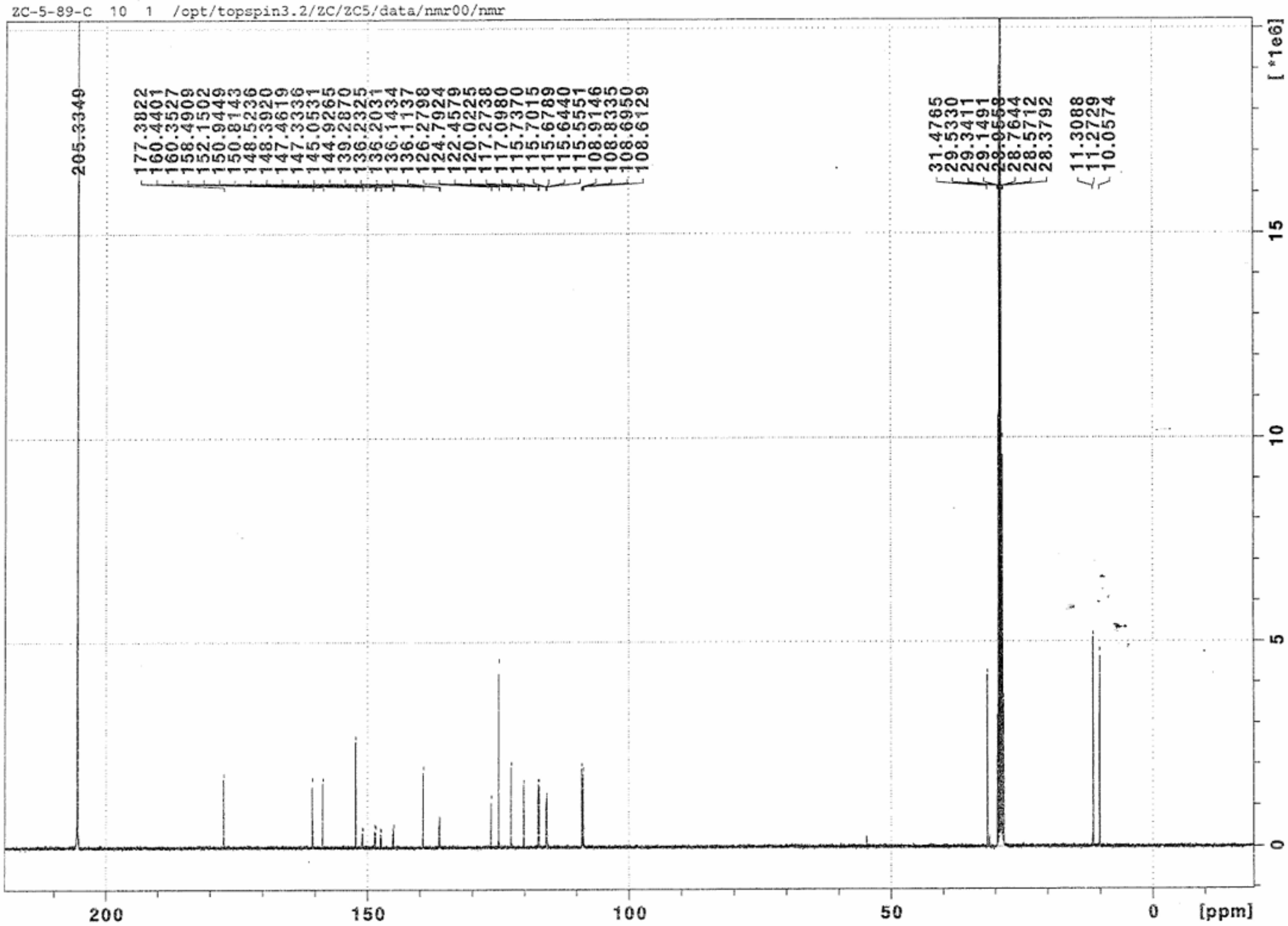

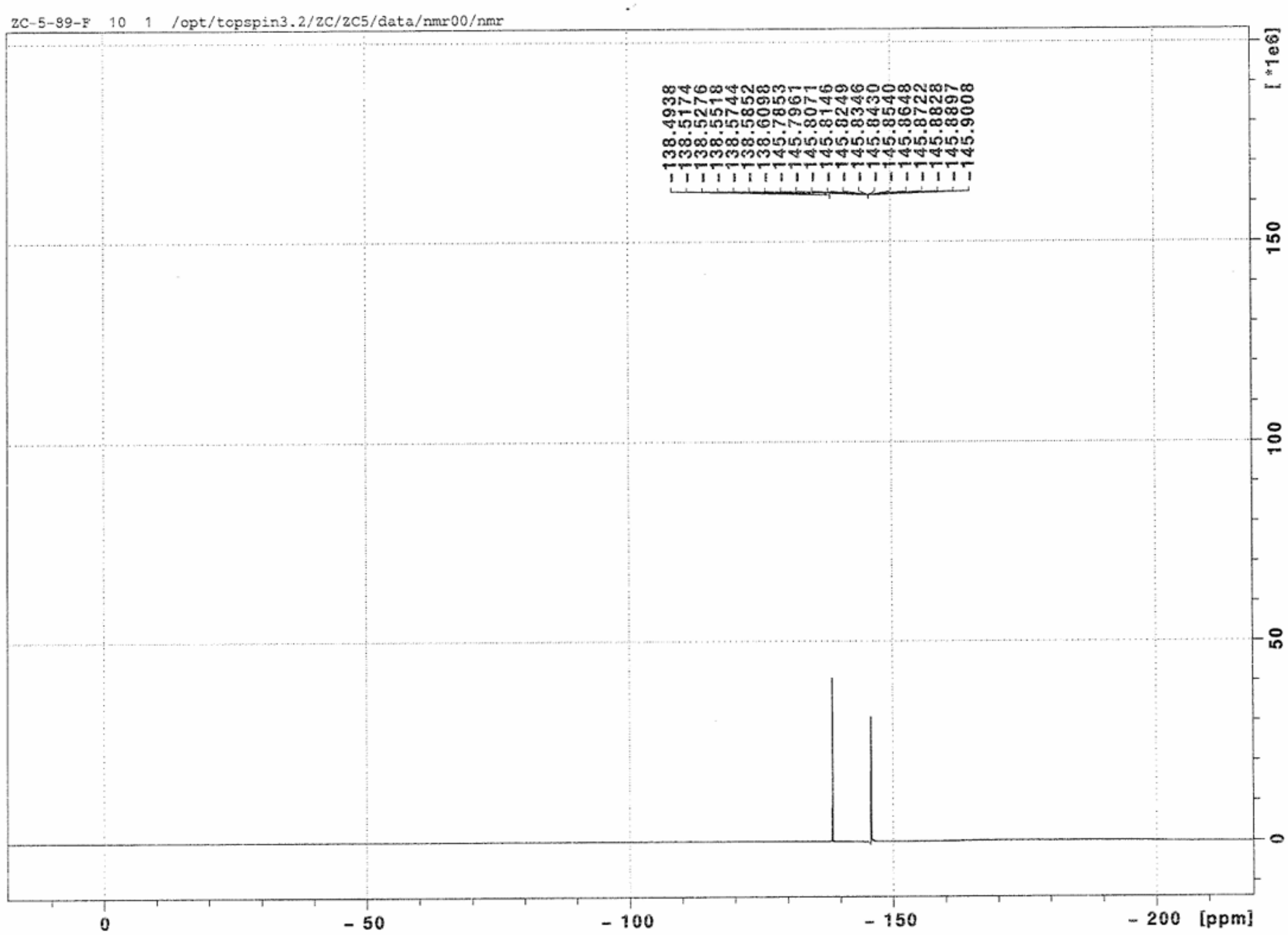
ZC-5-89-F 10 1 /opt/topspin3.2/ZC/ZC5/data/nmr00/nmr
-138.4938
-138.5174
-138.5276
-138.5518
-138.5744
-138.5852
-138.6098
-145.7853
-145.7961
-145.8071
-145.8146
-145.8249
-145.8346
-145.8430
-145.8540
-145.8648
-145.8722
-145.8828
-145.8897
-145.9008
[*1e6]
150
100
50
0
0
- 50
- 100
- 150
- 200
[ppm]

NMR of compound 23

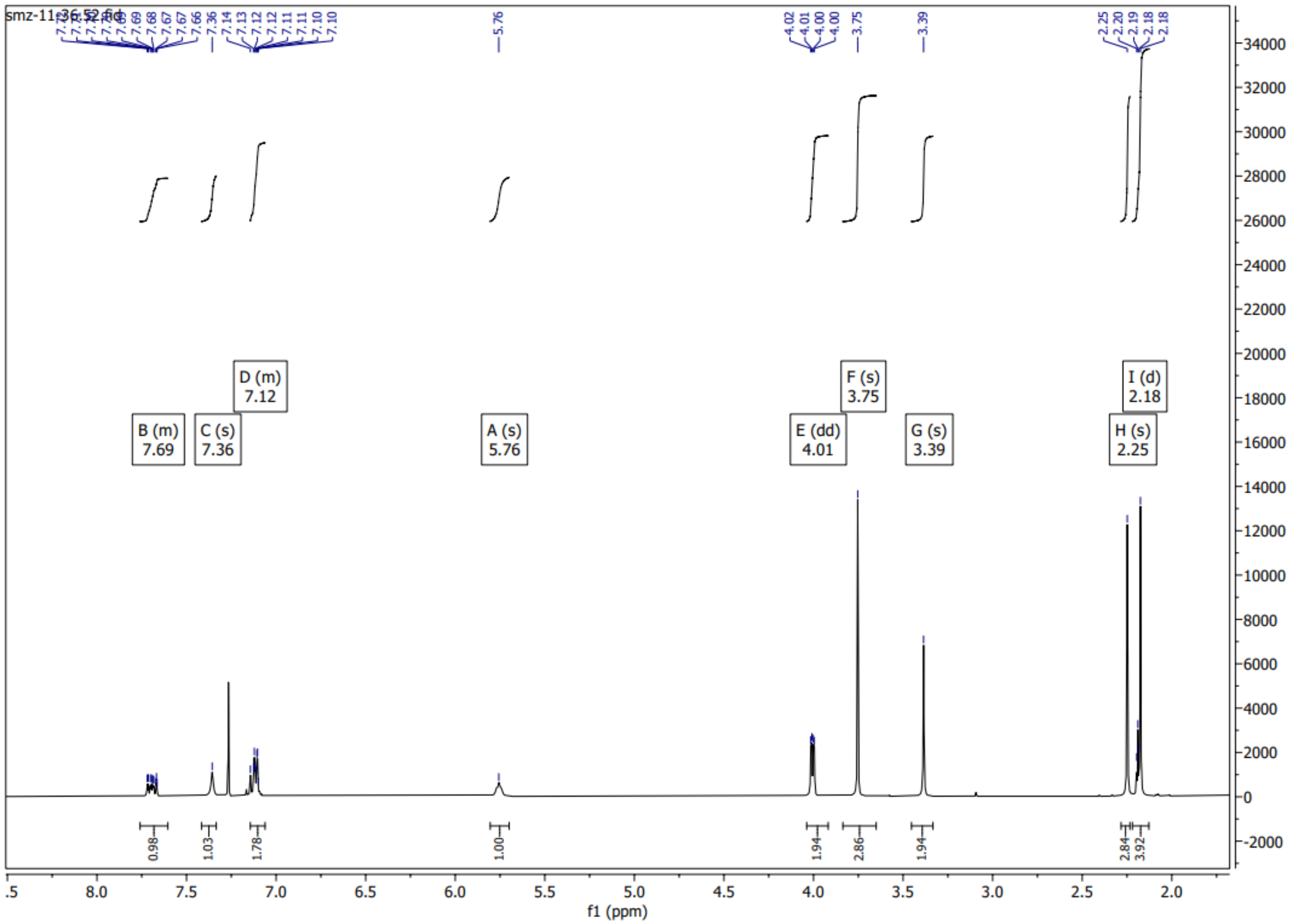

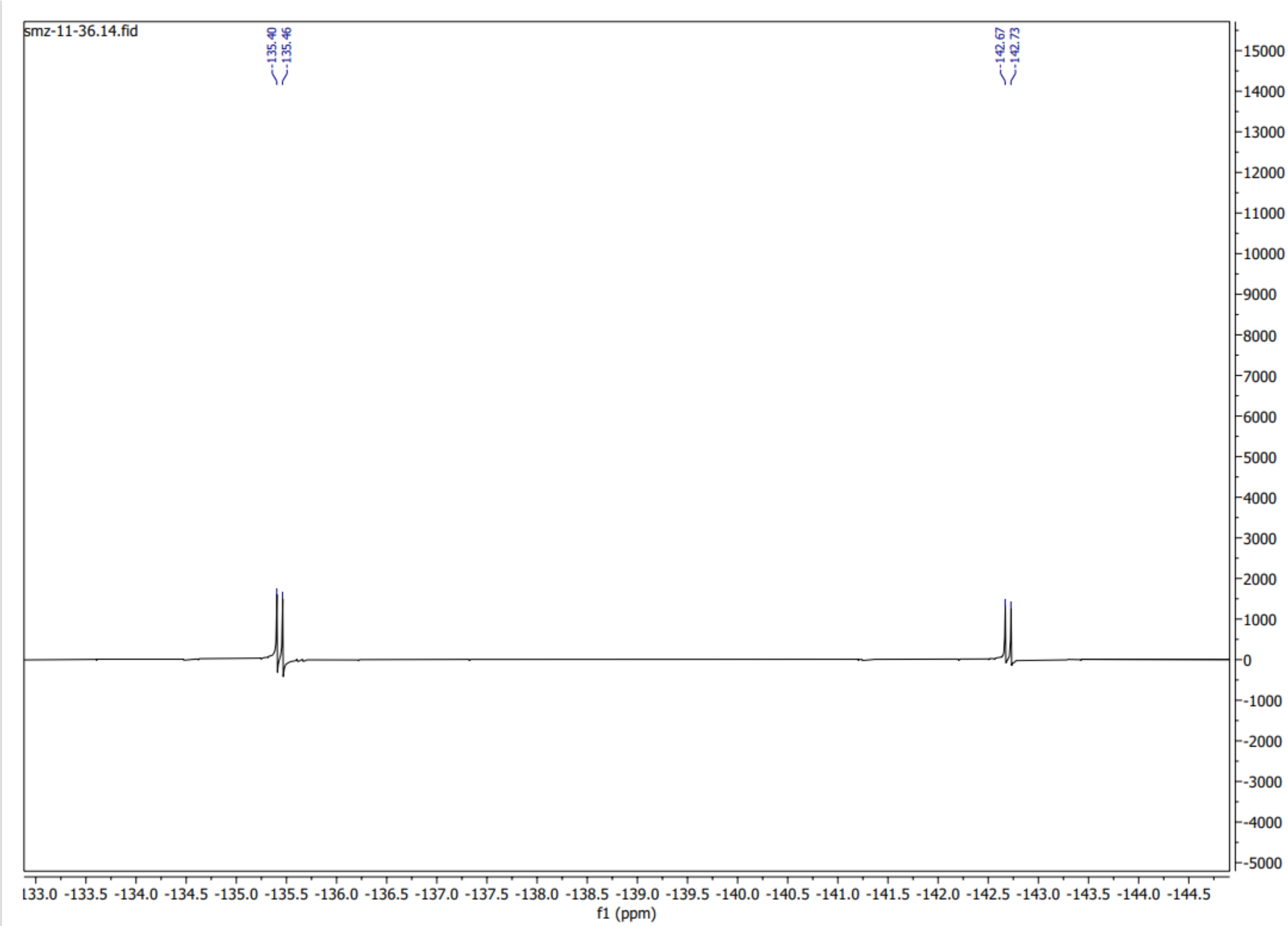

smz-11-36.14.fid
-135.40
-135.46
-142.67
-142.73
15000
14000
13000
12000
11000
10000
9000
8000
7000
6000
5000
4000
3000
2000
1000
0
-1000
-2000
-3000
-4000
-5000
133.0 -133.5 -134.0 -134.5 -135.0 -135.5 -136.0 -136.5 -137.0 -137.5 -138.0 -138.5 -139.0 -139.5 -140.0 -140.5 -141.0 -141.5 -142.0 -142.5 -143.0 -143.5 -144.0 -144.5
f1 (ppm)

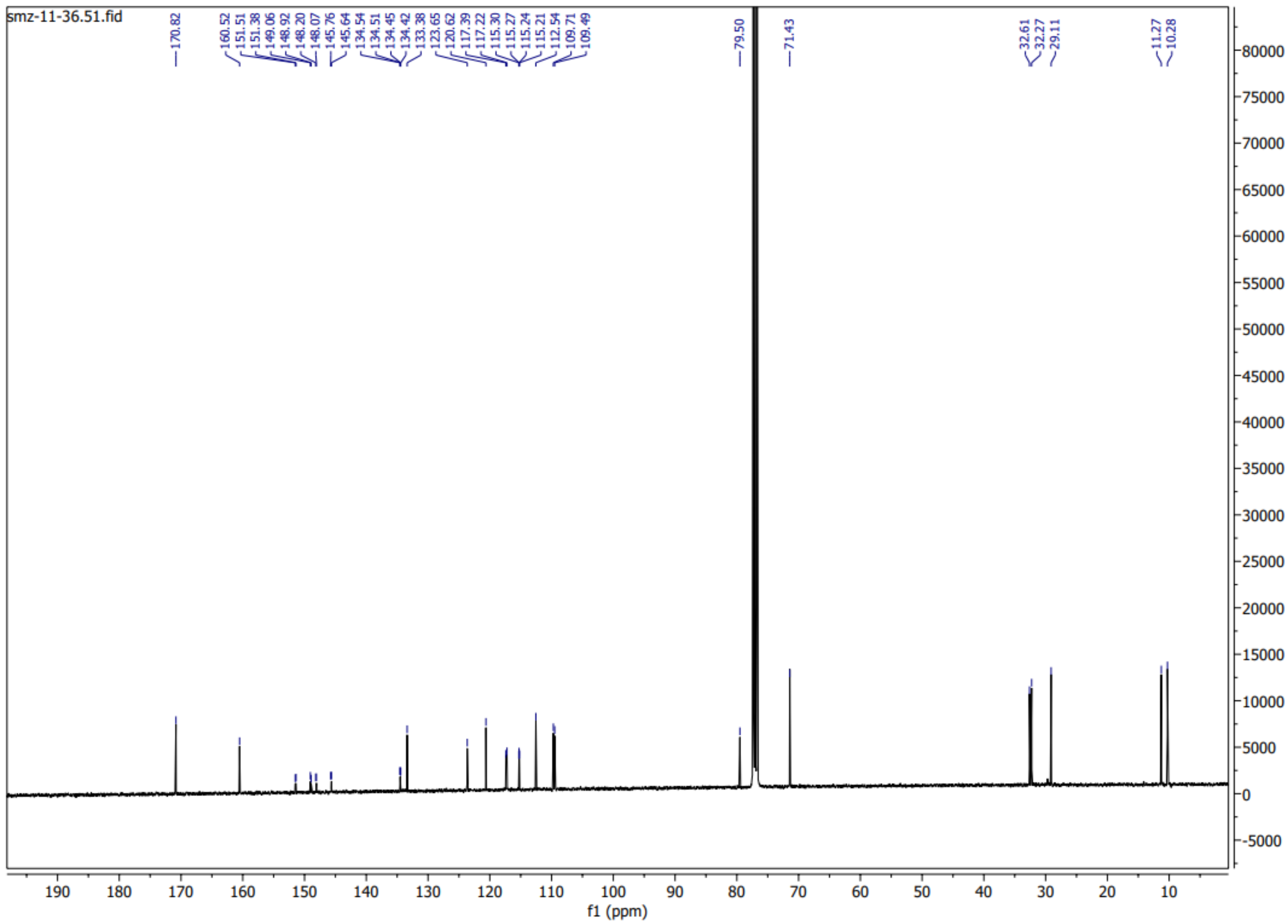

smz-11-36.51.fid
170.82
160.52
151.51
151.38
149.06
148.92
148.20
148.07
145.76
145.64
134.54
134.51
134.45
134.42
133.38
123.65
120.62
117.39
117.22
115.30
115.27
115.24
115.21
112.54
109.71
109.49
79.50
71.43
32.61
32.27
29.11
11.27
10.28
f1 (ppm)
